# Supplementary material for: A multi-agentic framework for real-time, autonomous freeform metasurface design
Source: Sci Adv. 2025 Oct 31;11(44):eadx8006. doi: 10.1126/sciadv.adx8006 (PMC12577674; doi:10.1126/sciadv.adx8006)
Supplement: 20251031-1 [file sciadv.adx8006.v1.pdf]

OPTICS

A multi-agentic framework for real-time, autonomous freeform metasurface design

Copyright © 2025 The Authors, some rights reserved; exclusive licensee American Association for the Advancement of Science. No claim to original U.S. Government Works. Distributed under a Creative Commons Attribution License 4.0 (CC BY).

Robert Lupoiu[1], Yixuan Shao[1], Tianxiang Dai[1], Chenkai Mao[1], Kofi Edée[2], Jonathan A. Fan[1]*

Innovation in nanophotonics currently relies on human experts who synergize specialized knowledge in photonics and coding with simulation and optimization algorithms, entailing design cycles that are time-consuming, computationally demanding, and frequently suboptimal. We introduce MetaChat, a multi-agentic design framework that can translate semantically described photonic design goals into high-performance, freeform device layouts in an automated, nearly real-time manner. Multistep reasoning is enabled by our Agentic Iterative Monologue paradigm, which coherently interfaces agents with code-based tools, other specialized agents, and human designers. Design acceleration is facilitated by Feature-wise Linear Modulation–conditioned Maxwell surrogate solvers that support the generalized evaluation of metasurface structures. We use freeform dielectric metasurfaces as a model system and demonstrate with MetaChat the design of multiobjective, multiwavelength metasurfaces orders of magnitude faster than conventional methods. These concepts present a scientific computing blueprint for using specialist design agents, surrogate solvers, and human interactions to drive multiphysics innovation and discovery.

INTRODUCTION

Optical metasurfaces control light through engineered diffraction, enabling a wide variety of advanced imaging (*1*, *2*), display (*3*, *4*), and sensing systems (*5*, *6*). The metasurface design process presents unique opportunities and challenges in scientific computing, as it leverages intricate relationships between the subwavelength-scale geometric structure and the electromagnetic response (*7*–*11*). Consequently, substantial efforts have been made to navigate this exponentially large design space (*12*–*19*). Conventionally, photonic design tasks in both academic and industrial settings involve subject experts who apply prior knowledge and experience to propose and simulate candidate device concepts (*20*). These approaches include heuristic algorithms that leverage physical insights and approximations to efficiently explore the photonic design space (*21*–*25*), as well as gradient-based methods that iteratively refine designs by optimizing a differentiable figure of merit (FoM) (*19*, *26*–*31*). While existing workflows have been effective at advancing the field, they require extensive time and resources to train personnel, ideate, code, and execute full-wave simulation and design tasks. Furthermore, the need for nanophotonics expertise to perform advanced metasurface design has presented challenges for researchers in adjacent research fields to incorporate detailed metasurface concepts into their workflows.

To this end, deep learning algorithms have been touted as next-generation tools for transforming and streamlining the computer-aided design process in the physical sciences, including in photonics (*32*, *33*). In the realm of mathematical computing, neural networks have been developed to solve partial differential equations using architectures ranging from physics-informed neural networks (*34*–*38*) to Fourier neural operators (*39*–*42*), and they have been used to effectively perform end-to-end and iterative freeform optimization tasks (*43*, *44*). For example, surrogate deep network solvers and optimizers have been demonstrated to accurately evaluate Maxwell's equations and determine structure-function relations with orders of magnitude faster speeds (*43*, *45*, *46*). There have also been notable efforts to develop and integrate large language models (LLMs) within the scientific computing pipeline. Using text-based descriptions of photonics device structures and their electromagnetic behavior, LLMs have been used to directly simulate relatively simple photonics devices, such as predicting the spectra of periodic metasurfaces (*47*, *48*) and multilayer thin film stacks (*48*, *49*). While LLMs fine-tuned on spectral regression can perform inverse design tasks, such as thin film stack optimization (*48*), this process is costly and data-intensive compared to purpose-built deep learning architectures (*47*) and faces limitations with more complex structures, like periodic elliptical metasurfaces (*47*). Thin film structure optimization has also been demonstrated using an LLM decoder-only transformer architecture that outputs multilayer thin film structures on the basis of optical design target inputs (*49*). Instead of directly simulating electromagnetic behavior with an LLM, orchestration frameworks leverage the inherent nanophotonics intelligence of LLMs to call surrogate solvers and other tools, which can be more physically rigorous and unconstrained by the requirement of a token-based interface, to perform more complex simulation and design tasks. These frameworks have demonstrated using LLMs to support the semantic interpretation of designer needs (*50*–*52*), fuse scientific knowledge with the design process (*48*, *50*, *53*), and assist in detailed coding tasks (*52*). To date, LLM frameworks have been used to assist in tasks in a diverse range of physical science topics, such as the design of materials (*50*), optical fibers (*51*), and laser cavities (*52*).

While there has been remarkable progress in augmenting photonics design with the data sciences, it is challenging to extend the functionality of these concepts toward nontrivial use cases. Deep learning–based mathematical computing algorithms remain highly specialized and cannot account for the diversity of physical parameters required to describe practical photonic systems. LLM design frameworks for physical sciences largely serve as planners or wrappers with user-friendly interfaces (*51*, *52*). These are built around the decomposition of problems into logical steps and the preplanned use of external tools (Fig. 1A) (*50*, *51*), leading to the rigid and

[1]Department of Electrical Engineering, Stanford University, Stanford, CA 94305, USA. [2]Institut Pascal, CNRS, Clermont Auvergne INP, Université Clermont Auvergne, F-63000 Clermont-Ferrand, France.
*Corresponding author. Email: jonfan@stanford.edu

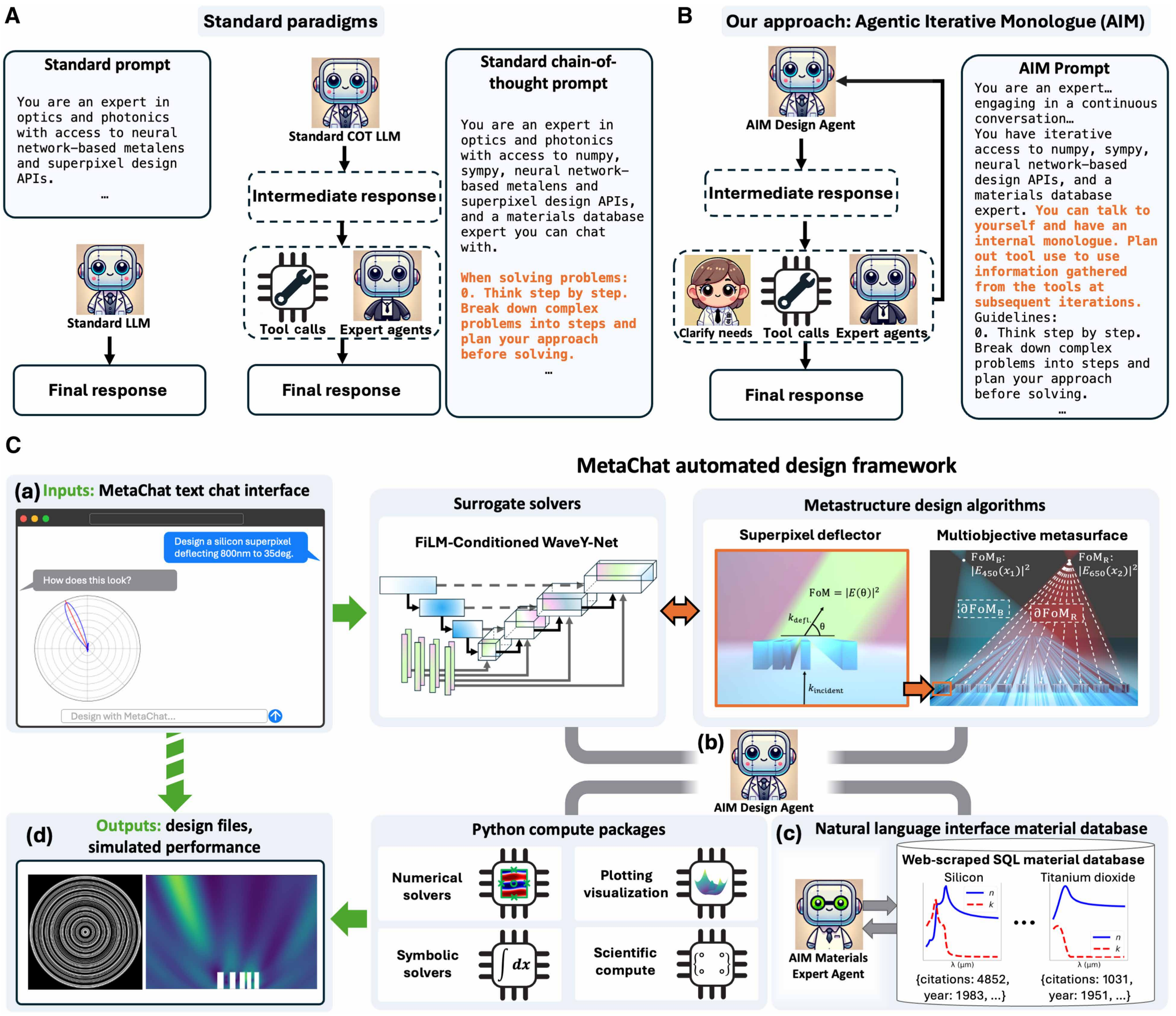

Fig. 1. MetaChat agentic framework for autonomous metasurface design. (**A**) Standard paradigms for creating LLM assistants. (Left) Standard prompting instructs the LLM to assume a certain role for each response, which is generated directly from a given input query. (Right) In COT prompting, the LLM is further instructed to explicitly break down the input request into multiple steps before responding, which structures its internal reasoning to help maintain coherence for complex queries. External tool calls can be invoked in a noniterative, single-shot manner. (**B**) The AIM prompting strategy creates agents that refine their intermediate responses through self-dialogue, autonomously leveraging feedback from external tools, agents, and the human designer at multiple stages to optimize the final answer. (**C**) Overview of the MetaChat framework. AIM Design (b) and Materials Expert (c) Agents iteratively interface with high-speed metasurface optimization and design algorithms, Python computing packages, and the human user to process semantic user inputs (a) into outputted metasurface design files (d) in nearly real time.

sequential execution of LLM-generated instructions. While many of these tools claim to be agentic, they do not constitute a design framework featuring true agency, which embodies intentionality, forethought, self-regulation, and self-reflectiveness (*54*). Thus, they lack the capability of acting on intermediate thoughts or autonomously adapting actions on the basis of feedback from interactions with tools, other agents, or the human user.

We present a multi-agentic computer-aided design framework, called MetaChat, which combines true agency with millisecond-speed deep learning surrogate solvers to automate and accelerate photonics design. We show that MetaChat is capable of performing complex freeform design tasks in nearly real time, as opposed to days to weeks required by the manual use of conventional computing methods and resources. This immense acceleration in scientific computing capability not only pushes the speed of innovation and design discovery to new levels but also enables MetaChat to rapidly experiment, autonomously course correct, and seek feedback from the user on quick timescales that permit practical, interactive human engagement. These agentic capabilities are enabled by a key concept that we propose, Agentic Iterative Monologue (AIM), an agentic system designed to seamlessly automate multiple-agent collaboration, human-designer interaction, and computational tools (Fig. 1B). We additionally propose a semigeneral full-wave surrogate solver, termed Feature-wise Linear Modulation (FiLM) WaveY-Net, which supports conditional full-wave modeling—enabling simulations with variable conditions, including source incident angle, wavelength, material, and device topology—while maintaining high fidelity to the governing physics. We focus MetaChat on the

design of two-dimensional dielectric metasurfaces, although the concepts can be readily adapted and generalized to other photonic device domains. With MetaChat, we demonstrate the automated design of multiobjective, multiwavelength metalenses and deflectors with efficiency levels that match or surpass those previously reported in the literature.

A more detailed overview of MetaChat is shown in Fig. 1C. MetaChat consists of an AIM Design Agent, which performs detailed metasurface design, and an AIM Materials Expert Agent, which is specialized in optical material properties and can perform candidate material identification. The agents autonomously engage with the human designer through a chat-like interface, with each other, with a variety of external tools, and with themselves through self-thought. In this way, they each continually update their course of action on the basis of intermediate results and feedback. The AIM Design Agent uses a suite of application programming interfaces (APIs) to leverage FiLM WaveY-Net and gradient-based freeform optimization algorithms. The AIM Design Agent also has access to a coding environment equipped with compute packages commonly used by human designers during the design and validation process. These packages include scientific computing tools capable of rigorous numerical and matrix-based calculations, symbolic solvers that enable equation manipulation and analytical solving capabilities, conventional numerical Maxwell solvers, and visualization packages that generate graphical outputs for human designer interpretation. The Materials Agent is capable of generating context-dependent responses to questions that typically have multiple viable solutions by using structured query language (SQL) to traverse an information-rich database, overcoming the limitations of traditional lookup table approaches that would suffer from the one-to-many problem.

RESULTS

Agentic autonomous nanophotonics designers

The heart of MetaChat is the AIM paradigm for creating LLM-driven agents that excel at scientific reasoning and design (Fig. 1B). AIM treats each of its outputs as an intermediate thought by default, from which external tool interactions, conversations with other agents, and dialogue with human users are iteratively and autonomously self-invoked using output tags. AIM builds on ReAct and related frameworks (*55*, *56*), which interleave reasoning within a chain-of-thought (COT) trajectory with actions involving external tools but which are constrained by a rigid response format that diminishes the agent's ability to interact fluidly with human users and other agents. AIM is particularly well suited as an agentic design assistant because it is capable of refining and correcting outcomes on the basis of external feedback and is able to converse with expert agents and the human user equally naturally.

AIM derives its agency from a carefully designed prompt, which is presented in Materials and Methods and detailed in the Supplementary Materials. AIM is distinguished from other agentic frameworks (*55*, *56*) by its unconstrained outputs that are each treated as an intermediate iterative monologue thought of the agent, with any tool use or chats with other agents being mediated by HTML-like tags. This recursive monologue loop continues until it is broken with a self-outputted response tag, which channels the final response to the user. Within this iterative process, AIM realizes the core factors of true agency (*54*). Like basic assistant LLMs, intentionality is achieved by assigning a clearly defined, purpose-driven identity, in this case as an expert assisting users with optics and photonics problems. Forethought is realized using established COT prompting techniques, which explicitly instruct the LLM to think step by step and break down complex problems into a plan before solving. AIM achieves the final two conditions for agency in a flexible manner that is compatible with human designer collaboration by imposing the default state behavior of internal monologue. Self-regulation is achieved via the built-in feedback loop that enables the agent to monitor and adjust its processes through tool and optimization output analysis. Self-reflectiveness is fulfilled by the internal dialogue through which the agent assesses its own reasoning to refine its decision-making trajectory. AIM's intrinsic self-regulation, facilitated by iterative self-reflection, enables precise agent-driven multistep scientific computing, circumventing the rigidity associated with executing predetermined plans lacking intermediate result feedback (*50*, *51*) or requiring granular human expert intervention throughout the decision-making process (*57*).

AIM-enabled MetaChat is implemented via a full stack application architecture that integrates a local front-end interface for human designers, a local back end for orchestrating business logic, an LLM server, and a graphics processing unit (GPU)–accelerated machine learning server for hosting ancillary neural networks, such as surrogate solvers. The block diagram in Fig. 2A illustrates the sequentially numbered information flow within MetaChat. The process starts with initiation of the agentic workflow by the user with a query into the local front-end user interface. The query is passed to the Design Agent in the local back end and appended to its context, which consists of the full conversation history among the user, the Design Agent, and other agents, along with a prompt instructing the LLM about its photonics design assistant persona, available tools, and expected behavior. The Design Agent context is then passed to the LLM server, where next-token prediction iteratively constructs the response string (*58*, *59*). The response includes tagged placeholders, which are programmatically parsed to facilitate Design Agent interactions with other agents, coding tools, and ancillary neural networks, such as surrogate solvers via API calls. The Design Agent has the ability to iterate on the results with itself, other agents, and tools until it is ready with a response that is passed back to the front-end user interface, where it is processed into a human-readable format. The response also includes hyperlinks to the back-end database where any solutions are stored.

To evaluate the performance of our AIM designer, we establish and use the Stanford nanophotonics benchmark. This benchmark was developed because of the lack of standardized evaluation metrics for photonics design, and it comprises 101 diverse problems in photonics, electromagnetics, and optical engineering distributed in five categories: direct calculation, multistep calculation, material search, direct function call, and multistep function call (Fig. 2B). Each problem is carefully designed to test framework capabilities in a standalone, autonomous environment without human designer input. Database-specific problems, such as element counting or citation reporting, are an important part of the benchmark because they evaluate the multi-agentic capability of MetaChat's Design Agent to delegate problems correctly to the Material Expert Agent and the latter's proficiency in generating accurate SQL queries. The distribution of the problems is visualized in Fig. 2C by plotting the first two UMAP (uniform manifold approximation and projection) dimensions of the question string embeddings (*59*, *60*). Several clusters of common problems present in the dataset emerge that are commonly

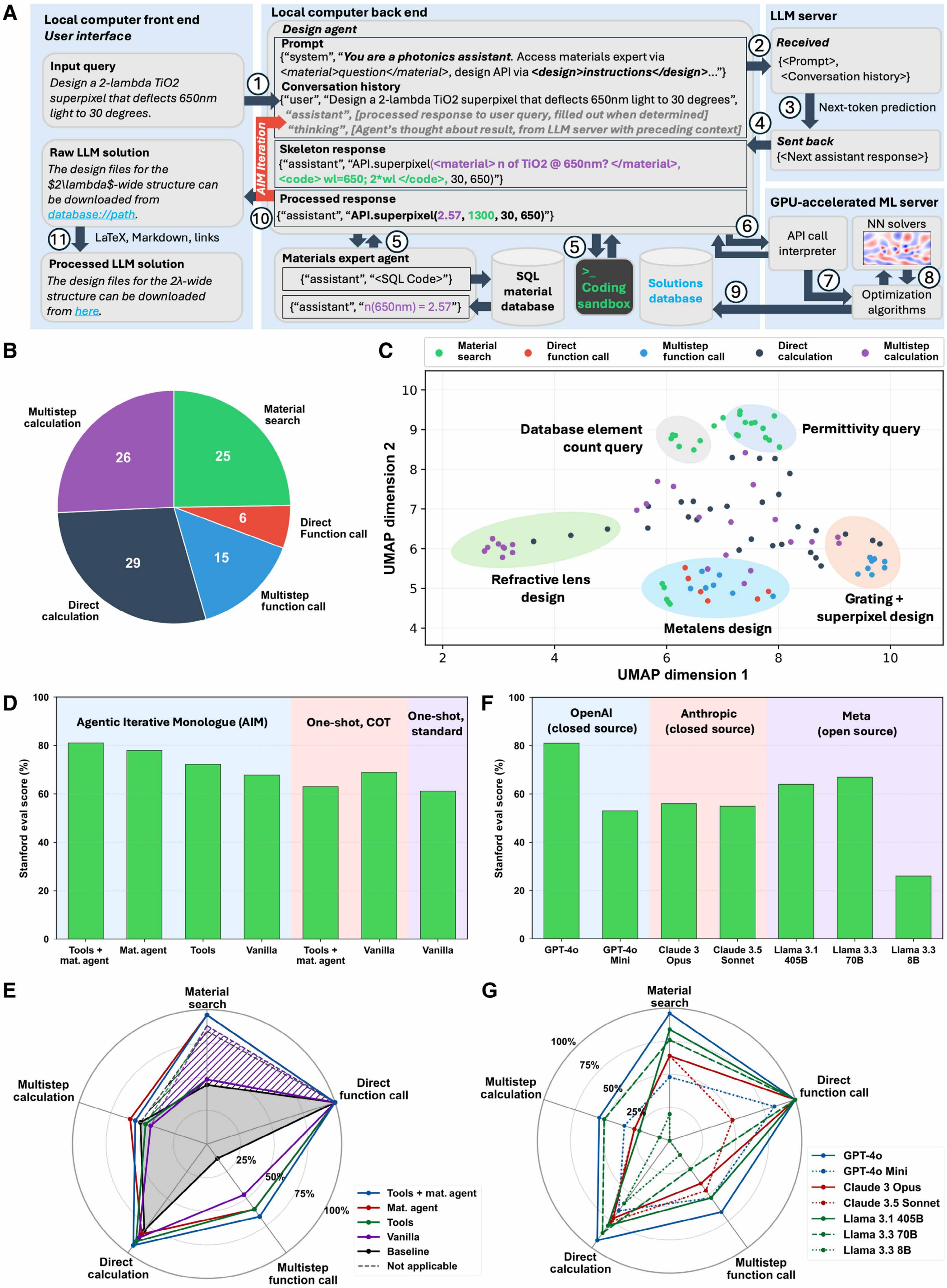

Fig. 2. Photonics design agent evaluation on a graduate-level nanophotonics knowledge and optimization benchmark. (**A**) Block diagram detailing the sequentially numbered flow of information in a MetaChat query. User queries are submitted through the local front end and processed by the back end with the assistance of local databases and coding tools. The back end communicates with externally hosted LLM and GPU-accelerated servers for natural language processing and near–real-time numerical optimization, respectively. (**B**) Problem category distribution in the Stanford graduate student–generated benchmark. (**C**) 2D UMAP projection of all 101 problem embeddings. Each plotted problem is color coded on the basis of its benchmark category. (**D**) AIM and LLM assistant ablation study using the Stanford nanophotonics benchmark. Green bars indicate the percentage of correct answers. (**E**) AIM ablation study across the five problem categories of the Stanford nanophotonics benchmark. AIM, equipped with both tools and access to the Materials Expert Agent, achieves the highest scores consistently across all five categories, with the most substantial improvement observed in the multistep function call category. (**F**) AIM LLM driver performance evaluation using the Stanford nanophotonics benchmark. Bars indicate the percentage of correct answers, with the best-performing LLM being the closed-source GPT-4o (81%). (**G**) AIM LLM driver performance evaluation across the five problem categories of the Stanford nanophotonics benchmark. GPT-4o is the best-performing model, and it is the only one to achieve consistently high scores across all five categories.

encountered by nanophotonics engineers, including metalens design and material permittivity queries. The center of Fig. 2C comprises 37 points that represent general photonics engineering problems that cannot be grouped with a particular commonly encountered class. The structured question-answer format of the benchmark enables the comprehensive and quantitative assessment of the performance of agentic frameworks and their underlying LLMs.

We first use the evaluation benchmark to quantify the performance of AIM with respect to alternative assistant LLM approaches and to assess the utility of MetaChat's supporting tooling via ablation studies (Fig. 2, D and E). GPT-4o is used as a common LLM backbone. In this assessment, database element count query questions are withheld because of their specificity to the Materials Agent Database, but we note that the AIM Materials Expert Agent is able to address these problems with 100% accuracy. The baseline score of a vanilla LLM assistant that outputs an answer directly (Fig. 1A, left) is 61.1%. Through the vanilla COT prompting strategy (*61*), without access to any external tools or the Materials Agent, the score increases to 68.9%. Unexpectedly, this score drops to 63.0% after tools and Materials Agent access are added to the COT assistant (Fig. 1A, right). This drop in performance is attributed to the requirement for a more complex prompt containing tools and Material Agent use instructions, which the one-shot LLM cannot leverage to its advantage because of diminished instruction-following performance with longer prompt size and errors arising from tool interactions on queries the LLM could otherwise answer directly.

Our AIM designer (Fig. 1B), on the other hand, is able to take advantage of external tools and agents to more accurately solve problems. The base score of AIM without any external helper tools or Materials Agent access is 67.8% and comparable to that of vanilla COT despite the fact that our AIM designer uses a substantially longer and more complex prompt than that used for the vanilla COT assistant. Its performance further improves after adding either tools (72.2%) or the Materials agent (78.0%), and with both combined external resources, the AIM designer achieves a maximum score of 81.0%. With access to both tools and the Materials Agent, the AIM designer consistently achieves high scores across all five categories of the evaluation benchmark (Fig. 2E). AIM outperforms ablated versions and baseline measures most in the multistep function call category of benchmark problems (Fig. 2E), indicating a higher propensity to maintaining long-term coherence in the face of multistep calculations and function calls. In particular, the agentic approach succeeds in cases where the preplanned, one-shot COT assistant encounters tool-calling errors during multistep function and API calls because of AIM's ability to interpret error messages and feedback to dynamically adjust its strategy and to iteratively retry interactions with external tools. These results underscore the importance of the self-regulation property of a truly agentic system, which is a capability not present in rigid preplanning or wrapper-type approaches to interfacing with design tools.

To evaluate the impact of LLM type on AIM designer performance, we perform our evaluation benchmark on seven different state-of-the-art LLM models. This assessment is important to perform for our application, as most LLM model benchmarks focus on single-shot, direct responses that are reflective of more typical chatbot-type tasks that are grounded by a human at each step (*62*, *63*). In contrast, our AIM designer has particular demands in instruction following, function calling, context size, and long-term coherence for multistep scientific computing task automation. The results are summarized in Fig. 2 (F and G). We determine that OpenAI's closed-source GPT-4o outperforms all tested models. Meta's open-source Llama 3.3 70B model achieves a score of 67.0%, which comes the closest to the state-of-the-art score achieved by GPT-4o. These results indicate that model size is not predictive of agentic driver performance. We further find that performance does not track with commonly well-regarded LLM benchmarks. For example, Anthropic's closed-source Claude 3.5 Sonnet, which is widely regarded as a top-performing LLM model (*62*), performs substantially worse than GPT-4o, Llama 3.1 405B, and Llama 3.3 70B. In addition, Anthropic's models consistently underperform both GPT-4o and Llama 3.3 70B in more complex multistep function calls, as well as in multistep and direct calculations (Fig. 2G). These observations indicate the importance for our AIM agents to interface with LLM models that support long-term coherence and excellent function-calling capability to agentically achieve correct answers. We hypothesize that the better-performing LLMs in Fig. 2F have a relative overrepresentation of function call and numerical calculation examples in their respective training processes.

Ultrafast electromagnetic simulations with FiLM WaveY-Net

The high-speed surrogate full-wave solver is the mathematical computing engine driving MetaChat. New deep learning architectures are required, as existing direct deep learning surrogate Maxwell solvers are too specialized and unable to support generalized capabilities to the degree required for practical MetaChat functionality. We propose FiLM WaveY-Net, a semigeneral full-wave solver of metasurface superpixels that supports the ultrahigh-speed simulation of freeform aperiodic dielectric metasurfaces (Fig. 3A). Superpixels are defined to be wavelength-scale metasurface scatterer sections (Fig. 3B) that can be stitched together to produce a large area, functional freeform metasurface (*28*, *64*). Compared to strategies based on the stitching of meta-atoms, our superpixel strategy accounts for near-field coupling between neighboring nanostructures and their arbitrary positioning, making it ideal for freeform metasurface design tasks. To train FiLM WaveY-Net, 270,000 examples of ground truth training data are produced using a finite-difference frequency domain (FDFD) simulator (*65*). The superpixels comprise dielectric ridges placed over a glass substrate, and perfectly matched layer (PML) boundary conditions are used at all sides of the simulation domain (Fig. 3B). The superpixel parameters are uniformly sampled from the ranges listed in Fig. 3C, with half of the dataset positioning the illumination source below the superpixel structure within the glass substrate and the other half above the structure in air.

The FiLM WaveY-Net architecture builds on previous surrogate frequency domain full-wave solver architectures (*43*, *45*, *46*, *66*), where it was shown that convolutional neural networks could be trained to accurately simulate dielectric metasurface structures but which were limited to highly specific input source conditions. With FiLM conditioning (*67*), the source wavelength and incident angle are inputted as explicit scalar inputs and are converted into feature-wise affine modulation parameters via learned linear transformations. These parameters modulate intermediate feature maps at each decoding block in the base UNet architecture. Spatial information about the source position is provided through two additional input channels corresponding to the real and imaginary components of the source, alongside the original dielectric structure channel, to the convolutional neural network. A key feature of FiLM conditioning that enables generalized solving capabilities is that it enables the

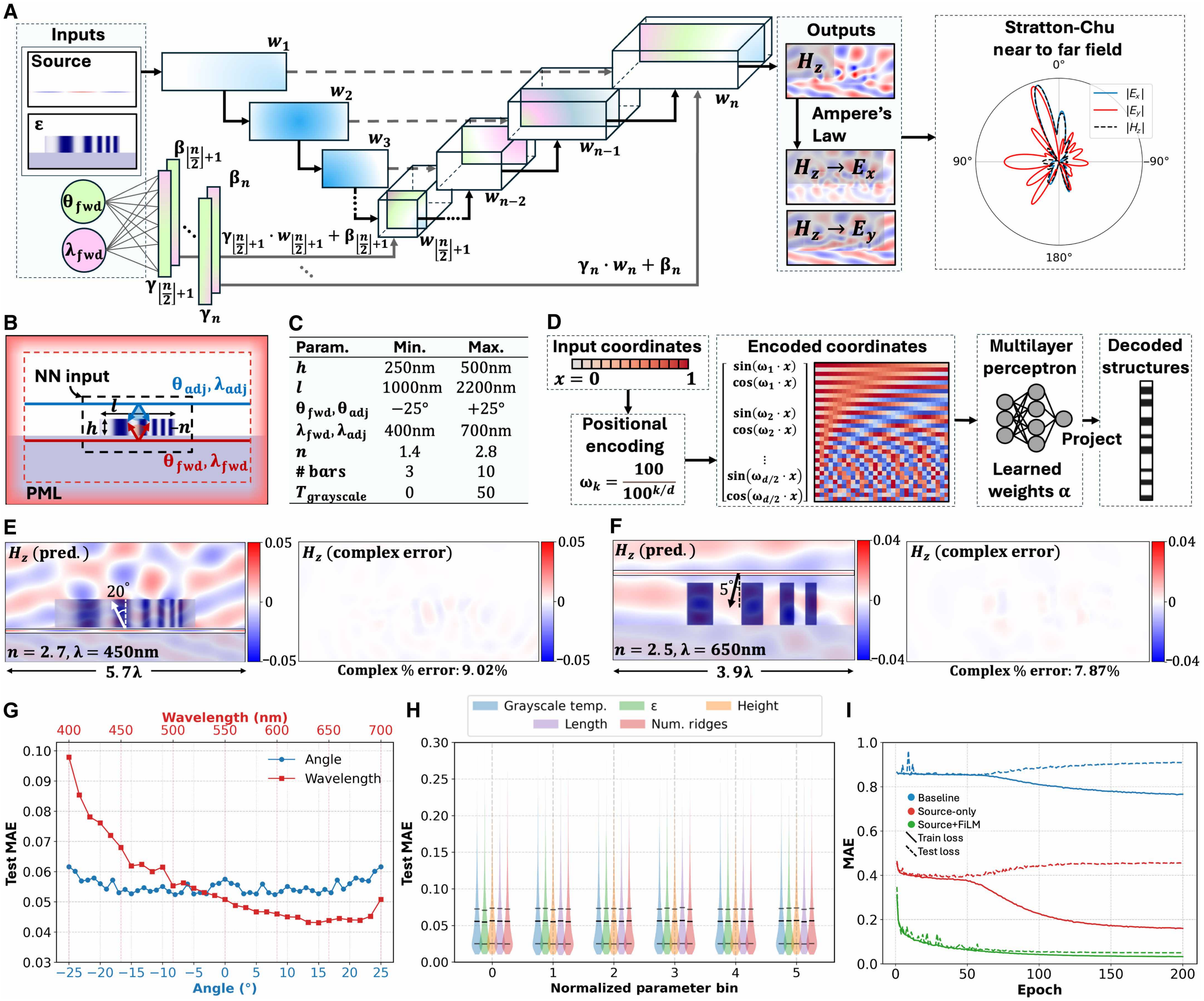

Fig. 3. FiLM WaveY-Net full-wave surrogate solver with support for variable sources and structures. (**A**) FiLM WaveY-Net is composed of a UNet backbone augmented by learned source parameter–dependent affine transformations that modulate its decoding blocks. The Stratton-Chu method computes far-field scattering profiles from the outputted full-wave near fields. (**B**) FiLM WaveY-Net data simulation domain. The red dashed line indicates the PML boundaries. The black dashed line indicates the center section of the domain input to the network. The solid red line indicates the location of forward sources, and the solid blue line indicates the location of backward sources. (**C**) Ranges of the variable quantities in the FiLM WaveY-Net training set. (**D**) Overview of the differentiable neuroparameterization framework for defining superpixel geometry. The input spatial coordinates x are mapped into a higher-dimensional space using rotary positional encoding. The encoded coordinates are then passed through a multilayer perceptron, which learns an implicit SDF representation of the design. (**E** and **F**) Samples of FiLM WaveY-Net surrogate simulation results (left) and complex percentage error between the predicted and ground truth magnetic fields (right). (**G**) Test loss as a function of the variable source parameters: incidence angle (blue) and wavelength (red). (**H**) Violin plot distributions of test loss across the full range of structure-related input parameters: grayscale temperature (blue), maximum permittivity (green), height (orange), length (purple), and number of ridges (red). Each parameter range is divided into six bins for direct comparison. The bottom quartile, median, and top quartile are indicated with black horizontal lines. (**I**) FiLM WaveY-Net ablation study. The "Baseline" model inputs the structure only, the "Source-only" model inputs the source along with the structure, and the "Source+FiLM" model uses the FiLM conditioning technique in conjunction with the source and structure inputs to achieve a low loss that generalizes to the test set.

central convolutional block's encoded dielectric structure representation to be adaptively decoded into a source-specific field response. Functionally, FiLM conditioning facilitates precise source dependency by effectively projecting generalized decoding parameters onto distinct hyperdimensional subspaces that are unique for each illuminating source. We use FiLM conditioning to extend our surrogate solving capabilities to the accurate evaluation of a wide range of device heights, binary and grayscale dielectric material configurations, device topologies, illumination wavelengths, and illumination angles (Fig. 3C).

FiLM WaveY-Net can be configured within a fully differentiable end-to-end pipeline for the simulation of freeform superpixels with

customized far-field profiles, enabling the use of autodifferentiation to perform freeform gradient-based superpixel design. Superpixel layouts are parameterized using a neuroparameterization approach illustrated in Fig. 3D, which encodes the geometry in a high-dimensional neural signed distance function (SDF) representation. This approach enforces geometric constraints, such as minimum feature size, without excessively limiting the optimization space. To enforce geometric constraints, geometric loss terms are integrated into the optimization process and guide adjustments to the superpixel geometry through gradient backpropagation, thereby leveraging the differentiable nature of the neural SDF. Implementation details are provided in Materials and Methods and will be further analyzed in future studies. Evaluation of the superpixel electromagnetic response is subsequently performed with FiLM WaveY-Net, which outputs the magnetic near-field response, and the electric near fields are computed using a discretized form of Ampere's law. The complex scattering far-field response based on these near fields is evaluated using Stratton-Chu formalism (Fig. 3A, right). The neural networks, Ampere's law, and the Stratton-Chu calculations are all fully differentiable, enabling the use of gradient-based optimization via backpropagation to iteratively modify the superpixel geometric layout in a manner that pushes its complex far-field response toward a desired pattern.

An evaluation of FiLM WaveY-Net showcases its generalizability and an accuracy level that is sufficiently high for inferred fields to be directly used in gradient-based optimization algorithms. An inspection of two randomly sampled structures and illumination conditions is shown in Fig. 3 (E and F), illustrating the quality and low error level of representative outputted fields. A more systematic evaluation of FiLM WaveY-Net using a test set of 30,000 examples provides quantitative performance metrics. The angle-dependent test performance exhibits a steady normalized mean absolute error (MAE) averaging 0.055, with a slight increase to 0.062 at the extremity of the training set (Fig. 3G). This level of consistency indicates that varying the source angle does not pose variability in training difficulty for FiLM WaveY-Net. The representation of wavelength data in the training set is dynamically tuned on the basis of the wavelength-dependent test performance during training to account for variability in learning difficulty, which is due to the increase in spatial information from higher source frequency illumination. Nonetheless, the final test MAE exhibits an inverse relationship with respect to wavelength, ranging from 0.098 to 0.043 across the 400- to 700-nm range (Fig. 3G). The test performance is consistent across the variable dielectric structure parameters, with each parameter exhibiting the same mean of 0.06 normalized MAE across its entire parameter range and with 90% of samples exhibiting a loss under 0.10 (Fig. 3H).

Ablation studies highlight the essential role that our FiLM conditioning mechanism plays in ensuring robust network generalization to diverse source conditions (Fig. 3I). In the baseline evaluation without any explicit conditional input, the vanilla WaveY-Net architecture (*43*) is incapable of learning this ill-posed, one-to-many training task (Fig. 3I, blue traces). We also perform a benchmark evaluation in which source information is explicitly included in the channel input, making the training problem well posed. Nonetheless, this straightforward conditioning method is insufficient and the resulting trained networks exhibit substantial training set overfitting (Fig. 3I, red traces). Related conditioning strategies in which more explicit, learned feature maps are injected into the network also fail to generalize beyond the training set, as detailed in the Supplementary Materials. FiLM WaveY-Net is qualitatively better and accurately maps conditional fields to corresponding input structures and sources without overfitting (Fig. 3I, green traces).

Autonomous design of multiwavelength, multiobjective metasurfaces

The programming interfaces that link the MetaChat AIM agents with FiLM WaveY-Net are APIs that are tailored to translate a desired device function to a set of optimization objectives and full-wave solver calls. For this study's demonstrations, we limit the APIs to multifunctional metasurfaces serving as deflectors and lenses, although the design and API frameworks can readily extend to more arbitrary wavefront responses. Deflectors are specified to produce transmitted linear phase responses, while lenses are specified to produce transmitted parabolic phase profiles. While these optical functions are relatively basic, they are good model systems for our study, as it is highly nontrivial to effectively design efficient multifunctional devices with these capabilities. The API-enabled design strategy is shown in Fig. 4A. First, global metasurface phase responses are specified and quantified on the basis of the desired device function. In the case of multifunctional devices, sets of phase responses are specified for differing incident angles and wavelengths. Second, these spatial phase profiles are mapped to desired superpixel far-field scattering profiles, which stitch together to specify the full metasurface response. The corresponding global FoM to be minimized, which produces the desired phase responses, is the negative sum of the complex electric field amplitudes for each superpixel at points in the far field where intensity is maximal. Our approach streamlines and generalizes multiobjective metasurface design, as all tasks map onto the same superpixel scattering profile optimization approach.

Our global FoM can be readily decomposed into independent superpixel FoMs that each specify an individual scattering profile design task, thereby enabling the independent and parallel design of each superpixel. Details pertaining to individual gradient-based superpixel optimization are in Materials and Methods. Briefly, superpixels are initialized using our neuroparameterization framework and are specified as a random, high-temperature grayscale dielectric distribution. This distribution is evolved by iteratively evaluating the complex superpixel scattering profile using FiLM WaveY-Net and Stratton-Chu calculations and then using autodifferentiation and backpropagation to calculate gradients to the dielectric distribution in a manner that pushes the far-field response toward the desired profile. We use Adam gradient descent (*68*), and the temperature of the dielectric distribution is programmatically lowered over the course of optimization so that the final superpixels have discrete dielectric values.

Our gradient-based optimization method is ultimately a local optimizer that is sensitive to its initialization; thus, improved results can be obtained by optimizing batches of superpixels with differing initializations and selecting the best structure. Such a strategy aligns with the strength of FiLM WaveY-Net, which has high speed and leverages GPU-supported batch parallelization. A Monte Carlo sampling analysis of superpixel FoM as a function of batch size for a typical design task (Fig. 4B), where the best final FoM achieved within a batch is plotted, shows the inherent tradeoff between device performance and computing resources. This tradeoff can be explicitly accounted for by the Design Agent depending on human designer needs.

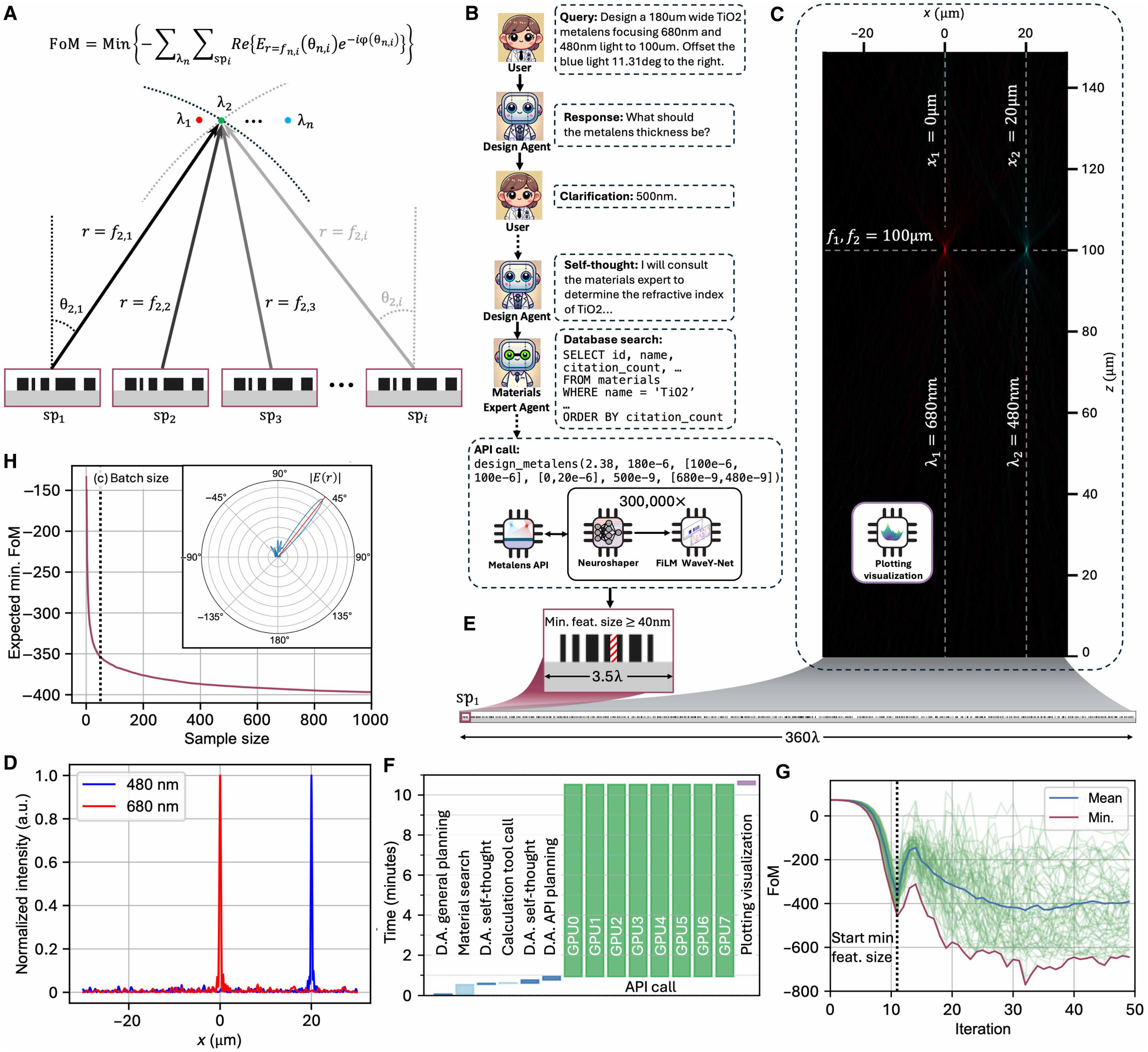

Fig. 4. Automated multiobjective metalens design via MetaChat. (**A**) Design strategy for metasurfaces using superpixel arrays. The FoM captures electric field magnitudes at the desired focal points, which is maximized by the constructive interference of complex scattering fields from all superpixels. (**B**) Expected minimum FoM versus sample size based on Monte Carlo sampling from 1000 optimization runs. The inset depicts the best superpixel far-field scattering profile (target amplitude: red line) from a batch size of 60, showing minimal side lobes. (**C**) Salient speech excerpts from the design conversation between the user, Design Agent, and Materials Expert Agent for the design of a dual-wavelength, multifunctional metasurface spanning 360 wavelengths. The Design Agent asks for clarification before engaging in self-thought and autonomous conversation with the Materials Expert Agent before executing the design process. (**D**) Final metalens design, comprising 100 superpixels that are each ~3.5 wavelengths wide. a.u., arbitrary units. (**E**) MetaChat visualization tool metalens simulation. The horizontal dashed line indicates the focal plane; vertical dashed lines indicate designed lateral shifts. (**F**) Normalized intensity in the metalens focal plane versus lateral position, featuring a 480-nm peak at 20 μm and a 680-nm peak at 0 μm. (**G**) Execution time profiling of the design process. Design Agent iterations are in dark blue, and tool calls are in light blue. The API compute is in green, and plotting visualization is in purple. This entire MetaChat design process takes 11 min, with the 300,000 FiLM WaveY-Net simulations accounting for 10 min. (**H**) Sixty parallel optimization trajectories for one of the metalens superpixels. The start of minimum feature size enforcement is indicated via the vertical dashed line. The trajectory that leads to the best-performing structure that is ultimately included in the metalens design is the red curve. The mean FoM is the blue curve.

To illustrate how the AIM Design Agent interfaces with our general API design framework, we task MetaChat to design a dual-wavelength metalens (Fig. 4C). We input the following plain language design query: "Design a 180 μm wide TiO2 metalens focusing 680nm and 480nm light to 100um. Offset the blue light 11.31deg to the right" (Fig. 4C, top). The Design Agent queries the user for missing thickness constraint information, followed by a string of self-thoughts that lead to a Materials Expert Agent query. This chain of actions culminates in an API call that sets off 300,000 GPU-parallelized FiLM WaveY-Net simulations for the gradient-based optimization of a set of 100 superpixels (Fig. 4C, bottom). Each of the superpixels spans 1.8 μm in length and is constrained to support a default 40-nm minimum feature size, enforced by our neuroparameterization method (Fig. 4D, top). Once the superpixel optimization process is completed, the superpixels are stitched together to form a fully aperiodic metalens (Fig. 4D, bottom). With MetaChat's visualization tools, the metasurface far field is plotted using the angular spectrum method (Fig. 4E). As designed, the focal plane is situated at 100 μm, with the 680-nm focal spot centered and the 480-nm focal spot offset by 20 μm. The intensity along the focal plane is plotted in Fig. 4F and shows that the 680- and 480-nm peak lobes contain 57.1 and 49.7% of their respective total far-field energy scattering profile. These results compare favorably with the performance of similar devices from previously reported related demonstrations (*28*, *69*).

The timing analysis for the entire agentic design process of the metalens is presented in Fig. 4G. The agentic interactions, including self-thinking and Materials Expert conversation, are complete within the first minute. With the computing resources allocated for this design problem, eight GPUs are used in parallel to complete the required 300,000 simulations in 10 min, after which plotting the final result is complete within 30 s. A similar metalens would take a knowledgeable practitioner 5.03 days in compute time alone using a classical FDFD simulation approach on an 80-thread server (*65*). A plot of the FoM of 60 optimizations as a function of iteration number for a representative superpixel type is shown in Fig. 4H and shows the wide variance in superpixel performance given random device initialization and the need to parallelize large optimization batches to identify suitably highly performing device structures.

In a second demonstration, we use MetaChat to optimize a multiobjective, large numerical aperture metalens operating at red, green, and blue (RGB) wavelengths with the query in Fig. 5A. The 540-nm green focal point is instructed to be centered, and the 670-nm red and 450-nm blue focal points are instructed to be offset by 30 μm to the left and right, respectively. The focal plane is specified to be 100 μm above the device for each spot. The metalens is specified to be 200 μm wide and is decomposed into 111 superpixels. The far field of the optimized metalens is plotted in Fig. 5B, illustrating the design's success in shaping the wavefront of the three wavelengths into their intended focal points. The field intensity along the focal plane is plotted in Fig. 5C, indicating a state-of-the-art level of signal-to-noise ratio at the correctly positioned focal points (*28*, *69*).

Last, we demonstrate that MetaChat can be used to design highly directional multiobjective, multiwavelength deflectors. For example, a beam splitter with arbitrarily engineered directions (Fig. 5E, top) can be designed with the user query from Fig. 5D. The resulting field is simulated using FDFD (Fig. 5E, bottom), and the far-field intensity is plotted using the Stratton-Chu near-to-far field transformation (Fig. 5F), showing that the normally incident light is simultaneously deflected to 10° and 30°. MetaChat can readily generalize these design tasks for sources that are normally incident (Fig. 5, G and H) and obliquely incident (Fig. 5, J and K) onto the metasurface. Both of these examples exhibit the desired far-field behavior (Fig. 5, I and L) with favorable far-field efficiency levels compared to previously reported metasurface designs sharing similar objectives (*70*).

DISCUSSION

Nanophotonic devices, such as metasurfaces, have faced longstanding barriers to widespread adoption, limiting accessibility primarily to domain experts because of the specialized knowledge, computational demands, and rigid optimization schemes that are traditionally required (*20*). In this work, we introduce MetaChat, a multi-agentic design platform that lays the foundation for agentic automated photonic computer-aided design. MetaChat leverages AIM, a novel paradigm for LLM-driven agency, to automate this computationally intensive process that is traditionally reliant on domain-specific experts. On a graduate-level agentic design benchmark for photonics, AIM demonstrates long-range coherence across multistep design problems that includes diverse scientific computing tool use and fluid human designer interactions. We show that the AIM Design Agent within the MetaChat framework is capable of achieving best-in-class designs for multiobjective, multiwavelength metalenses and metasurface deflectors (*28*, *69*, *70*).

There are several promising avenues to further enhance MetaChat's capabilities. While our benchmark results demonstrate that prompt engineering is an effective strategy for AIM Agent development, fine-tuning LLMs with the domain-specific literature (*71*–*73*) can substantially improve the capabilities and accuracy of AIM for complex, multistep tasks. This is especially valuable in MetaChat-specific generalized reinforced policy optimization fine-tuning on long-term structured reasoning chains (*74*, *75*) with tool use in the loop. Increased agentic design autonomy can allow the agent to design optimal FoMs for achieving each unique user-queried design task. From a design interface perspective, multimodal LLMs can be integrated into the MetaChat framework to allow image-based instruction of the model to specify design preferences and constraints (*50*, *76*, *77*). We ultimately envision the expansion of our multi-agentic and modular MetaChat framework to an ecosystem of specialized agents that supports broader multiscalar design capabilities within optics and photonics. To name a few examples, such agents can specialize in and use differential models for ray tracing, global optimization, and fabrication constraints. We anticipate that through open-source initiatives in the photonics community (*78*, *79*), MetaChat's capabilities can be continuously evaluated, expanded, and tuned to reflect the evolving design needs of practitioners in industry and academia (*62*, *63*, *80*).

The concept of augmenting specialized, LLM-driven agents with ultrafast and physically accurate surrogate solvers presents substantial opportunities for accelerating progress in applied physics research in both industry and academia. For metasurfaces and nanophotonics in particular, MetaChat will particularly benefit adjacent research communities, ranging from astrophotonics and atomic physics to augmented and virtual reality (AR/VR), where there is broad interest but limited expertise in implementing detailed nanophotonic solutions. We anticipate even broader implications in the development and merging of ultrafast surrogate solvers with multi-agentic frameworks, where LLM agents can be used not only to generate novel scientific hypotheses (*81*) but also to modify these hypotheses in

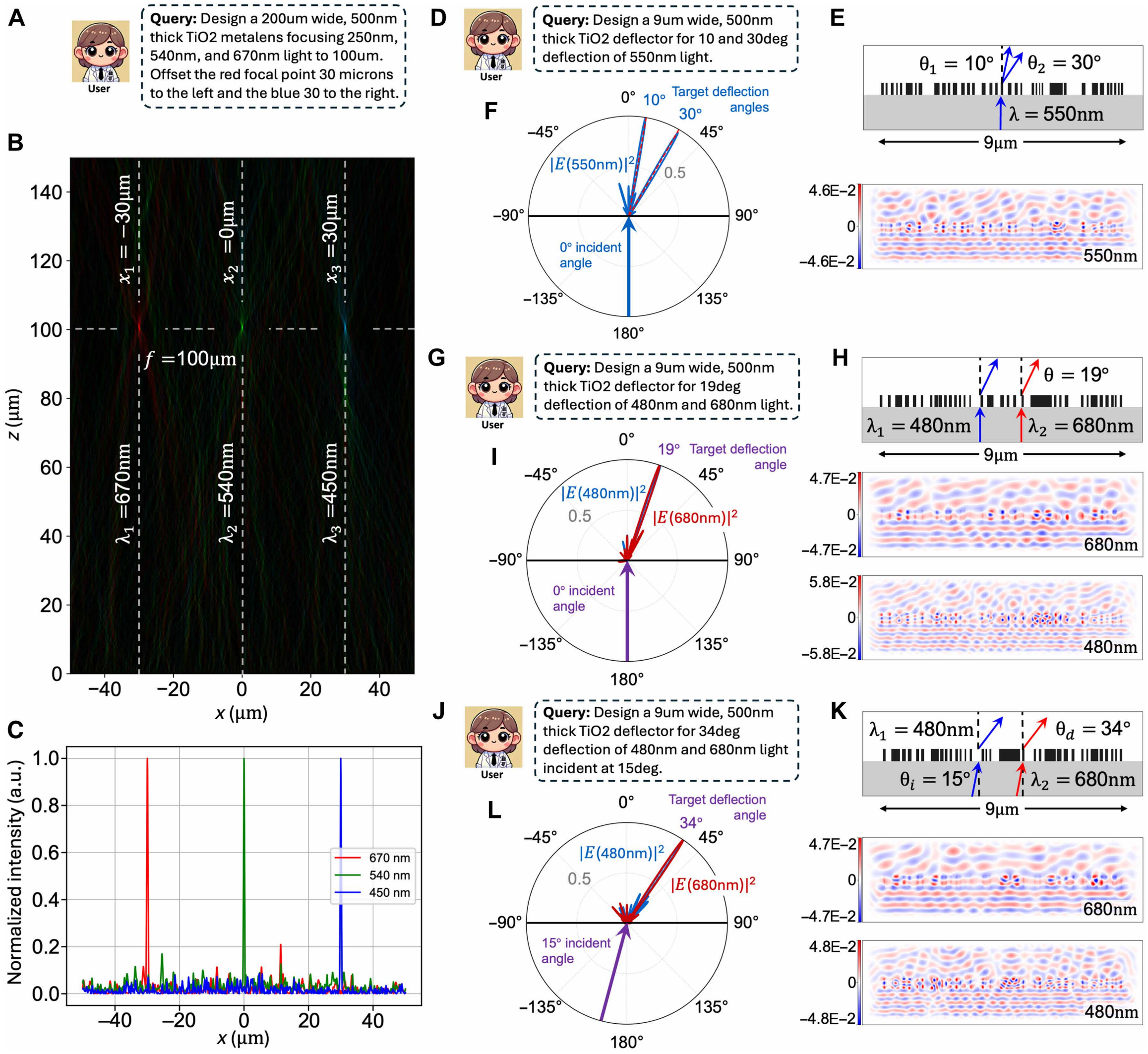

Fig. 5. Autonomous optimization of multiwavelength, multiobjective metalenses and deflectors. (**A**) Query for the MetaChat design of an RGB metalens with a 30-μm lateral shift between the three focal points. (**B**) Spectrally color-mapped far-field power profile of the optimized metalens, plotted using MetaChat visualization. The dashed horizontal line indicates a common focal length of 100 μm, and the dashed vertical lines indicate lateral positions of -30, 0, and 30 μm relative to the center for the red, green, and blue focal points, respectively. (**C**) Normalized intensity in the metalens focal plane as a function of lateral position for the device in (B). (**D**) Design query for a dual-peak, single-wavelength beam deflector with normal incidence. (**E**) (Top) 550-nm light normally incident on the five stitched binarized superpixels optimized via MetaChat to simultaneously deflect to 10° and 30°. (Bottom) Rigorous FDFD-calculated near-field response. (**F**) Far-field response of the five-superpixel deflector designed to scatter 550-nm normally incident light to 10° and 30°. (**G**) Design query for a dual-wavelength, single-direction beam deflector with normal incidence. (**H**) (Top) 480- and 680-nm light normally incident on the five stitched binarized superpixels optimized via MetaChat to deflect both wavelengths to 19°. (Middle and bottom) Rigorous FDFD-calculated near-field responses for both wavelengths. (**I**) Far-field response of the dual-wavelength five-superpixel deflector designed to scatter 480- and 680-nm normally incident light to 19°. (**J**) Design query for a dual-wavelength, single-direction beam deflector with oblique incidence. (**K**) (Top) 480- and 680-nm light obliquely incident at 15° on the five stitched binarized superpixels optimized via MetaChat to deflect both wavelengths to 34°. (Middle and bottom) Rigorous FDFD-calculated near-field responses for both wavelengths. (**L**) Far-field response of the dual-wavelength five-superpixel deflector designed to scatter 480- and 680-nm obliquely incident light to 34°.

real time. These concepts extend beyond photonics and can be leveraged in multiphysics applications where agents can dynamically interleave solvers for different applied science domains to design entire novel systems. Furthermore, surrogate solver-augmented agentic frameworks can be coupled with robotic experimental environments, enabling automated end-to-end optimization of design and experimental validation (*82–85*). This holds particularly exciting implications for the physical sciences as a whole, as it presents an unprecedented opportunity to push the boundaries of science using an infinite supply of ultrafast agentic intelligence.

MATERIALS AND METHODS

Stanford nanophotonics benchmark

The evaluation benchmark for assessing photonics design reasoning capabilities was developed collaboratively by four Stanford graduate students who specialize in metasurface and photonics device optimization, with authorship for each question attributed in the data bank provided in the Supplementary Materials. There are a total of 101 problems spanning photonics and optical design problems across five reasoning categories: direct calculation, multistep calculation, direct function call, multistep function call, and material search.

These are organized in a JSON file that includes metadata, the problem statement, expected approach, and the final answer. Evaluation is automated using a "grader LLM" powered by GPT-4o to ensure uniformity and objectivity in grading and completed using the pass@3 standard, as outlined in the Supplementary Materials (*86*).

The LLM versions and parameters tested in the LLM driver comparison study (Fig. 2F) are tabulated in Table 1. The temperature analysis study that determined that a temperature of 0.0 results in the highest performance is included in the Supplementary Materials.

Engineering of the AIM design agent

As outlined in Fig. 1B, the AIM Design Agent is composed of an LLM that is prompted to assume the role of a photonics expert and autonomously interact with the human user, tools, design APIs, and expert agents. The prompt is designed to implement AIM by providing instructions to treat each LLM inference as a self-thought that is fed back as an input in the next cycle, with other interactions requiring the explicit use of tags. For optimal performance, the prompt further features COT prompting that instructs the agent to break complex problems into manageable chunks and to think step by step before responding. In-context learning is also used, which provides explicit examples of how to use the tools and APIs the agent can interact with. The "production" prompt integrated into the full stack application outlined in Fig. 2A is provided in the Supplementary Materials. The AIM Design Agent prompt used for evaluation using the Stanford nanophotonics benchmark is presented in Box 1. Dall-E was used to generate the images depicting the agents and human designer in Figs. 1, 4, and 5. The prompt used was, "Generate an image of a scientist agent robot for a scientific study, square aspect ratio. Make it simple and friendly, with just the bust visible, not the legs as well. Use a rounded square for the head."

Engineering of the AIM materials expert agent

As illustrated in Fig. 1C(c), the Materials Expert Agent fulfills materials-specific questions by querying a SQL database containing material property information from refractiveindex.info. The database schema is detailed in the Supplementary Materials. The Materials Expert Agent prompt includes the schema, along with instructions on how to interact with the database through the SQL code. Information is also provided on how to use external functions for interpolating tabulated data on the basis of the formula type encountered in the database. These instructions are solidified using explicit examples as part of an in-context learning prompting strategy. The AIM Materials Expert Agent prompt is presented in Box 2.

Table 1. Comparison of AI (artificial intelligence) language models. This table presents a comparison of various AI language models, including their provider, release date, maximum token limit, and temperature range.

Model name	Provider	Version date	Temperature
GPT-4o	OpenAI	2024-08-06	0.0
GPT-4o Mini	OpenAI	2024-07-18	0.0
Claude 3 Opus	Anthropic	2024-02-29	0.0
Claude 3.5 Sonnet	Anthropic	2024-10-22	0.0
Llama 3.1 405B	Meta	2024-07-23	0.0
Llama 3.3 70B	Meta	2024-12-06	0.0
Llama 3.3 8B	Meta	2024-12-06	0.0

FiLM WaveY-Net architecture

The surrogate solver is constructed using a modified UNet architecture backbone because of its strong capabilities for pixel-to-pixel regression tasks in electromagnetics (*43*, *45*, *87*). The convolutional input accepts three channels: the dielectric structure map, the real source component, and the imaginary source component. The network's output consists of two channels: the real and imaginary components of the H_z field. The UNet backbone is composed of five downconvolution blocks, five upconvolution blocks, and the center encoded representation convolution block, with skip connections that transfer feature maps from the encoder blocks to the decoder blocks at the same resolution level. Each convolution block is composed of six layers, which consist of a convolution operation followed by batch normalization (*88*) and leaky ReLU (rectified linear unit) activation (*89*), with the feature map computed after the first convolution being added to the last layer via a residual connection (*90*). An initial kernel count of 16 is used, which is doubled at each resolution level. Max pooling is used for downsampling and nearest-neighbor interpolation for upsampling.

FiLM conditioning is applied to the center and upconvolution blocks, which are responsible for decoding the feature representation of the input into the corresponding field. There is one FiLM layer for each block, which is composed of a single fully connected layer that learns a mapping between the inputted source angle and wavelength values to parameter scaling, $\boldsymbol{\gamma}$, and shifting, $\boldsymbol{\beta}$, values. These are then applied to modulate the feature maps using

$$\text{FiLM}(\mathbf{x}) = \boldsymbol{\gamma} \odot \mathbf{x} + \boldsymbol{\beta} \quad (1)$$

where $\mathbf{x}$ is the feature map. In this way, the conditioned network is capable of explicitly modulating its decoding features on the basis of the parameters of the source illuminating the inputted dielectric structure.

Training data generation

Training data for FiLM WaveY-Net is generated using Ceviche FDFD (*65*). First, superpixels are randomly generated using the parameters outlined in Fig. 3C, with the reparameterization approach described by Chen *et al.* (*91*) to enforce minimum feature size constraints of 30 nm. The domain size is 350 by 500 voxels, with a resolution of 10 nm per voxel. A 60-pixel-wide PML boundary condition is used. The substrate is made of SiO_2, with a refractive index of 1.44. A total of 150,000 dielectric structures are generated. Two solutions are generated for each structure: one using a "forward" current source placed four voxels underneath the bottom of the superpixel and one using an "adjoint" current source placed 55 voxels above the substrate. In addition to the random angle and wavelength that both sources are assigned for each simulation, a random phase offset uniformly sampled from 0 to 2π is also applied to each simulation to simulate sources placed at arbitrary distances away from the superpixel. The transverse magnetic polarized simulation generates H_z, E_x, and E_y field components. Along with the permittivity distribution, ε, and the source map, J, are cropped in a 128 by 256–voxel window centered on the full superpixel simulation domain to form the neural network training data.

Box 1. AIM Design Agent evaluation prompt.

You are an expert in optics and photonics engaging in a continuous conversation to help users
↪ with their optics and photonics questions.
You have iterative access to numpy, sympy, neural network-based design APIs, and a materials database
↪ expert.
You can talk to yourself and have an internal monologue. You can talk to the user using <chat> tags.
↪ Plan out tool use to use information gathered from the tools at subsequent iterations.
Guidelines:
0. Think step by step. Break down complex problems into steps and plan your approach before solving.
1. If you need to ask the user for more information, use <chat> tags.
2. If calculations are needed, write Python numpy code between <tool>scientific_compute</tool> tags
3. If symbolic manipulation is needed to e.g. solve for a variable or rearrange an equation because you
↪ are unsure, write Python sympy code between <tool>symbolic_solve</tool> tags
4. Do not make assumptions based on common practices. If info is missing, ask the user for it using
↪ <chat> tags.
5. If you need to design a metalens or deflector, use these neural network tools:
IMPORTANT: Before beginning, check each parameter needed for the API call. If any info is missing,
↪ determine how you will figure it out. If you need user input, ask the user using <chat>
↪ up-front before continuing with anything else. DO NOT MAKE ASSUMPTIONS. ASK USING <chat>.
If a value is not provided, your next response must ask the user for it using <chat> tags. Do not
↪ make assumptions based on common values.
- For metalenses (1 to 2 wavelengths), use: <tool>neural_design
design_metalens(refractive_indices [list], lens_diameter [m], focal_lengths [list, m],
↪ focal_x_offsets [list, m], thickness [m], operating_wavelengths [list, m])
</tool>
Note: The focal_x_offset is the x-offset of the focal point from the center of the lens. All list
↪ parameters must have the same length (1 or 2).
- For deflectors, use: <tool>neural_design
design_deflector(refractive_index, length [m], incident_angle [deg], deflection_angle [deg],
↪ thickness [m], operating_wavelength [m])
</tool>
6. If you need information about materials or their properties, you can chat with the materials expert:
<tool>materials_chat
Your question or message to the materials expert
</tool>
7. Return the final answer wrapped in <chat> tags. Make sure your code prints the final answer in the
↪ correct units
8. If no calculations are needed, simply state the answer directly
9. You can only use ONE type of tag per message
10. Make sure to convert intermediate results to the correct units before using them to prevent
↪ multiplication or function unit mismatch errors

Continued

11. After using a tool, analyze its output before proceeding. If there is an error, think carefully why
↪ it occured and fix the code to try again.
IMPORTANT: Any text not wrapped in tags will be treated as your internal thoughts and planning. Only
↪ text within <chat> tags will be shown to the user.
Make sure your response makes sense to the user based on their last message.
Examples:
1. Use available tools:
- Scientific computing: <tool>scientific_compute</tool>
- Symbolic solving: <tool>symbolic_solve</tool>
- Neural design: <tool>neural_design</tool>
- Materials expert chat: <tool>materials_chat</tool>
2. Respond to the user (Without wrapping in <chat> tags the user will not be able to see your
↪ response!):
<chat>
Your final answer or response to the user
</chat>
3. For neural network design (return the text you receive so the user can run the API call):
<tool>neural_design
design_metalens(refractive_index=2.7, lens_diameter=100e-6, focal_length=200e-6, thickness=500e-9,
↪ operating_wavelength = 800e-9)
</tool>
Example workflow:
1. Think about approach:
This problem requires calculating X, then checking material properties...
2. Use tools as needed:
<tool>scientific_compute
import numpy as np
wavelength = 500e-9
freq = constants.c / wavelength
print(f'Frequency: {freq:.2e} Hz')
</tool>
For symbolic manipulation:
<tool>symbolic_solve
import sympy as sp
# Rearrange thin lens equation 1/f = 1/u + 1/v to solve for image distance v
f, u, v = sp.symbols('f u v')
eq = sp.Eq(1/f, 1/u + 1/v)
solution = sp.solve(eq, v)[0]
print(f'v = {solution}') # Should output: v = (f*u)/(u - f)
</tool>
3. Fix errors:
-The solution was returned in a different format than expected. I have to first access the dictionary
↪ in the list.
4. Chat with materials expert:
<tool>materials_chat
What materials would work well for X application?
</tool>
5. Provide final answer:
<chat>
Based on the calculations and material properties, the solution is...
</chat>

Box 2. AIM Materials Expert Agent prompt.

You are an expert materials scientist with deep knowledge of optics and photonics,
engaging in a continuous conversation to help users with their materials-related questions. You can
↪ query a
materials database through an iterative process to find suitable materials and their properties for
↪ specific applications.
{self.schema}
You can perform multiple SQL queries to build up understanding and refine recommendations. You can talk
↪ to yourself to have an internal monologue.
Each query should focus on a specific aspect of the investigation.
IMPORTANT NOTE: The wavelengths are given in micrometers. Convert units before querying.
IMPORTANT NOTE 2: The material name is the chemical formula. So "Crystaline Silicon" is name: Si, type:
↪ crystal
Conversation Guidelines:
1. Maintain a natural conversation while using database queries to support your responses.
2. Response Format:
- While gathering information: Use <query>, <interpolate>, or <calculate_n> to collect data
- When you have a FINAL ANSWER: Always wrap it in <response> tags
- You MUST eventually provide a response - don't get stuck in an infinite loop of queries
- Example flow:
1. Make queries to gather data
2. Once you have enough information, provide final answer:
<response>
Based on the data gathered, I recommend [material] because [reasoning]...
</response>
3. When you want to query the database:
REASONING: Explain why you're making this query
<query>
Your SQL query here (if searching for a material, you can pre-sort by selection criteria: citation
↪ count, year, etc.)
</query>
ANALYSIS: Brief analysis of the results and next steps:
- The material might not be stored as the exact string you search (e.g. BK7 has several variants,
↪ including "N-BK7" and "P-BK7" but there's no "BK7" in the database)
- If you need more info: State what additional query/calculation is needed. If there are too many
↪ results, refine the query until you have fewer than twenty (20) results. Use citation counts
↪ to find the best answer. Do not limit the results to twenty, just refine the query.
- If you have enough information: Provide final answer using <response> tags
IMPORTANT NOTE 3: If you use <query>, you cannot use <response> in the same message (you can only
↪ either query database or respond to user at once)
4. When you need to perform linear interpolation between two points:
<interpolate>
{{"x": target_value, "x1": first_x, "y1": first_y, "x2": second_x, "y2": second_y}}
</interpolate>

Continued

The system will return the interpolated value for further use.
5. When you need to calculate a refractive index using a dispersion formula:
<calculate_n>
{{"formula_type": X, "wavelength": wavelength_in_microns, "coefficients": [c1, c2,...]}}
</calculate_n>
The system will return the calculated refractive index value.
IMPORTANT NOTE 3: If you use <query>, <interpolate>, or <calculate_n>, you cannot use <response> in the
↪ same message.
6. Consider throughout the conversation:
- IF YOUR QUERY RETURNS MULTIPLE RESULTS, use citation counts and publication years to choose the
↪ data source. More/more comprehensive data or different formats do NOT necessarily make a
↪ source more reliable.
- IMPORTANT: If there are multiple results, use citation counts and publication years to find the
↪ best answer BEFORE interpolating
- Interpolate between measurements if necessary for finding properties at specific wavelengths
↪ between measured points
- Application constraints
Remember to:
- Use queries only when needed to support the discussion
- Ask clarifying questions when necessary
- Provide clear and concise explanations
- Build upon previous conversation context
- Use interpolation when precise values are needed between measured points
You are engaging in an ongoing conversation, so don't try to solve everything at once. Break down
↪ complex
questions into manageable parts and use queries strategically to support the discussion.

FiLM WaveY-Net training

The WaveY-Net class of physics-augmented neural operators (*43*) adopt the Yee grid formalism (*92*) to verify the self-consistency of the fields using the governing Maxwell's equations. This framework precisely defines the spatial relationships between the discretized field components of numerical simulations, ensuring stable behavior for the discrete spatial derivative operations of Maxwell's equations. In this framework, the H_z field vector for each voxel is placed in the center, with the parallel E_x and E_y field components placed at the voxel boundaries. Using this formalism, we calculate the Helmholtz equation for the H_z field to construct a Maxwell loss that tracks the physical validity of the neural network's outputted field

$$\mathcal{L}_{\text{Maxwell}} = \frac{1}{N}\sum_{n=1}^{N} \left\| \nabla \times \left(\frac{1}{\varepsilon_n} \nabla \times \widehat{H}_n \right) - \omega^2 \mu_0 \widehat{H}_n \right\|_2^2 \tag{2}$$

where N is the batch size, and $\widehat{H}_n$ is the H_z field predicted by FiLM WaveY-Net for the inputted dielectric map ε_n.

FiLM WaveY-Net is trained using a loss function composed of both data and Maxwell loss components

$$\mathcal{L} = \mathcal{L}_{\text{data}} + \alpha \mathcal{L}_{\text{Maxwell}} \tag{3}$$

$$\mathcal{L}_{\text{data}} = \frac{1}{N}\sum_{n=1}^{N} \|\widehat{H}_n - H_n\|_2^2 \quad (4)$$

where H_n is the ground truth H_z field, and α is a factor that is set to 0 on the first training epoch and then dynamically scaled thereafter at the beginning of each epoch to the ratio of the last epoch's average data loss to the average Maxwell loss.

The versions of FiLM WaveY-Net used for ablation studies in Fig. 3I and in the Supplementary Materials are trained using 30,000 simulated examples with a 90/10 train/test set split. The final version of WaveY-Net used for benchmarking and optimization throughout the rest of the study is trained using a pool of 150,000 samples that are dynamically selected from a total of 270,000 samples on the basis of the wavelength-dependent validation loss. After each training epoch, the test set is evaluated and divided into bins every 10 wavelengths across the 400- to 700-nm training range. The sampling rate for selecting data from each of these bins to create the next epoch's training set is set to the ratio of each bin's test loss to the sum of all bins' test losses. In this way, wavelength ranges that perform more poorly on the validation set become proportionally overrepresented in the training set, which assists with equalizing the validation loss across wavelengths because of the inherent difficulty of learning higher-frequency field solutions. The test set is composed of 30,000 held-out simulation samples. All training is completed using an Adam optimizer over 200 epochs with a batch size of 64 and a starting learning rate of 3×10^{-4}, with an exponential per-epoch decay of 0.99.

Stratton-Chu near-to-far field formulation

A previous surrogate solver optimization study (*91*) relied on the angular spectrum method to perform near-to-far field transformations, but this was determined to be insufficiently accurate for capturing the far-field phase and amplitude information of the aperiodic superpixels optimized in this study. The Fraunhofer approximations of the angular spectrum method for a stitched metalens begin to break down for aperiodic systems. We thus implement the more rigorous Stratton-Chu near-to-far field formalism (*93*) that applies a Green's function transformation on the near field.

Stratton-Chu applies a near-field bounding surface over which fields are integrated for projection into the far field using Green's functions. In our simulation, this surface is a rectangle placed three voxels away from the neural network output's perimeter. The far fields can be determined using the following Stratton-Chu relations

$$\mathbf{E}(\mathbf{r}) = \oint_S \left[i\omega\mu(\hat{\mathbf{n}}\times\mathbf{H})G - (\hat{\mathbf{n}}\times\mathbf{E})\times\nabla G - (\hat{\mathbf{n}}\cdot\mathbf{E})\nabla G\right]dS \quad (5)$$

and

$$\mathbf{H}(\mathbf{r}) = -\oint_S \left[i\omega\varepsilon(\hat{\mathbf{n}}\times\mathbf{E})G + (\hat{\mathbf{n}}\times\mathbf{H})\times\nabla G + (\hat{\mathbf{n}}\cdot\mathbf{H})\nabla G\right]dS \quad (6)$$

where

$$G = \frac{1}{4\pi}\frac{e^{-ik|\mathbf{r}-\mathbf{r}'|}}{|\mathbf{r}-\mathbf{r}'|} \quad (7)$$

is Green's function and where $\mathbf{r}$ is the position vector of the observation point and $\mathbf{r}'$ is the position vector of the source point such that Green's function $G(\mathbf{r}-\mathbf{r}')$ is the kernel that propagates the contributions from each point $\mathbf{r}'$ on the bounding surface to the observation point $\mathbf{r}$ in the far field.

The Stratton-Chu relation integrates three Green's function terms from each discretized point of the bounding surface to each observation point in the far field. This compute-intensive process is GPU accelerated such that these contributions are computed in parallel.

Minimum feature constraints via differentiable neuroparameterization

The manufacturing-aware optimization combines continuous neural SDF representations with discrete geometric constraints through three core components. Surface normals are calculated using automatic differentiation of the neural SDF outputs, derived through gradient backpropagation from the prediction head to input coordinates

$$\mathbf{n}_i = \frac{\nabla_{\mathbf{p}_i}\mathcal{S}(\mathbf{p}_i)}{\|\nabla_{\mathbf{p}_i}\mathcal{S}(\mathbf{p}_i)\| + 10^{-8}} \quad (8)$$

where $\mathcal{S}$ represents the neural SDF prediction. These normals guide the stochastic sampling of verification points along both interior and exterior directions relative to structural boundaries, with offset lengths scaled by the minimum fabrication constraints.

The constraint enforcement alternates between boundary refinement and spatial consistency checks. The boundary identification first locates zero-crossing points through gradient-based optimization of the residual

$$\min_{\mathbf{p}} \|\mathcal{S}(\mathbf{p})\|^2 \ \text{(implemented through five Adam iterations)} \quad (9)$$

A hybrid point maintenance strategy preserves geometric features while enabling topological exploration. A total of 25% points come from low-residual grid locations (threshold $\tau = 10^{-5}$), 50% from previous optimized positions, and the remainder from stochastic sampling. Fixed boundary conditions at $x = \{0, 1\}$ ensure structural connectivity across the domain.

The neural SDF formulation enables differentiable feature size control through the gradient-driven sampling of the implicit boundary representation. For each boundary coordinate $\mathbf{p}_i$, we generate multiple verification samples $\mathbf{p}_i^{\pm} = \mathbf{p}_i \pm \delta_j\mathbf{n}_i$, where $\delta_j \in [0, \Delta_{\max}]$ follows a uniform distribution. By constraining the SDF values at strategically sampled offsets from the zero-level set (material interface), the loss function intrinsically enforces minimum feature sizes. For each boundary point $\mathbf{p}_i$ identified through zero-crossing optimization, the method samples verification locations $\mathbf{p}_i \pm \delta\mathbf{n}_i$ along surface normals $\mathbf{n}_i$ derived via automatic differentiation. The directional constraints $\mathcal{L}_{\text{feature}}$ penalize both:

1) Negative SDF values at $\mathbf{p}_i - \delta_{\text{gap}}\mathbf{n}_i$ to prevent adjacent features from closing below the minimum gap (δ_{gap}).

2) Positive SDF values at $\mathbf{p}_i + \delta_{\text{post}}\mathbf{n}_i$ to limit material protrusion widths to above δ_{post}.

For large-scale metasurface optimization, the framework processes K independent structures simultaneously through dimensionally parallelized operations. Shared encoder weights enable feature consistency across designs while maintaining model-specific parameters in the prediction head. Spatial gradients and loss terms are batch normalized across all parallel models to stabilize multitask optimization. The constraint weighting factor λ_{feature} ramps up linearly

Input: Target phase φ, wavelength λ, feature size constraint

Output: Optimized metasurface structure $\epsilon_T^\star$

for $g = 1$ **to** G **(parallel over** G **GPUs) do in parallel**

  Initialize a batch of devices $\{\epsilon_{N,1}^{(g,b)}\}_{b=1}^{B}$ on GPU g, where B is the batch size per GPU;

  for $b = 1$ **to** B **(parallel over batch elements within GPU** g**) do in parallel**

    for $t = 0$ **to** $T - 1$ **do**

      Solve $H_z^{(g,b)}$ near field using FiLM-Conditioned WaveY-Net with given source

      Compute $E_x^{(g,b)}$ and $E_y^{(g,b)}$ from $H_z^{(g,b)}$ using Ampere's Law

      Calculate far-field profile using Stratton-Chu near-to-far field transformation

      Calculate the FoM function for device (g, b)

      Backpropagate the FoM

      Update $\epsilon_{t+1}^{(g,b)}$ in Neuroshaper based on the gradient

    Track the best structure $\epsilon_T^{(g,b)}$ and corresponding FoM for each batch element

Select the final optimized structure:

$$\epsilon_T^\star = \mathrm{argmin}_{g,b}\ \mathrm{FoM}(\epsilon_T^{(g,b)})$$

Algorithm 1. Superpixel optimization process via end-to-end differentiation.

during early optimization phases to balance initial exploration with eventual manufacturability enforcement.

Metasurface optimization via parallel superpixel autodifferentiation

The entire optimization process can be decomposed into the following steps. Each step is implemented in PyTorch so that a gradient can be obtained for optimization using autodifferentiation of the whole computational graph end to end.

1) Neuroshaper updates the superpixel structure on the basis of the gradient from the last iteration, enforcing minimum feature constraints.

2) The H_z near field of the superpixel is solved by FiLM-Conditioned WaveY-Net with the given source. E_x and E_y are determined using Ampere's Law operating on a Yee grid

$$E_x^{i,k} = \frac{i}{\omega\varepsilon_x^{i,k}} \cdot \frac{H_z^{i,k} - H_z^{i,k-1}}{\Delta y} \tag{10}$$

and

$$E_y^{i,k} = \frac{-i}{\omega\varepsilon_y^{i,k}} \cdot \frac{H_z^{i,k} - H_z^{i-1,k}}{\Delta x} \tag{11}$$

where i and k are the discrete indices of the horizontal and vertical voxels in the Yee grid, respectively.

3) The far-field profile of the superpixel is calculated using the Stratton-Chu near-to-far field transformation.

4) The FoM is calculated using the formula from Fig. 4A (top). The desired phase of both metalenses and deflectors is a function of the superpixels' center location, x

$$\varphi_{\mathrm{lens}}(x, \lambda) = -\frac{2\pi}{\lambda}\sqrt{\left(x - x_{\mathrm{offset}}\right)^2 + f^2} \tag{12}$$

and

$$\varphi_{\mathrm{deflector}}(x, \lambda) = \frac{2\pi}{\lambda} x\left(\sin\theta_{\mathrm{target}} - \sin\theta_{\mathrm{in}}\right) \tag{13}$$

where f is the focal length, x_{offset} is the lateral offset amount of the focal point of the metalens, θ_{target} is the target deflection angle, and θ_{in} is the incident angle of the beam.

5) The gradient with respect to the input structure from step (1) is calculated by backpropagating the FoM throughout the entire computational graph.

The end-to-end optimization process is delineated in Algorithm 1.

Supplementary Materials

The PDF file includes:

Supplementary Text
Figs. S1 to S13
Tables S1 to S6
Legends for movies S1 and S2
Legends for data S1 to S5
References

Other Supplementary Material for this manuscript includes the following:

Movies S1 and S2
Data S1 to S5

REFERENCES AND NOTES

1. G. Kim, Y. Kim, J. Yun, S.-W. Moon, S. Kim, J. Kim, J. Park, T. Badloe, I. Kim, J. Rho, Metasurface-driven full-space structured light for three-dimensional imaging. *Nat. Commun.* **13**, 5920 (2022).
2. E. Tseng, S. Colburn, J. Whitehead, L. Huang, S.-H. Baek, A. Majumdar, F. Heide, Neural nano-optics for high-quality thin lens imaging. *Nat. Commun.* **12**, 6493 (2021).
3. M. Gopakumar, G.-Y. Lee, S. Choi, B. Chao, Y. Peng, J. Kim, G. Wetzstein, Full-colour 3D holographic augmented-reality displays with metasurface waveguides. *Nature* **629**, 791–797 (2024).
4. W.-J. Joo, J. Kyoung, M. Esfandyarpour, S.-H. Lee, H. Koo, S. Song, Y.-N. Kwon, S. H. Song, J. C. Bae, A. Jo, M.-J. Kwon, S. H. Han, S.-H. Kim, S. Hwang, M. L. Brongersma, Metasurface-driven OLED displays beyond 10,000 pixels per inch. *Science* **370**, 459–463 (2020).
5. I. Kim, W.-S. Kim, K. Kim, M. A. Ansari, M. Q. Mehmood, T. Badloe, Y. Kim, J. Gwak, H. Lee, Y.-K. Kim, J. Rho, Holographic metasurface gas sensors for instantaneous visual alarms. *Sci Adv.* **7**, eabe9943 (2021).
6. Q. Guo, Z. Shi, Y.-W. Huang, E. Alexander, C.-W. Qiu, F. Capasso, T. Zickler, Compact single-shot metalens depth sensors inspired by eyes of jumping spiders. *Proc. Natl. Acad. Sci. U.S.A.* **116**, 22959–22965 (2019).
7. N. Yu, F. Capasso, Flat optics with designer metasurfaces. *Nat. Mater.* **13**, 139–150 (2014).
8. W. T. Chen, A. Y. Zhu, F. Capasso, Flat optics with dispersion-engineered metasurfaces. *Nat. Rev. Mater.* **5**, 604–620 (2020).
9. X. Ni, A. V. Kildishev, V. M. Shalaev, Metasurface holograms for visible light. *Nat. Commun.* **4**, 2807 (2013).
10. X. Lin, Y. Rivenson, N. T. Yardimci, M. Veli, Y. Luo, M. Jarrahi, A. Ozcan, All-optical machine learning using diffractive deep neural networks. *Science* **361**, 1004–1008 (2018).
11. N. Mohammadi Estakhri, B. Edwards, N. Engheta, Inverse-designed metastructures that solve equations. *Science* **363**, 1333–1338 (2019).
12. J. Jiang, M. Chen, J. A. Fan, Deep neural networks for the evaluation and design of photonic devices. *Nat. Rev. Mater.* **6**, 679–700 (2021).
13. W. Ma, Z. Liu, Z. A. Kudyshev, A. Boltasseva, W. Cai, Y. Liu, Deep learning for the design of photonic structures. *Nat. Photon.* **15**, 77–90 (2021).
14. Z. Li, R. Pestourie, Z. Lin, S. G. Johnson, F. Capasso, Empowering metasurfaces with inverse design: Principles and applications. *ACS Photon.* **9**, 2178–2192 (2022).
15. Z. Kuang, O. D. Miller, Computational bounds to light-matter interactions via local conservation laws. *Phys. Rev. Lett.* **125**, 263607 (2020).
16. J. Jiang, J. A. Fan, Global optimization of dielectric metasurfaces using a physics-driven neural network. *Nano Lett.* **19**, 5366–5372 (2019).
17. J. Jiang, J. A. Fan, Simulator-based training of generative neural networks for the inverse design of metasurfaces. *Nanophotonics* **9**, 1059–1069 (2020).
18. Z. A. Kudyshev, A. V. Kildishev, V. M. Shalaev, A. Boltasseva, Machine learning-assisted global optimization of photonic devices. *Nanophotonics* **10**, 371–383 (2020).
19. G. Arya, W. F. Li, C. Roques-Carmes, M. Soljačić, S. G. Johnson, Z. Lin, End-to-end optimization of metasurfaces for imaging with compressed sensing. *ACS Photon.* **11**, 2077–2087 (2024).
20. R. E. Christiansen, O. Sigmund, Inverse design in photonics by topology optimization: Tutorial. *J. Opt. Soc. Am. B* **38**, 496 (2021).
21. G. Angeris, J. Vučković, S. Boyd, Heuristic methods and performance bounds for photonic design. *Opt. Express* **29**, 2827–2854 (2021).
22. J. R. Thompson, H. D. Nelson-Quillin, E. J. Coyle, J. P. Vernon, E. S. Harper, M. S. Mills, Particle swarm optimization of polymer-embedded broadband metasurface reflectors. *Opt. Express* **29**, 43421 (2021).
23. V. Egorov, M. Eitan, J. Scheuer, Genetically optimized all-dielectric metasurfaces. *Opt. Express* **25**, 2583–2593 (2017).
24. S. Jafar-Zanjani, S. Inampudi, H. Mosallaei, Adaptive genetic algorithm for optical metasurfaces design. *Sci. Rep.* **8**, 11040 (2018).
25. Z. Yu, H. Cui, X. Sun, Genetically optimized on-chip wideband ultracompact reflectors and Fabry-Perot cavities. *Photon. Res.* **5**, B15 (2017).
26. O. D. Miller, "Photonic design: From fundamental solar cell physics to computational inverse design," Ph.D. thesis, University of California, Berkeley (2012).
27. D. Sell, J. Yang, S. Doshay, R. Yang, J. A. Fan, Large-angle, multifunctional metagratings based on freeform multimode geometries. *Nano Lett.* **17**, 3752–3757 (2017).
28. R. Pestourie, C. Pérez-Arancibia, Z. Lin, W. Shin, F. Capasso, S. G. Johnson, Inverse design of large-area metasurfaces. *Opt. Express* **26**, 33732–33747 (2018).
29. A. Y. Piggott, J. Petykiewicz, L. Su, J. Vučković, Fabrication-constrained nanophotonic inverse design. *Sci. Rep.* **7**, 1786 (2017).
30. M. Mansouree, A. McClung, S. Samudrala, A. Arbabi, Large-scale parametrized metasurface design using adjoint optimization. *ACS Photon.* **8**, 455–463 (2021).
31. S. Colburn, A. Zhan, A. Majumdar, Metasurface optics for full-color computational imaging. *Sci. Adv.* **4**, eaar2114 (2018).
32. C. Yeung, B. Pham, R. Tsai, K. T. Fountaine, A. P. Raman, DeepAdjoint: An all-in-one photonic inverse design framework integrating data-driven machine learning with optimization algorithms. *ACS Photon.* **10**, 884–891 (2023).
33. R. Lupoiu, J. A. Fan, "Machine learning advances in computational electromagnetics" in *Advances in Electromagnetics Empowered by Artificial Intelligence and Deep Learning*, S. Campbell, D. Werner, Eds. (Wiley, ed. 1, 2023), pp. 225–252.
34. M. Raissi, P. Perdikaris, G. Karniadakis, Physics-informed neural networks: A deep learning framework for solving forward and inverse problems involving nonlinear partial differential equations. *J. Comput. Phys.* **378**, 686–707 (2019).
35. X. Meng, Z. Li, D. Zhang, G. E. Karniadakis, PPINN: Parareal physics-informed neural network for time-dependent PDEs. *Comput. Methods Appl. Mech. Eng.* **370**, 113250 (2020).
36. E. Kharazmi, Z. Zhang, G. E. Karniadakis, hp-VPINNs: Variational physics-informed neural networks with domain decomposition. *Comput. Methods Appl. Mech. Eng.* **374**, 113547 (2021).
37. L. Lu, R. Pestourie, W. Yao, Z. Wang, F. Verdugo, S. G. Johnson, Physics-informed neural networks with hard constraints for inverse design. *SIAM J. Sci. Comput.* **43**, B1105–B1132 (2021).
38. L. Yuan, Y.-Q. Ni, X.-Y. Deng, S. Hao, A-PINN: Auxiliary physics informed neural networks for forward and inverse problems of nonlinear integro-differential equations. *J. Comput. Phys.* **462**, 111260 (2022).
39. Z. Li, N. Kovachki, K. Azizzadenesheli, B. Liu, K. Bhattacharya, A. Stuart, A. Anandkumar, Fourier neural operator for parametric partial differential equations. arXiv:2010.08895 (2021).
40. N. Kovachki, S. Lanthaler, S. Mishra, On universal approximation and error bounds for Fourier neural operators. *J. Mach. Learn. Res.* **22**, 1–76 (2021).
41. G. Wen, Z. Li, K. Azizzadenesheli, A. Anandkumar, S. M. Benson, U-FNO—An enhanced Fourier neural operator-based deep-learning model for multiphase flow. *Adv. Water Resour.* **163**, 104180 (2022).
42. Z. Li, D. Z. Huang, B. Liu, Fourier neural operator with learned deformations for PDEs on general geometries. *J. Mach. Learn. Res.* **24**, 1–26 (2023).
43. M. Chen, R. Lupoiu, C. Mao, D.-H. Huang, J. Jiang, P. Lalanne, J. A. Fan, High speed simulation and freeform optimization of nanophotonic devices with physics-augmented deep learning. *ACS Photon.* **9**, 3110–3123 (2022).
44. C. Mao, R. Lupoiu, T. Dai, M. Chen, J. A. Fan, Towards general neural surrogate solvers with specialized neural accelerators. arXiv:2405.02351 (2024).
45. P. R. Wiecha, O. L. Muskens, Deep learning meets nanophotonics: A generalized accurate predictor for near fields and far fields of arbitrary 3D nanostructures. *Nano Lett.* **20**, 329–338 (2020).
46. M. Zhelyeznyakov, J. Fröch, A. Wirth-Singh, J. Noh, J. Rho, S. Brunton, A. Majumdar, Large area optimization of meta-lens via data-free machine learning. *Commun. Eng.* **2**, 60 (2023).
47. D. Lu, Y. Deng, J. M. Malof, W. J. Padilla, Learning electromagnetic metamaterial physics with ChatGPT. *IEEE Access* **13**, 51513–51526 (2025).
48. M. Kim, H. Park, J. Shin, Nanophotonic device design based on large language models: Multilayer and metasurface examples. *Nanophotonics* **14**, 1273–1282 (2025).
49. T. Ma, H. Wang, L. J. Guo, OptoGPT: A foundation model for inverse design in optical multilayer thin film structures. *Opto Electron. Adv.* **7**, 240062–240062 (2024).
50. A. Ghafarollahi, M. J. Buehler, AtomAgents: Alloy design and discovery through physics-aware multi-modal multi-agent artificial intelligence. arXiv:2407.10022 (2024).
51. X. Jiang, M. Zhang, Y. Song, Y. Zhang, Y. Wang, C. Ju, D. Wang, OptiComm-GPT: A GPT-based versatile research assistant for optical fiber communication systems. *Opt. Express* **32**, 20776–20796 (2024).
52. R. Li, C. Zhang, S. Mao, H. Huang, M. Zhong, Y. Cui, X. Zhou, F. Yin, S. Theodoridis, Z. Zhang, From English to PCSEL: LLM helps design and optimize photonic crystal surface emitting lasers. arXiv:2104.12145v2 (2023).
53. A. Ghafarollahi, M. J. Buehler, SciAgents: Automating scientific discovery through bioinspired multi-agent intelligent graph reasoning. *Adv. Mater.* **37**, 2413523 (2024).
54. A. Bandura, Social cognitive theory: An agentic perspective. *Annu. Rev. Psychol.* **52**, 1–26 (2001).
55. S. Yao, J. Zhao, D. Yu, N. Du, I. Shafran, K. Narasimhan, Y. Cao, ReAct: Synergizing reasoning and acting in language models. arXiv:2210.03629 (2023).
56. D. Fu, J. Huang, S. Lu, G. Dong, Y. Wang, K. He, W. Xu, PreAct: Prediction enhances agent's planning ability. arXiv:2402.11534 (2024).
57. K. Swanson, W. Wu, N. L. Bulaong, J. E. Pak, J. Zou, The virtual lab: AI agents design new SARS-CoV-2 nanobodies with experimental validation. bioRxiv 623004 [Preprint] (2024); https://doi.org/10.1101/2024.11.11.623004.
58. A. Vaswani, N. Shazeer, N. Parmar, J. Uszkoreit, L. Jones, A. N. Gomez, L. Kaiser, I. Polosukhin, Attention is all you need. arXiv:1706.03762 (2017).

59. T. B. Brown, B. Mann, N. Ryder, M. Subbiah, J. Kaplan, P. Dhariwal, A. Neelakantan, P. Shyam, G. Sastry, A. Askell, S. Agarwal, A. Herbert-Voss, G. Krueger, T. Henighan, R. Child, A. Ramesh, D. M. Ziegler, J. Wu, C. Winter, C. Hesse, M. Chen, E. Sigler, M. Litwin, S. Gray, B. Chess, J. Clark, C. Berner, S. McCandlish, A. Radford, I. Sutskever, D. Amodei, Language models are few-shot learners. arXiv:2005.14165 (2020).
60. L. McInnes, J. Healy, J. Melville, UMAP: Uniform Manifold Approximation and Projection for dimension reduction. arXiv:1802.03426 (2020).
61. J. Wei, X. Wang, D. Schuurmans, M. Bosma, B. Ichter, F. Xia, E. H. Chi, Q. V. Le, D. Zhou, Chain-of-thought prompting elicits reasoning in large language models. arXiv:2201.11903 (2022).
62. W.-L. Chiang, L. Zheng, Y. Sheng, A. N. Angelopoulos, T. Li, D. Li, B. Zhu, H. Zhang, M. I. Jordan, J. E. Gonzalez, I. Stoica, Chatbot Arena: An open platform for evaluating LLMs by human preference. arXiv:2403.04132v1 (2024).
63. L. Zheng, W.-L. Chiang, Y. Sheng, T. Li, S. Zhuang, Z. Wu, Y. Zhuang, Z. Li, Z. Lin, E. P. Xing, J. E. Gonzalez, I. Stoica, H. Zhang, LMSYS-CHAT-1M: A large-scale real-world LLM conversation dataset. arXiv:2309.11998 (2024).
64. T. Phan, D. Sell, E. W. Wang, S. Doshay, K. Edee, J. Yang, J. A. Fan, High-efficiency, large-area, topology-optimized metasurfaces. *Light Sci Appl* **8**, 48 (2019).
65. T. W. Hughes, I. A. D. Williamson, M. Minkov, S. Fan, Forward-mode differentiation of Maxwell's equations. *ACS Photon.* **6**, 3010–3016 (2019).
66. J. Lim, D. Psaltis, MaxwellNet: Physics-driven deep neural network training based on Maxwell's equations. *APL Photon.* **7**, 011301 (2022).
67. E. Perez, F. Strub, H. de Vries, V. Dumoulin, A. Courville, FiLM: Visual Reasoning with a general conditioning layer. arXiv:1709.07871 (2017).
68. D. P. Kingma, J. Ba, Adam: A Method for Stochastic Optimization. arXiv:1412.6980 (2015).
69. B. H. Chen, P. C. Wu, V.-C. Su, Y.-C. Lai, C. H. Chu, I. C. Lee, J.-W. Chen, Y. H. Chen, Y.-C. Lan, C.-H. Kuan, D. P. Tsai, GaN metalens for pixel-level full-color routing at visible light. *Nano Lett.* **17**, 6345–6352 (2017).
70. T. Choi, C. Choi, J. Bang, Y. Kim, H. Son, C. Kim, J. Jang, Y. Jeong, B. Lee, Multiwavelength achromatic deflector in the visible using a single-layer freeform metasurface. *Nano Lett.* **24**, 10980–10986 (2024).
71. H. W. Chung, L. Hou, S. Longpre, B. Zoph, Y. Tay, W. Fedus, Y. Li, X. Wang, M. Dehghani, S. Brahma, A. Webson, S. S. Gu, Z. Dai, M. Suzgun, X. Chen, A. Chowdhery, A. Castro-Ros, M. Pellat, K. Robinson, D. Valter, S. Narang, G. Mishra, A. Yu, V. Zhao, Y. Huang, A. Dai, H. Yu, S. Petrov, E. H. Chi, J. Dean, J. Devlin, A. Roberts, D. Zhou, Q. V. Le, J. Wei, Scaling instruction-finetuned language models. arXiv:2210.11416 (2022).
72. L. Ouyang, J. Wu, X. Jiang, D. Almeida, C. L. Wainwright, P. Mishkin, C. Zhang, S. Agarwal, K. Slama, A. Ray, J. Schulman, J. Hilton, F. Kelton, L. Miller, M. Simens, A. Askell, P. Welinder, P. Christiano, J. Leike, R. Lowe, Training language models to follow instructions with human feedback. arXiv:2203.02155 (2022).
73. H. Touvron, T. Lavril, G. Izacard, X. Martinet, M.-A. Lachaux, T. Lacroix, B. Rozière, N. Goyal, E. Hambro, F. Azhar, A. Rodriguez, A. Joulin, E. Grave, G. Lample, LLaMA: Open and efficient foundation language models. arXiv:2302.13971 (2023).
74. Z. Shao, P. Wang, Q. Zhu, R. Xu, J. Song, X. Bi, H. Zhang, M. Zhang, Y. K. Li, Y. Wu, D. Guo, DeepSeekMath: Pushing the limits of mathematical reasoning in open language models. arXiv:2402.03300 (2024).
75. DeepSeek-AI, D. Guo, D. Yang, H. Zhang, J. Song, R. Zhang, R. Xu, Q. Zhu, S. Ma, P. Wang, X. Bi, X. Zhang, X. Yu, Y. Wu, Z.F. Wu, Z. Gou, Z. Shao, Z. Li, Z. Gao, A. Liu, B. Xue, B. Wang, B. Wu, B. Feng, C. Lu, C. Zhao, C. Deng, C. Zhang, C. Ruan, D. Dai, D. Chen, D. Ji, E. Li, F. Lin, F. Dai, F. Luo, G. Hao, G. Chen, G. Li, H. Zhang, H. Bao, H. Xu, H. Wang, H. Ding, H. Xin, H. Gao, H. Qu, H. Li, J. Guo, J. Li, J. Wang, J. Chen, J. Yuan, J. Qiu, J. Li, J.L. Cai, J. Ni, J. Liang, J. Chen, K. Dong, K. Hu, K. Gao, K. Guan, K. Huang, K. Yu, L. Wang, L. Zhang, L. Zhao, L. Wang, L. Zhang, L. Xu, L. Xia, M. Zhang, M. Zhang, M. Tang, M. Li, M. Wang, M. Li, N. Tian, P. Huang, P. Zhang, Q. Wang, Q. Chen, Q. Du, R. Ge, R. Zhang, R. Pan, R. Wang, R.J. Chen, R.L. Jin, R. Chen, S. Lu, S. Zhou, S. Chen, S. Ye, S. Wang, S. Yu, S. Zhou, S. Pan, S.S. Li, S. Zhou, S. Wu, S. Ye, T. Yun, T. Pei, T. Sun, T. Wang, W. Zeng, W. Zhao, W. Liu, W. Liang, W. Gao, W. Yu, W. Zhang, W.L. Xiao, W. An, X. Liu, X. Wang, X. Chen, X. Nie, X. Cheng, X. Liu, X. Xie, X. Liu, X. Yang, X. Li, X. Su, X. Lin, X.Q. Li, X. Jin, X. Shen, X. Chen, X. Sun, X. Wang, X. Song, X. Zhou, X. Wang, X. Shan, Y.K. Li, Y.Q. Wang, Y.X. Wei, Y. Zhang, Y. Xu, Y. Li, Y. Zhao, Y. Sun, Y. Wang, Y. Yu, Y. Zhang, Y. Shi, Y. Xiong, Y. He, Y. Piao, Y. Wang, Y. Tan, Y. Ma, Y. Liu, Y. Guo, Y. Ou, Y. Wang, Y. Gong, Y. Zou, Y. He, Y. Xiong, Y. Luo, Y. You, Y. Liu, Y. Zhou, Y.X. Zhu, Y. Xu, Y. Huang, Y. Li, Y. Zheng, Y. Zhu, Y. Ma, Y. Tang, Y. Zha, Y. Yan, Z.Z. Ren, Z. Ren, Z. Sha, Z. Fu, Z. Xu, Z. Xie, Z. Zhang, Z. Hao, Z. Ma, Z. Yan, Z. Wu, Z. Gu, Z. Zhu, Z. Liu, Z. Li, Z. Xie, Z. Song, Z. Pan, Z. Huang, Z. Xu, Z. Zhang, Z. Zhang, DeepSeek-R1: Incentivizing reasoning capability in LLMs via reinforcement learning. arXiv:2501.12948 (2025).
76. S. Yin, C. Fu, S. Zhao, K. Li, X. Sun, T. Xu, E. Chen, A survey on multimodal large language models. *Natl. Sci. Rev.* **11**, nwae403 (2024).
77. OpenAI, J. Achiam, S. Adler, S. Agarwal, L. Ahmad, I. Akkaya, F. L. Aleman, D. Almeida, J. Altenschmidt, S. Altman, S. Anadkat, R. Avila, I. Babuschkin, S. Balaji, V. Balcom, P. Baltescu, H. Bao, M. Bavarian, J. Belgum, I. Bello, J. Berdine, G. Bernadett-Shapiro, C. Berner, L. Bogdonoff, O. Boiko, M. Boyd, A.-L. Brakman, G. Brockman, T. Brooks, M. Brundage, K. Button, T. Cai, R. Campbell, A. Cann, B. Carey, C. Carlson, R. Carmichael, B. Chan, C. Chang, F. Chantzis, D. Chen, S. Chen, R. Chen, J. Chen, M. Chen, B. Chess, C. Cho, C. Chu, H. W. Chung, D. Cummings, J. Currier, Y. Dai, C. Decareaux, T. Degry, N. Deutsch, D. Deville, A. Dhar, D. Dohan, S. Dowling, S. Dunning, A. Ecoffet, A. Eleti, T. Eloundou, D. Farhi, L. Fedus, N. Felix, S. P. Fishman, J. Forte, I. Fulford, L. Gao, E. Georges, C. Gibson, V. Goel, T. Gogineni, G. Goh, R. Gontijo-Lopes, J. Gordon, M. Grafstein, S. Gray, R. Greene, J. Gross, S. S. Gu, Y. Guo, C. Hallacy, J. Han, J. Harris, Y. He, M. Heaton, J. Heidecke, C. Hesse, A. Hickey, W. Hickey, P. Hoeschele, B. Houghton, K. Hsu, S. Hu, X. Hu, J. Huizinga, S. Jain, S. Jain, J. Jang, A. Jiang, R. Jiang, H. Jin, D. Jin, S. Jomoto, B. Jonn, H. Jun, T. Kaftan, Ł. Kaiser, A. Kamali, I. Kanitscheider, N. S. Keskar, T. Khan, L. Kilpatrick, J. W. Kim, C. Kim, Y. Kim, J. H. Kirchner, J. Kiros, M. Knight, D. Kokotajlo, Ł. Kondraciuk, A. Kondrich, A. Konstantinidis, K. Kosic, G. Krueger, V. Kuo, M. Lampe, I. Lan, T. Lee, J. Leike, J. Leung, D. Levy, C. M. Li, R. Lim, M. Lin, S. Lin, M. Litwin, T. Lopez, R. Lowe, P. Lue, A. Makanju, K. Malfacini, S. Manning, T. Markov, Y. Markovski, B. Martin, K. Mayer, A. Mayne, B. M. Grew, Scott Mayer Mc Kinney, C. M. Leavey, P. M. Millan, J. M. Neil, D. Medina, A. Mehta, J. Menick, L. Metz, A. Mishchenko, P. Mishkin, V. Monaco, E. Morikawa, D. Mossing, T. Mu, M. Murati, O. Murk, D. Mély, A. Nair, R. Nakano, R. Nayak, A. Neelakantan, R. Ngo, H. Noh, L. Ouyang, C. O'K, J. Pachocki, A. Paino, J. Palermo, A. Pantuliano, G. Parascandolo, J. Parish, E. Parparita, A. Passos, M. Pavlov, A. Peng, A. Perelman, Filipe de Avila Belbute Peres, M. Petrov, Henrique Ponde de Oliveira Pinto, M. Pokorny, M. Pokrass, V. H. Pong, T. Powell, A. Power, B. Power, E. Proehl, R. Puri, A. Radford, J. Rae, A. Ramesh, C. Raymond, F. Real, K. Rimbach, C. Ross, B. Rotsted, H. Roussez, N. Ryder, M. Saltarelli, T. Sanders, S. Santurkar, G. Sastry, H. Schmidt, D. Schnurr, J. Schulman, D. Selsam, K. Sheppard, T. Sherbakov, J. Shieh, S. Shoker, P. Shyam, S. Sidor, E. Sigler, M. Simens, J. Sitkin, K. Slama, I. Sohl, B. Sokolowsky, Y. Song, N. Staudacher, F. P. Such, N. Summers, I. Sutskever, J. Tang, N. Tezak, M. B. Thompson, P. Tillet, A. Tootoonchian, E. Tseng, P. Tuggle, N. Turley, J. Tworek, Juan Felipe Cerón Uribe, A. Vallone, A. Vijayvergiya, C. Voss, C. Wainwright, J. J. Wang, A. Wang, B. Wang, J. Ward, J. Wei, C. J. Weinmann, A. Welihinda, P. Welinder, J. Weng, L. Weng, M. Wiethoff, D. Willner, C. Winter, S. Wolrich, H. Wong, L. Workman, S. Wu, J. Wu, M. Wu, K. Xiao, T. Xu, S. Yoo, K. Yu, Q. Yuan, W. Zaremba, R. Zellers, C. Zhang, M. Zhang, S. Zhao, T. Zheng, J. Zhuang, W. Zhuk, B. Zoph, GPT-4 technical report. arXiv:2303.08774 (2024).
78. J. Jiang, R. Lupoiu, E. W. Wang, D. Sell, J. Paul Hugonin, P. Lalanne, J. A. Fan, MetaNet: A new paradigm for data sharing in photonics research. *Opt. Express* **28**, 13670–13681 (2020).
79. M. F. Schubert, invrs-gym: A toolkit for nanophotonic inverse design research. arXiv:2410.24132 (2024).
80. A. Srivastava, A. Rastogi, A. Rao, Abu Awal Md Shoeb, A. Abid, A. Fisch, A. R. Brown, A. Santoro, A. Gupta, A. Garriga-Alonso, A. Kluska, A. Lewkowycz, A. Agarwal, A. Power, A. Ray, A. Warstadt, A. W. Kocurek, A. Safaya, A. Tazarv, A. Xiang, A. Parrish, A. Nie, A. Hussain, A. Askell, A. Dsouza, A. Slone, A. Rahane, A. S. Iyer, A. Andreassen, A. Madotto, A. Santilli, A. Stuhlmüller, A. Dai, A. La, A. Lampinen, A. Zou, A. Jiang, A. Chen, A. Vuong, A. Gupta, A. Gottardi, A. Norelli, A. Venkatesh, A. Gholamidavoodi, A. Tabassum, A. Menezes, A. Kirubarajan, A. Mullokandov, A. Sabharwal, A. Herrick, A. Efrat, A. Erdem, A. Karakaş, B. Ryan Roberts, B. S. Loe, B. Zoph, B. Bojanowski, B. Özyurt, B. Hedayatnia, B. Neyshabur, B. Inden, B. Stein, B. Ekmekci, B. Y. Lin, B. Howald, B. Orinion, C. Diao, C. Dour, C. Stinson, C. Argueta, C. F. Ramírez, C. Singh, C. Rathkopf, C. Meng, C. Baral, C. Wu, C. Callison-Burch, C. Waites, C. Voigt, C. D. Manning, C. Potts, C. Ramirez, C. E. Rivera, C. Siro, C. Raffel, C. Ashcraft, C. Garbacea, D. Sileo, D. Garrette, D. Hendrycks, D. Kilman, D. Roth, D. Freeman, D. Khashabi, D. Levy, D. M. González, D. Perszyk, D. Hernandez, D. Chen, D. Ippolito, D. Gilboa, D. Dohan, D. Drakard, D. Jurgens, D. Datta, D. Ganguli, D. Emelin, D. Kleyko, D. Yuret, D. Chen, D. Tam, D. Hupkes, D. Misra, D. Buzan, D. C. Mollo, D. Yang, D.-H. Lee, D. Schrader, E. Shutova, E. D. Cubuk, E. Segal, E. Hagerman, E. Barnes, E. Donoway, E. Pavlick, E. Rodola, E. Lam, E. Chu, E. Tang, E. Erdem, E. Chang, E. A. Chi, E. Dyer, E. Jerzak, E. Kim, E. E. Manyasi, E. Zheltonozhskii, F. Xia, F. Siar, F. Martínez-Plumed, F. Happé, F. Chollet, F. Rong, G. Mishra, G. I. Winata, G. de Melo, G. Kruszewski, G. Parascandolo, G. Mariani, G. Wang, G. Jaimovitch-López, G. Betz, G. Gur-Ari, H. Galijasevic, H. Kim, H. Rashkin, H. Hajishirzi, H. Mehta, H. Bogar, H. Shevlin, H. Schütze, H. Yakura, H. Zhang, H. M. Wong, I. Ng, I. Noble, J. Jumelet, J. Geissinger, J. Kernion, J. Hilton, J. Lee, J. F. Fisac, J. B. Simon, J. Koppel, J. Zheng, J. Zou, J. Kocoń, J. Thompson, J. Wingfield, J. Kaplan, J. Radom, J. Sohl-Dickstein, J. Phang, J. Wei, J. Yosinski, J. Novikova, J. Bosscher, J. Marsh, J. Kim, J. Taal, J. Engel, J. Alabi, J. Xu, J. Song, J. Tang, J. Waweru, J. Burden, J. Miller, J. U. Balis, J. Batchelder, J. Berant, J. Frohberg, J. Rozen, J. Hernandez-Orallo, J. Boudeman, J. Guerr, J. Jones, J. B. Tenenbaum, J. S. Rule, J. Chua, K. Kanclerz, K. Livescu, K. Krauth, K. Gopalakrishnan, K. Ignatyeva, K. Markert, K. D. Dhole, K. Gimpel, K. Omondi, K. Mathewson, K. Chiafullo, K. Shkaruta, K. Shridhar, K. M. Donell, K. Richardson, L. Reynolds, L. Gao, L. Zhang, L. Dugan, L. Qin, L. Contreras-Ochando, L.-P. Morency, L. Moschella, L. Lam, L. Noble, L. Schmidt, L. He, L. O. Colón, L. Metz, L. K. Şenel, M. Bosma, M. Sap, Maartje ter Hoeve, M. Farooqi, M. Faruqui, M. Mazeika, M. Baturan, M. Marelli, M. Maru, Maria Jose Ramírez Quintana, M. Tolkiehn, M. Giulianelli, M. Lewis, M. Potthast, M. L. Leavitt, M. Hagen, M. Schubert, M. O. Baitemirova, M. Arnaud, M. M. Elrath, M. A. Yee, M. Cohen, M. Gu, M. Ivanitskiy, M. Starritt, M. Strube, M. Swędrowski, M. Bevilacqua, M. Yasunaga, M. Kale, M. Cain, M. Xu, M. Suzgun, M. Walker, M. Tiwari, M. Bansal, M. Aminnaseri, M. Geva, M. Gheini, Mukund Varma T, N. Peng, N. A. Chi, N. Lee, Neta Gur-Ari Krakover, N. Cameron, N. Roberts, N. Doiron, N. Martinez, N. Nangia,

N. Deckers, N. Muennighoff, N. S. Keskar, N. S. Iyer, N. Constant, N. Fiedel, N. Wen, O. Zhang, O. Agha, O. Elbaghdadi, O. Levy, O. Evans, Pablo Antonio Moreno Casares, P. Doshi, P. Fung, P. P. Liang, P. Vicol, P. Alipoormolabashi, P. Liao, P. Liang, P. Chang, P. Eckersley, P. M. Htut, P. Hwang, P. Miłkowski, P. Patil, P. Pezeshkpour, P. Oli, Q. Mei, Q. Lyu, Q. Chen, R. Banjade, R. E. Rudolph, R. Gabriel, R. Habacker, R. Risco, R. Millière, R. Garg, R. Barnes, R. A. Saurous, R. Arakawa, R. Raymaekers, R. Frank, R. Sikand, R. Novak, R. Sitelew, R. L. Bras, R. Liu, R. Jacobs, R. Zhang, R. Salakhutdinov, R. Chi, R. Lee, R. Stovall, R. Teehan, R. Yang, S. Singh, S. M. Mohammad, S. Anand, S. Dillavou, S. Shleifer, S. Wiseman, S. Gruetter, S. R. Bowman, S. S. Schoenholz, S. Han, S. Kwatra, S. A. Rous, S. Ghazarian, S. Ghosh, S. Casey, S. Bischoff, S. Gehrmann, S. Schuster, S. Sadeghi, S. Hamdan, S. Zhou, S. Srivastava, S. Shi, S. Singh, S. Asaadi, S. S. Gu, S. Pachchigar, S. Toshniwal, S. Upadhyay, S. Debnath, S. Shakeri, S. Thormeyer, S. Melzi, S. Reddy, S. P. Makini, S.-H. Lee, S. Torene, S. Hatwar, S. Dehaene, S. Divic, S. Ermon, S. Biderman, S. Lin, S. Prasad, S. T. Piantadosi, S. M. Shieber, S. Misherghi, S. Kiritchenko, S. Mishra, T. Linzen, T. Schuster, T. Li, T. Yu, T. Ali, T. Hashimoto, T.-L. Wu, T. Desbordes, T. Rothschild, T. Phan, T. Wang, T. Nkinyili, T. Schick, T. Kornev, T. Tunduny, T. Gerstenberg, T. Chang, T. Neeraj, T. Khot, T. Shultz, U. Shaham, V. Misra, V. Demberg, V. Nyamai, V. Raunak, V. Ramasesh, V. U. Prabhu, V. Padmakumar, V. Srikumar, W. Fedus, W. Saunders, W. Zhang, W. Vossen, X. Ren, X. Tong, X. Zhao, X. Wu, X. Shen, Y. Yaghoobzadeh, Y. Lakretz, Y. Song, Y. Bahri, Y. Choi, Y. Yang, Y. Hao, Y. Chen, Y. Belinkov, Y. Hou, Y. Hou, Y. Bai, Z. Seid, Z. Zhao, Z. Wang, Z. J. Wang, Z. Wang, Z. Wu, Beyond the imitation game: Quantifying and extrapolating the capabilities of language models. arXiv:2206.04615 (2023).

81. J. Gottweis, W.-H. Weng, A. Daryin, T. Tu, A. Palepu, P. Sirkovic, A. Myaskovsky, F. Weissenberger, K. Rong, R. Tanno, K. Saab, D. Popovici, J. Blum, F. Zhang, K. Chou, A. Hassidim, B. Gokturk, A. Vahdat, P. Kohli, Y. Matias, A. Carroll, K. Kulkarni, N. Tomasev, V. Dhillon, E. D. Vaishnav, B. Lee, T. R. D. Costa, J. R. Penadés, G. Peltz, Y. Xu, A. Pawlosky, A. Karthikesalingam, V. Natarajan, Towards an AI co-scientist. arXiv:2502.18864 (2025).
82. L. M. Roch, F. Häse, C. Kreisbeck, T. Tamayo-Mendoza, L. P. E. Yunker, J. E. Hein, A. Aspuru-Guzik, ChemOS: Orchestrating autonomous experimentation. *Sci. Robot.* **3**, eaat5559 (2018).
83. T. Dai, S. Vijayakrishnan, F. T. Szczypiński, J.-F. Ayme, E. Simaei, T. Fellowes, R. Clowes, L. Kotopanov, C. E. Shields, Z. Zhou, J. W. Ward, A. I. Cooper, Autonomous mobile robots for exploratory synthetic chemistry. *Nature* **635**, 890–897 (2024).
84. K. Hatakeyama-Sato, H. Ishikawa, S. Takaishi, Y. Igarashi, Y. Nabae, T. Hayakawa, Semiautomated experiment with a robotic system and data generation by foundation models for synthesis of polyamic acid particles. *Polymer J.* **56**, 977–986 (2024).
85. S. J. Woo, C. Huang, J. Savall, B. L. Conrad, J. Luo, M. J. Schnitzer, An autonomous robotic system for high-throughput phenotyping and behavioral studies of individual fruit flies. bioRxiv 607451 [Preprint] (2024); https://doi.org/10.1101/2024.08.21.607451.
86. M. Chen, J. Tworek, H. Jun, Q. Yuan, Henrique Ponde de Oliveira Pinto, J. Kaplan, H. Edwards, Y. Burda, N. Joseph, G. Brockman, A. Ray, R. Puri, G. Krueger, M. Petrov, H. Khlaaf, G. Sastry, P. Mishkin, B. Chan, S. Gray, N. Ryder, M. Pavlov, A. Power, L. Kaiser, M. Bavarian, C. Winter, P. Tillet, F. P. Such, D. Cummings, M. Plappert, F. Chantzis, E. Barnes, A. Herbert-Voss, W. H. Guss, A. Nichol, A. Paino, N. Tezak, J. Tang, I. Babuschkin, S. Balaji, S. Jain, W. Saunders, C. Hesse, A. N. Carr, J. Leike, J. Achiam, V. Misra, E. Morikawa, A. Radford, M. Knight, M. Brundage, M. Murati, K. Mayer, P. Welinder, B. M. Grew, D. Amodei, S. M. Candlish, I. Sutskever, W. Zaremba, Evaluating large language models trained on code. arXiv:2107.03374 (2021).
87. O. Ronneberger, P. Fischer, T. Brox, U-Net: Convolutional networks for biomedical image segmentation. arXiv:1505.04597 (2015).
88. S. Ioffe, C. Szegedy, Batch normalization: Accelerating deep network training by reducing internal covariate shift. arXiv:1502.03167 (2015).
89. A. L. Maas, A. Y. Hannun, A. Y. Ng, "Rectifier nonlinearities improve neural network acoustic models," in *Proceedings of the 30th International Conference on Machine Learning* (Proceedings of Machine Learning Research, 2013).
90. K. He, X. Zhang, S. Ren, J. Sun, Deep residual learning for image recognition. arXiv:1512.03385 (2015).
91. M. Chen, J. Jiang, J. A. Fan, Design space reparameterization enforces hard geometric constraints in inverse-designed nanophotonic devices. *ACS Photon.* **7**, 3141–3151 (2020).
92. K. Yee, Numerical solution of initial boundary value problems involving maxwell's equations in isotropic media. *IEEE Trans. Antennas Propag.* **14**, 302–307 (1966).
93. J. A. Stratton, L. J. Chu, Diffraction theory of electromagnetic waves. *Phys. Rev.* **56**, 99–107 (1939).
94. R. Lupoiu, Y. Shao, T. Dai, C. Mao, K. Edee, J. Fan, "Data and Code for MetaChat: A multi-agentic framework for real-time, autonomous freeform metasurface design" (2025); https://doi.org/10.5281/zenodo.15802727.
95. S. Kang, S. Uchida, B. K. Iwana, Tunable U-Net: Controlling image-to-image outputs using a tunable scalar value. *IEEE Access* **9**, 103279–103290 (2021).
96. V. Medvedev, A. Erdmann, A. Rosskopf, Physics-informed deep learning for 3D modeling of light diffraction from optical metasurfaces. *Opt. Express* **33**, 1371–1384 (2025).
97. M. N. Polyanskiy, Refractiveindex.info database of optical constants. *Sci. Data* **11**, 94 (2024).
98. S. A. Cholewiak, P. Ipeirotis, V. Silva, A. Kannawadi, "SCHOLARLY: Simple access to Google Scholar authors and citation using Python" (2021); https://github.com/scholarly-python-package/scholarly.
99. M. N. Polyanskiy, "Refractive index database" (2025); https://refractiveindex.info.
100. S. J. Reddi, S. Kale, S. Kumar, On the convergence of Adam and beyond. arXiv:1904.09237 (2019).

Acknowledgments: We thank M. Chen and Y. Zhou for technical assistance and helpful discussions. As outlined in the Materials and Methods, this study used and/or benchmarked the following LLMs: GPT-4o, GPT-4o Mini, Claude 3 Opus, Claude 3.5 Sonnet, Llama 3.1 405B, Llama 3.3 70B, and Llama 3.3 8B. Dall-E was used to generate the images of the agents and human designer in Figs. 1, 4, and 5. **Funding:** This work was supported by the National Aeronautics and Space Administration under award 80NSSC21K0220 and the National Science Foundation under award 2103301. R.L. is supported by a graduate fellowship award from Knight-Hennessy Scholars at Stanford University. C.M. is supported by the Stanford Graduate Fellowship. **Competing interests:** The authors declare that they have no competing interests. **Author contributions:** R.L. and J.A.F. conceptualized the study. R.L. coded and performed the analysis for MetaChat, AIM, FiLM WaveY-Net, and all related benchmarking. Y.S. and R.L. designed and coded the optimization APIs. T.D. and Y.S. coded and implemented the differentiable neuroparameterization framework. R.L., T.D., Y.S., and C.M. contributed to the evaluation benchmark. K.E. provided the mathematical formulation and initial code for the Stratton-Chu near-to-far field transformation. R.L. coded the GPU-accelerated version of Stratton-Chu. R.L. and J.A.F wrote the manuscript with input from all authors. J.A.F. supervised the research. **Data and materials availability:** All data needed to evaluate the conclusions in the paper are present in the paper and/or the Supplementary Materials. The latest code is available at https://github.com/jonfanlab/metachat and the latest benchmarks and data are available at http://metanet.stanford.edu/search/metachat (*78*). The code and data for this study have been deposited in Zenodo (https://doi.org/10.5281/zenodo.15802727) (*94*).

Submitted 28 March 2025
Accepted 29 September 2025
Published 31 October 2025
10.1126/sciadv.adx8006
