# Supplementary material for: A multi-agentic framework for real-time, autonomous freeform metasurface design
Source: Sci Adv. 2025 Oct 31;11(44):eadx8006. doi: 10.1126/sciadv.adx8006 (PMC12577674; doi:10.1126/sciadv.adx8006)
Supplement: Supplementary file 5 — Erratum published 6 February 2026: References throughout the main text and Supplementary Materials have been renumbered. The Supplementary Materials PDF has been updated. The original version is available here: [file sciadv.adx8006_sm.v1.pdf]

Science Advances
AAAS

Supplementary Materials for

A multi-agentic framework for real-time, autonomous freeform metasurface design

Robert Lupoiu *et al.*

Corresponding author: Jonathan A. Fan, jonfan@stanford.edu

Sci. Adv. **11**, eadx8006 (2025)
DOI: 10.1126/sciadv.adx8006

The PDF file includes:

Supplementary Text
Figs. S1 to S13
Tables S1 to S6
Legends for movies S1 and S2
Legends for data S1 to S5
References

Other Supplementary Material for this manuscript includes the following:

Movies S1 and S2
Data S1 to S5

Supplementary Text

FiLM WaveY-Net benchmark against MLP conditioning

Conditioning methods for modulating U-Net architecture outputs using a multi-layer perceptron (MLP)-encoded representation of conditioning parameters have been previously explored (*95*). This approach has been successfully applied to condition the simulation of periodic TiO_2 meta-atoms with variable source angle and wavelength for a near-infrared bandwidth from 700nm to 800nm (*96*). This approach is benchmarked against FiLM WaveY-Net in Fig. S1. In our implementation, wavelength and angle parameters are normalized and fed into a three-layer MLP with hidden dimensions of 64 and 128, producing a 4×4 spatial feature map. This representation is concatenated with the bottleneck features of the U-Net, forming an additional channel that propagates through the decoder path, directly modulating the network's output based on the input parameters. Within the visible range of 400nm to 700nm considered in this study, with variable parameters as listed in Fig. 3C of the main text, the method of conditioning by injecting an MLP-encoded representation of parameters into the convolutional spine of the U-Net architecture fails to generalize to the test set.

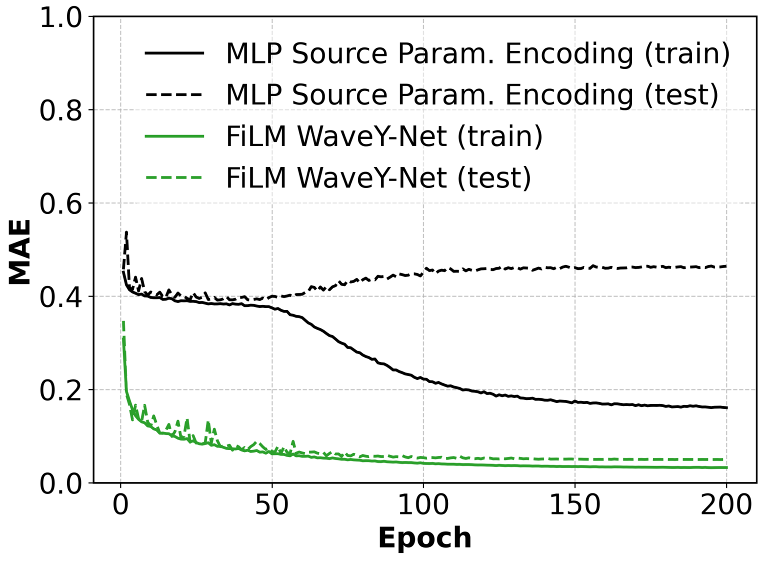

Figure S1: Benchmarking MLP source parameter encoding against FiLM WaveY-Net. Train (solid) and test (dashed) MAE is plotted over 200 epochs for MLP source parameter encoding conditioning (black) and FiLM WaveY-Net (green). The MLP source parameter encoding approach overfits on the data, whereas FiLM WaveY-Net generalizes to the test set.

Materials database design

The AIM Materials Expert Agent interacts with a curated database, with data pulled from `RefractiveIndex.info` (*97*). A relational database is necessary for the agent to interact with, instead of for example a simple lookup table, because the literature oftentimes contains multiple measurements of the same data under similar or even identical conditions. Choosing the correct data based on the context is a non-trivial problem that requires careful consideration of all the available information, including simulation problem specification, data collection metadata, data recency, and citation count as a proxy for data authoritativeness.

The schema for the relational database is illustrated in Fig. S2. The *materials* table contains high-level information about the data entry, including material formula name, wavelength range, and the data type (tabulated or dispersion formula). A primary key (PK) uniquely identifies each row in the *materials* database, which links to supporting tables via foreign keys (FKs). The *specs* table includes metadata information about the material measurements, such as the thickness, substrate, and temperature. This metadata is stored in an inconsistent text format in the source, which we extract using an instance of GPT-4o Mini that is prompted to identify this information and organize it in structured JSON. *material_references* contains information about the data source publication. GPT-4o Mini is again leveraged to extract the publication year, title, and journal information. Given that DOI links follow a prescribed pattern, a regular expression (regex) pattern is used to identify and extract them. Citation counts are extracted using a Google Scholar scraping tool (*98*). Given that studies have a variable number of authors, and individuals may contribute to more than one study, this is organized using a *reference_authors* table for each study that links to unique individuals stored in the *authors* table. Finally, the refractive index data is stored in *measurements* if the *materials* data type is "tabulated" or in *material_formulas* if it is "formula".

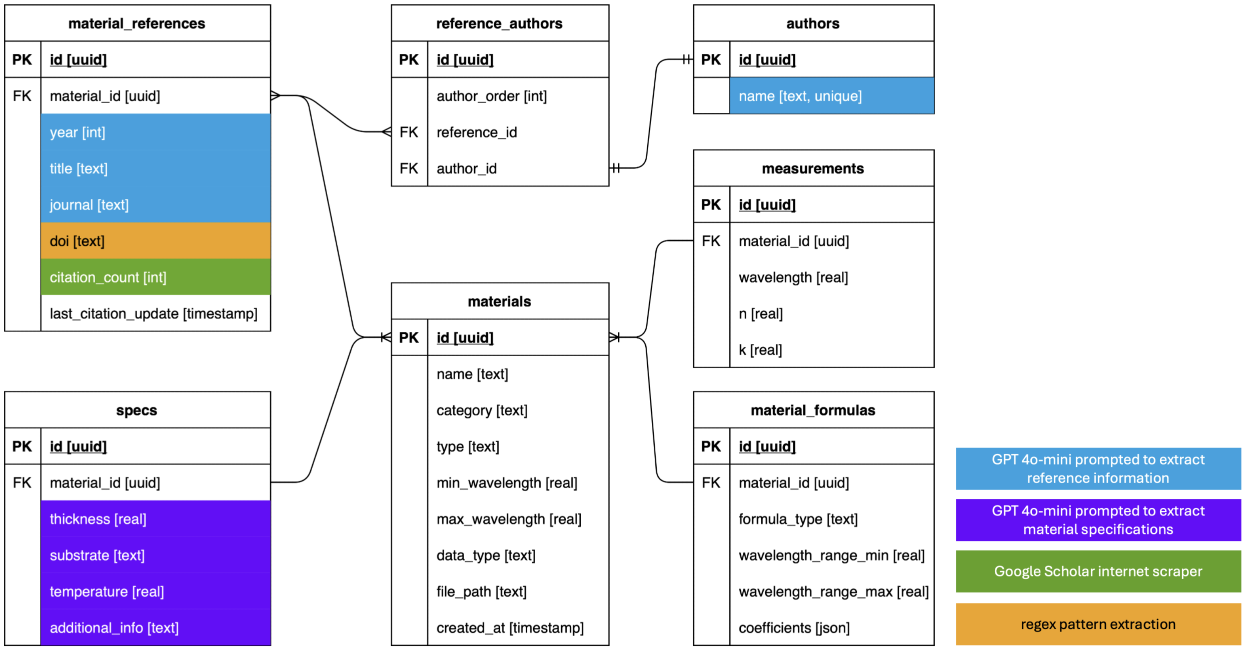

Figure S2: Materials Expert Agent database schema. Entity-relationship diagram for the materials database that the Materials Expert Agent interfaces with. FK relationships define how records are linked across tables. Color-coding is used to indicate the source of information extraction for the highlighted table entries. GPT-4o Mini extracts reference information (blue) and material metadata specifications (purple). Source publication citation counts are pulled from Google Scholar (purple). DOI extraction is implemented using regex patterns (orange).

Refractive index dispersion formulas

Data stored in the Materials Expert Agent database as dispersion formula coefficients is sourced from a variety of publications that use different dispersion formulas. Furthermore, different material types have different formula type conventions, such as gases and optical glasses. To account for this in a modular and flexible manner, the agent is prompted to interface with an API that calculates the refractive index at a given wavelength if specified the formula type, wavelength, and formula coefficients, as follows:

```
When you need to calculate a refractive index using a dispersion formula:
   <calculate_n>
   {{"formula_type": X, "wavelength": wavelength_in_microns, "coefficients": [c1, c2, ...]}}
   </calculate_n>
   The system will return the calculated refractive index value.
```

The API implements all of the dispersion formulas listed in *refractiveindex.info* (*99*):

$$n(\lambda) = \sqrt{1 + C_1 + \sum_{i=1}^{8} \frac{C_{2i}\,\lambda^2}{\lambda^2 - \left(C_{2i+1}\right)^2}}\,, \tag{S1}$$

$$n(\lambda) = \sqrt{1 + C_1 + \sum_{i=1}^{8} \frac{C_{2i}\,\lambda^2}{\lambda^2 - C_{2i+1}}}\,, \tag{S2}$$

$$n(\lambda) = \sqrt{C_1 + \sum_{i=1}^{8} C_{2i}\,\lambda^{C_{2i+1}}}\,, \tag{S3}$$

$$n(\lambda) = \sqrt{C_1 + \frac{C_2\,\lambda^{C_3}}{\lambda^2 - (C_4)^{C_5}} + \frac{C_6\,\lambda^{C_7}}{\lambda^2 - (C_8)^{C_9}} + \sum_{k=0}^{3} C_{10+2k}\,\lambda^{C_{11+2k}}}\,, \tag{S4}$$

$$n(\lambda) = C_1 + \sum_{i=1}^{5} C_{2i}\,\lambda^{C_{2i+1}}, \tag{S5}$$

$$n(\lambda) = 1 + C_1 + \sum_{i=1}^{5} \frac{C_{2i}}{C_{2i+1} - \lambda^{-2}}, \tag{S6}$$

$$n(\lambda) = C_1 + C_2\,\frac{1}{\lambda^2 - 0.028} + C_3\left(\frac{1}{\lambda^2 - 0.028}\right)^2 + C_4\,\lambda^2 + C_5\,\lambda^4 + C_6\,\lambda^6, \tag{S7}$$

$$x = C_1 + \frac{C_2\,\lambda^2}{\lambda^2 - C_3} + C_4\,\lambda^2, \tag{S8}$$

$$n(\lambda) = \sqrt{\frac{1 + 2x}{1 - x}}\,, \tag{S9}$$

$$n(\lambda) = \sqrt{C_1 + \frac{C_2}{\lambda^2 - C_3} + \frac{C_4\,(\lambda - C_5)}{(\lambda - C_5)^2 + C_6}}\,, \tag{S10}$$

where Eq. S1 is the Sellmeier formula, Eq. S2 is the Sellmeier-2 variant, Eq. S3 is the generalized polynomial formulation, Eq. S4 is the form used by `RefractiveIndex.INFO`, Eq. S5 is the Cauchy model, Eq. S6 is used for modeling gases, Eq. S7 is the Herzberger equation, Eqs. S8–S9 correspond

to the retro model, and Eq. S10 is a custom formulation.

SDF parameterized end-to-end optimization convergence and robustness analysis

Here, we formally treat the end-to-end SDF-parameterized optimization problem to demonstrate the convergence criteria and the rate to stationarity, as well as motivate the Monte-Carlo ensemble of initializations approach.

Problem Setting. Let $\boldsymbol{\theta} \in \mathbb{R}^d$ denote the parameters of the implicit neural signed–distance function (SDF) $S(\mathbf{p}; \boldsymbol{\theta})$ defining a single super–pixel. The end–to–end loss

$$\mathcal{L}(\boldsymbol{\theta}) \; = \; -\mathrm{FoM}\big(\boldsymbol{\theta}\big) \; + \; \lambda_{\text{feature}} \, \mathcal{L}_{\text{pen}}\big(\boldsymbol{\theta}\big), \tag{S11}$$

is the sum of (i) the negative figure of merit (FoM) returned by the differentiable pipeline

$$S \xrightarrow{\text{voxelisation}} \varepsilon_r \xrightarrow{\text{FiLM WaveY-Net}} \mathbf{H}_z \xrightarrow{\text{Stratton–Chu}} \tilde{\mathbf{E}}(\mathbf{k}) \xrightarrow{\text{post-processing}} \mathrm{FoM},$$

and (ii) the soft geometric-constraint penalty $\mathcal{L}_{\text{pen}}$ At iteration t, Adam (AMSGrad variant) updates

$$\mathbf{m}_t = \beta_1 \mathbf{m}_{t-1} + (1-\beta_1) \nabla \mathcal{L}(\boldsymbol{\theta}_{t-1}), \tag{S12}$$

$$\mathbf{v}_t = \max\big\{\mathbf{v}_{t-1}, \, \beta_2 \mathbf{v}_{t-1} + (1-\beta_2) \nabla \mathcal{L}(\boldsymbol{\theta}_{t-1})^2\big\}, \tag{S13}$$

$$\boldsymbol{\theta}_t = \boldsymbol{\theta}_{t-1} - \alpha_t \frac{\mathbf{m}_t}{\sqrt{\mathbf{v}_t} + \varepsilon}, \tag{S14}$$

where $\alpha_t = \alpha_0 / \sqrt{t}$ and $\beta_1 < \sqrt{\beta_2} < 1$.

Guarantee. Assume that

1. $\mathcal{L}$ is bounded below, and
2. $\nabla \mathcal{L}$ is L–Lipschitz ($\|\nabla \mathcal{L}(\boldsymbol{\theta}) - \nabla \mathcal{L}(\boldsymbol{\theta}')\| \leq L \|\boldsymbol{\theta} - \boldsymbol{\theta}'\|$).

With AMSGrad and the steps above,

$$\min_{1 \leq t \leq T} \mathbb{E}\big[\|\nabla \mathcal{L}(\boldsymbol{\theta}_t)\|^2\big] \; \leq \; \frac{C}{\sqrt{T}}, \tag{S15}$$

for some constant C depending on L, α_0, and the bounded second moments of the stochastic gradients (*100*). Thus the algorithm converges in expectation to a *first–order stationary point* of (S11).

Role of the Adaptive Denominator. The per-coordinate scaling by $\sqrt{\mathbf{v}_t}$ mitigates the severe anisotropy that arises from: (i) very steep barrier directions in $\mathcal{L}_{\text{pen}}$ once $\lambda_{\text{feature}} \to \lambda_{\max}$, and (ii) the disparate sensitivities of the chain-ruled field quantities. Without the "max" correction of AMSGrad, vanishing denominators may yield unbounded steps and divergence on non-convex surfaces.

(i) Neural SDF Smoothness. The MLP implementing $S(\mathbf{p};\boldsymbol{\theta})$ is required to be C^1 in both $\mathbf{p}$ and $\boldsymbol{\theta}$, with bounded partials:

$$\sup_{\mathbf{p},\boldsymbol{\theta}} \{ \|\nabla_{\mathbf{p}} S\|, \ \|\nabla_{\boldsymbol{\theta}} S\| \ < \infty. \tag{S16}$$

Weight-clipping or spectral-norm regularization suffices in practice.

(ii) Surrogate-Model Regularity. If every FiLM block employs Lipschitz activations (e.g. SiLU) and the weights are bounded, the surrogate mapping $\varepsilon_r \mapsto \mathbf{H}_z$ is $L_{\mathcal{N}}$–Lipschitz, which propagates the gradient-Lipschitz property through the pipeline.

(iii) Far-Field and FoM Differentiability. The Stratton–Chu surface integral is linear and smooth; the FoM is typically quadratic in electric-field magnitudes at a *finite* set of (θ, ϕ) angles, hence C^∞.

(iv) Penalty Ramp. Let $\lambda_{\text{feature}}(t)$ grow from 0 to $\lambda_{\max}$ with $\dot{\lambda}_{\text{feature}} \le \gamma$ (γ small). This avoids discontinuities that would violate assumption (A2).

Under (i)–(iv) the composite loss remains L–smooth and the guarantee (S15) applies.

Penalty Construction. For each zero–level boundary point $\mathbf{p}_i$ with outward normal $\mathbf{n}_i$,

$$\mathcal{L}_{\text{pen}} = \sum_{i,j} \Big[\underbrace{\max\big(0, -S(\mathbf{p}_i - \delta_{\text{gap}}\, \mathbf{n}_i)\big)}_{\text{gap constraint}} + \underbrace{\max\big(0,\ S(\mathbf{p}_i + \delta_{\text{post}}\, \mathbf{n}_i)\big)}_{\text{post-width constraint}} \Big]. \tag{S17}$$

Landscape Modification. The smooth barriers produced by the ReLU terms

$$\max(0, \pm S(\cdot)) \tag{S18}$$

- create new local minima where the FoM is sub-optimal but constraints are perfectly satisfied;
- flatten existing minima near feasibility boundaries, which may slow final convergence yet improves tolerance to fabrication perturbations;
- remove non-differentiable jumps present in projection methods, yielding more stable training trajectories.

Empirically, the linear ramp of λ_{feature} maintains fast early progress and postpones barrier steepness until the optimizer is already close to a high-FoM basin.

FiLM WaveY-Net domain region error contribution analysis

FiLM WaveY-Net features extremely high accuracy rates over a broad range of geometries, material properties, and sources, as outlined in the main text. As a surrogate solver, however, its solutions are not perfectly identical to those produced by a ground truth solver. Although the level of accuracy demonstrated by FiLM WaveY-Net is sufficient for producing ground-truth validated optimized structures, in this section we analyze the regions prone to the highest error rates, which can be the target for further accuracy improvement in future work.

High-intensity regions of resonance are one often-purported source of disproportional surrogate solver error. To probe the validity of this hypothesis for FiLM WaveY-Net, we devise an automated method for generating masks that are used to extract error values only in the high-intensity regions of interest. To achieve this, the contrast of the field map is first enhanced. This is achieved by denoising using a Gaussian filter convolution ($\sigma = 1.0$ pix.) for smoothing, followed by a second, broader Gaussian filter ($\sigma = 10.0$ pix.) to estimate the local background. The background is subtracted from the smoothed field map to produce a contrast map. The pixels in the 99th percentile of contrast are then selected, with an adaptive threshold relaxation applied if no pixels are found. Finally, small artifacts (smaller than 4 pixels) are removed to produce the hot-spot masks (examples plotted in Fig. S3A-D). To evaluate the relative contribution of the fields in the hot-spot regions compared

to the rest of the field map, we calculate the MAE in the extracted regions and divide them by the overall MAE of the whole field map. The distribution is plotted in Fig. S3E, which has an MAE of $\mu = 0.0292$, with a region over global mean ratio of 0.847. This indicates that on average, the hot-spot regions actually under-contribute to the overall observed error compared to other regions in the field map.

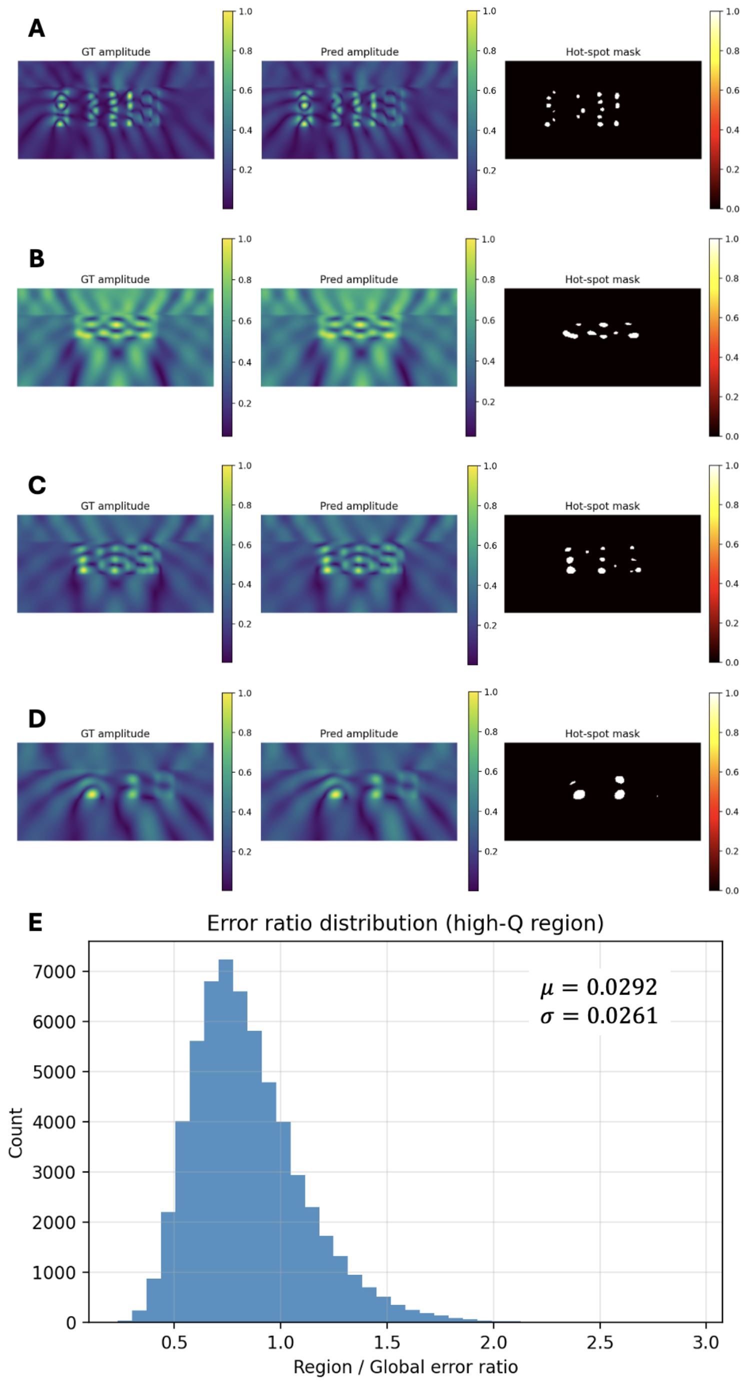

Figure S3: Hotspot region contribution analysis. (A)-(D) Randomly-sampled unseen test data performance, showing the ground truth H_z amplitude (left), the FiLM WaveY-Net predicted field (center), and the high-amplitude hot spot regions extraction mask (right). **(E)** Histogram counting the regional error in the hot-spot regions divided by the overall error of each example in turn. The mean hotspot-localized absolute error is 0.0292, with a standard deviation of 0.0261.

In the original WaveY-Net study, it was revealed that boundary regions are the source of the highest levels of noise, or error, in the field map (*43*). This was due to the underlying UNet

convolutional neural network architecture, which faces larger difficulty at boundaries in pixel-to-pixel regression tasks due to less accurate gradients in these regions of sharp input map transitions. To verify this phenomenon for FiLM WaveY-Net, we repeat the hot-spot region error analysis for the boundary regions of field maps. To produce the boundary masks, we first binarize the grayscale structures, with a dielectric threshold of 1.0. For each pixel, the absolute difference with its four-connected neighboring pixels is computed, and the pixel is marked as a boundary if the difference exceeds an edge tolerance of 0.001. Finally, the intermediate 1-pixel-wide boundary is dilated using a square structuring element that is 3 pixels wide (examples plotted in Figs. S4A-D, center-left). The resulting mask is a boolean array marking pixels in the regions near sharp boundaries after binarization. Again, the relative contribution to the overall error of the surrogate output, we divide the boundary region error by the global error for each sample in the test set. The distribution is plotted in Fig. S4E, which has an MAE of $\mu = 0.0410$, with a region over global mean ratio of 1.140. This indicates that on average, the boundary regions indeed over-contribute to the overall observed error compared to the rest of the field map, which is consistent with the findings from the original WaveY-Net study (*43*). This quantitative observation indicates that future work, potentially using a combination of iterative methods and attention mechanisms, can improve FiLM WaveY-Net performance by focusing primarily on the loss in the boundary regions of the outputted field map.

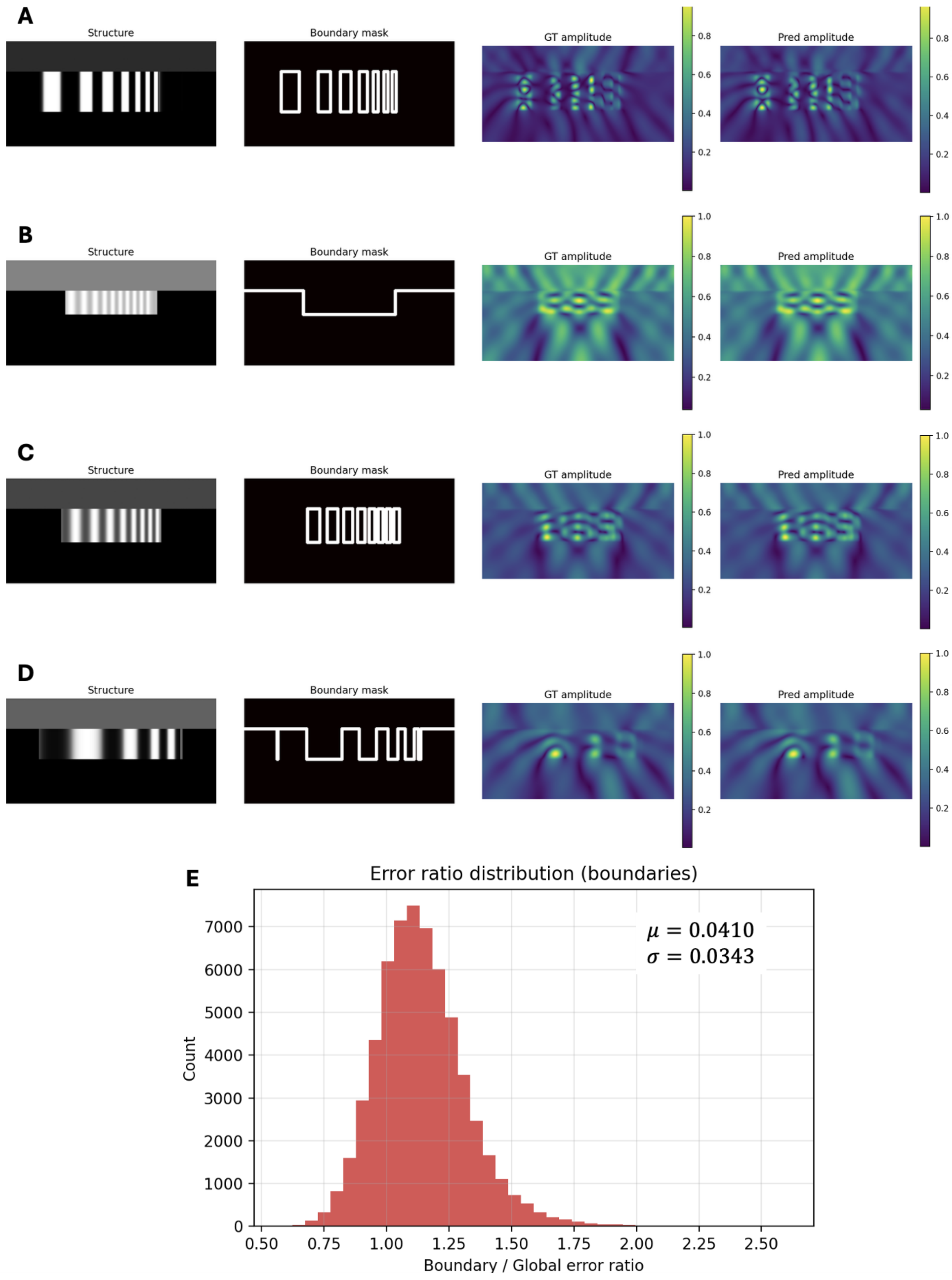

Figure S4: Boundary region contribution analysis. **(A)-(D)** Randomly-sampled unseen test data performance, showing the sampled structure (left-most), the extracted boundary mask (center-left), ground truth H_z amplitude (center-right), and the FiLM WaveY-Net predicted field (right-most). **(E)** Histogram counting the regional error in the boundary regions divided by the overall error of each example in turn. The mean boundary-localized absolute error is 0.0410, with a standard deviation of 0.0343.

FoM landscape analysis

In this section, we discuss the landscape of the FoM function and how we select the sample size during the optimization process of superpixels based on the convergence test result. For this analysis, we select a typical superpixel optimization problem, where the aim is to design a superpixel to deflect normally incident light to 25 degrees at a single wavelength of 650 nm. We optimized 3000 devices using the Neuroshaper representation and optimization method discussed in the main text.

We first optimized 3000 randomly initialized structures and collected their final optimized FoM value, which is defined in Fig. 4A of the main text. We show the histogram of the optimized FoM value in Fig. S5. The best FoM we can get for this problem is around -400. 242 out of 3000 trials produce a FoM below -300, and 22 out of 3000 trials produce a FoM below -360. Using these empirically-collected metrics, we can thus estimate the number of trials we need in order to achieve a certain performance requirement. As shown in the Fig. 4B in the main text, we plot the estimated best FoM value we expect to obtain given a certain number of trials using a Monte Carlo sampling of this FoM histogram distribution.

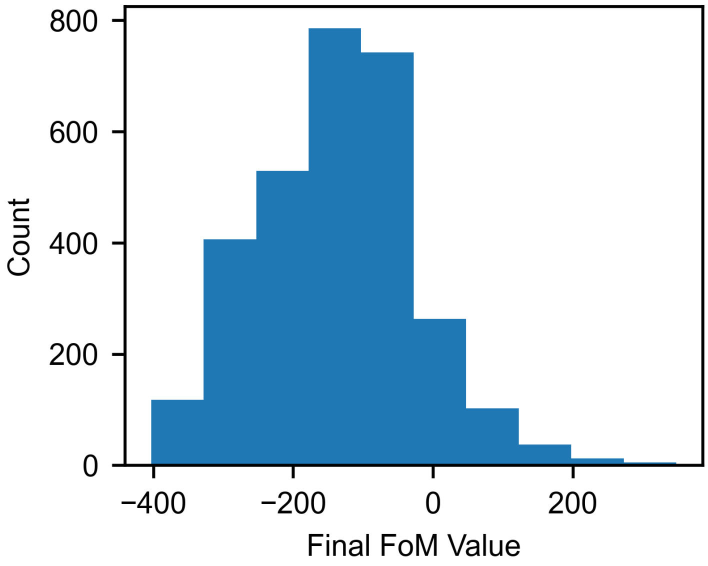

Figure S5: FoM Landscape Analysis. Histogram of the optimized FoM function value from a typical superpixel optimization problem.

FiLM WaveY-Net metalens design validation using a rigorous FDFD solver

In the main text, FiLM WaveY-Net is leveraged to perform electromagnetic simulations for acceleration that enables near real-time optimization. In this section, we compare the simulation results between FiLM WaveY-Net and a conventional FDFD solver, Ceviche, to evaluate the accuracy and efficiency of the surrogate solver for optimization. As a benchmark, we select a dual-wavelength, dual-objective metalens using the optimization method described in the main text. It contains a rigorous fullwave-manageable 20 superpixels, focuses 680 nm wavelength light to the center, and focuses 480 nm wavelength light offset to the right by 20 µm. The focal length at both wavelengths is 50 µm. In Figs. S6 and S7, we show the H_z field calculated by both FiLM WaveY-Net and Ceviche at both wavelengths, together with the optimized structure's dielectric constant profile. We can see that the fields predicted by FiLM WaveY-Net accurately match the ground-truth simulation results from the conventional FDFD solver Ceviche. This demonstrates the accuracy of the WaveY-Net-based simulation and optimization method across each of the individual superpixels that constitute an entire metalens.

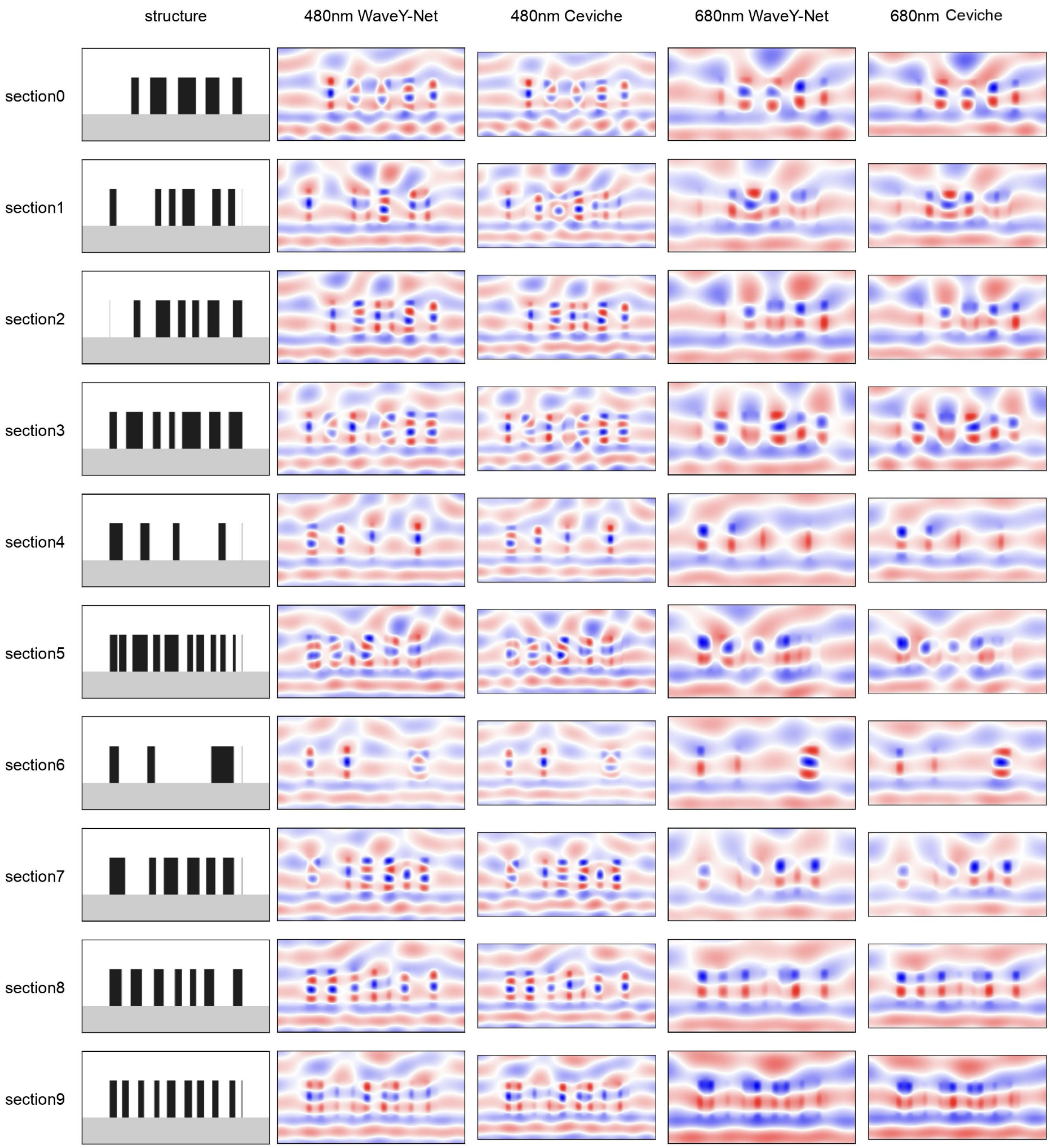

Figure S6: FiLM WaveY-Net benchmark against rigorous FDFD, metalens superpixels 0 through 9. Comparison of the H_z field calculated by FiLM WaveY-Net and Ceviche FDFD, at wavelengths of 480 nm and 680 nm, for each of the first ten superpixel sections that make up a dual-wavelength, dual-objective metalens optimized using MetaChat's modular superpixel optimization method.

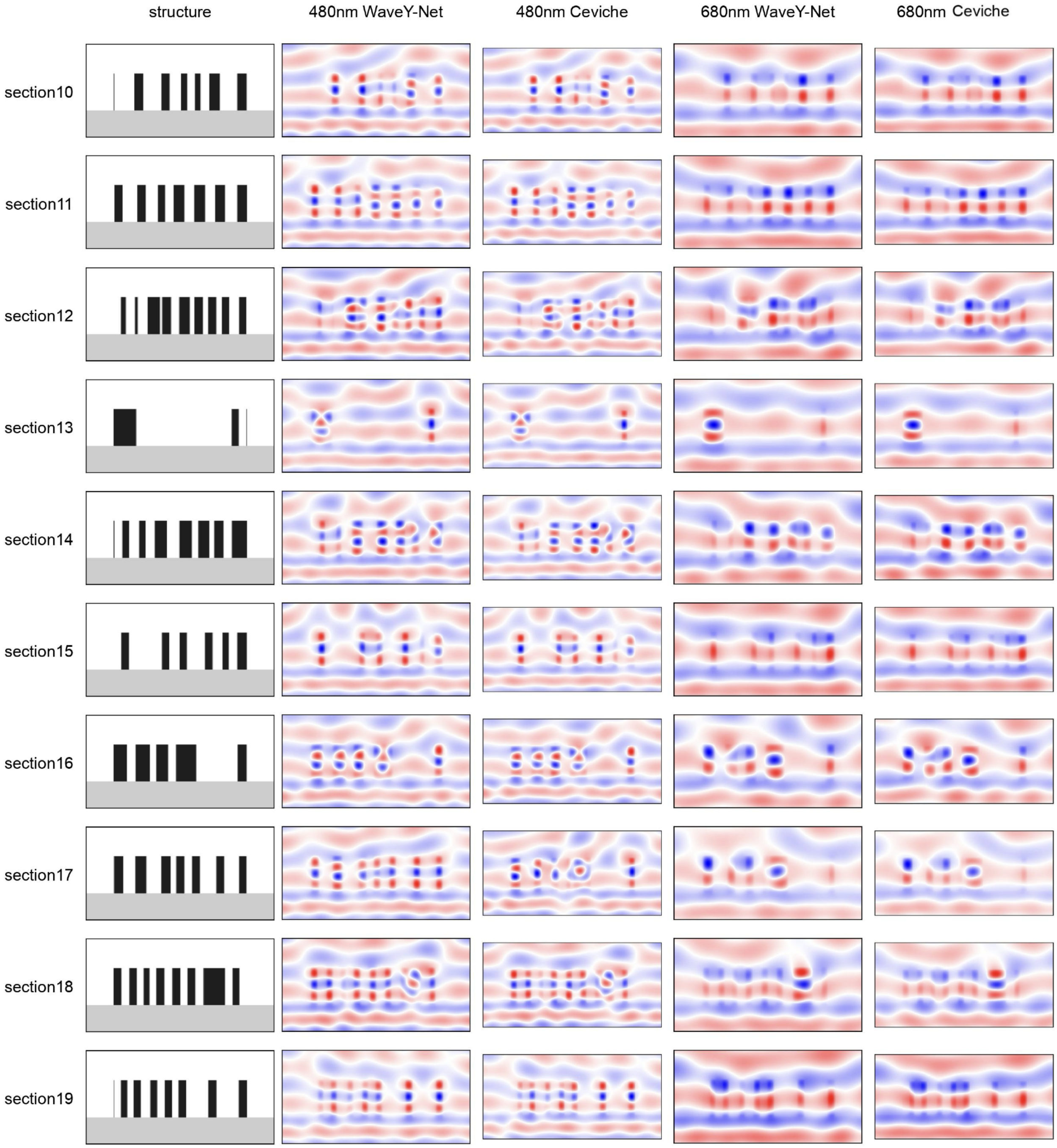

Figure S7: FiLM WaveY-Net benchmark against rigorous FDFD, metalens superpixels 10 through 19. Comparison of the H_z field calculated by FiLM WaveY-Net and Ceviche FDFD, at wavelengths of 480 nm and 680 nm, for each of the last ten superpixel sections that make up a dual-wavelength, dual-objective metalens optimized using MetaChat's modular superpixel optimization method.

We also perform a large-area Ceviche simulation on the whole metalens with 20 superpixels stitched together. The metalens structure's dielectric constant profile, and the simulated H_z at 480 nm and 680 nm wavelengths are shown in Figs. S8A-C. By comparing with the fields shown in

Figs. S6 and S7, we see that the whole field calculated from the stitched device matches well with each superpixel's small-size simulation result. This empirically indicates that by stitching the fields from each superpixel's simulation result, we can accurately approximate the whole field as long as there is enough separation air gap between neighboring superpixels. Therefore, we can optimize each superpixel individually and stitch them together subsequently. In addition, Fig. S8D shows the far field power map of the whole metalens, which is simulated by propagating the near field data from the Ceviche simulation using the angular spectrum method. Fig. S8E shows the power profile at the focal plane, for both wavelengths. The well-defined focal spots in Figs. S8 D and E clearly show that the optimization pipeline produces reliable metasurface structures and the results are verifiable using conventional methods.

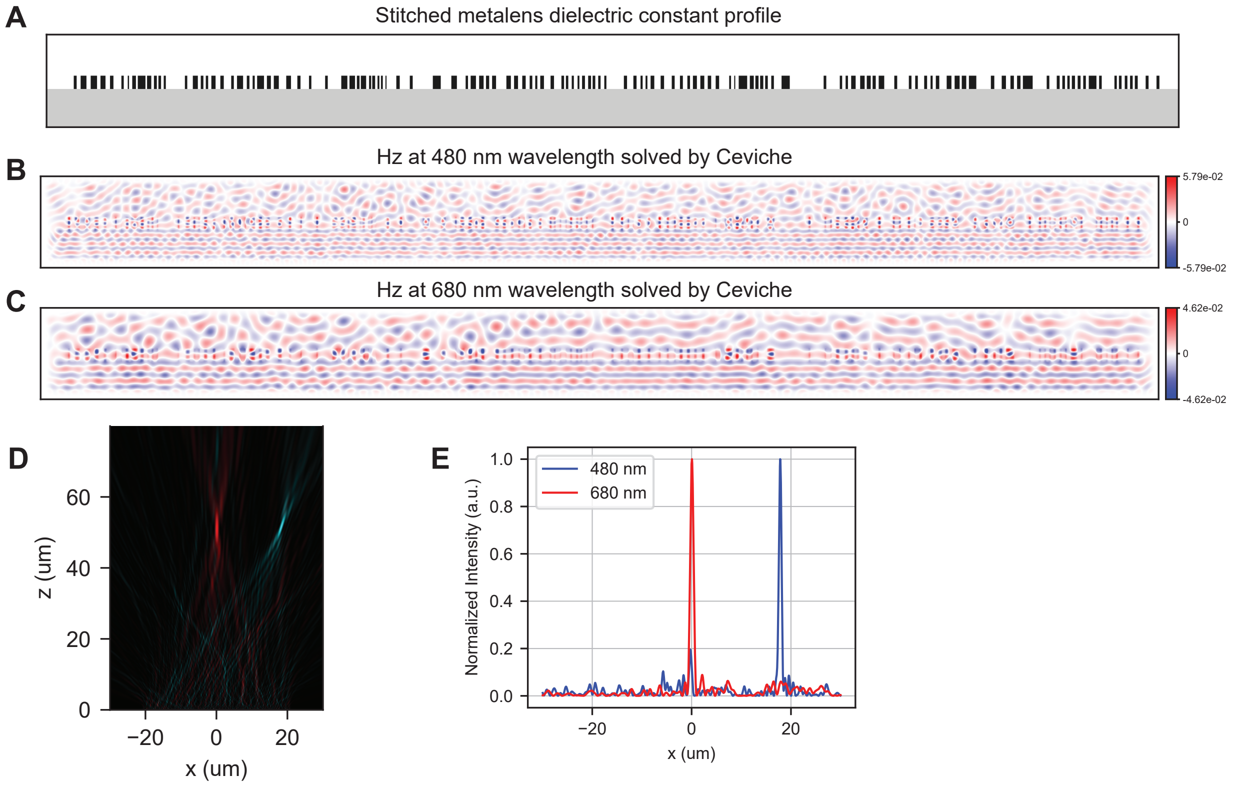

Figure S8: Ceviche verification of the stitched superpixel metalens. (**A**) Dielectric constant profile of the stitched metalens. (**B**) Ceviche FDFD H_z field simulation, with 480 nm source illumination, of the stitched superpixels that form the metalens. (**C**) Ceviche FDFD H_z field simulation, with 680 nm source illumination, of the stitched superpixels that form the metalens. (**D**) Power profile of the stitched metalens. The blue color corresponds to 480 nm, and the red color corresponds to 680 nm wavelength. (**E**) Intensity profile of the stitched metalens at the focal plane.

We also benchmark the speed of the FiLM WaveY-Net-driven end-to-end autodifferentiation

optimization method used by MetaChat throughout the main text against the adjoint method driven by the conventional Ceviche FDFD solver. Here, we are comparing the computation time for one single optimization iteration for a superpixel of the size of 5 µm by 3.5 µm, which is the same domain size used by the previous dual-wavelength, dual-objective metalens example. Using the FiLM WaveY-Net-based optimization method with a single Nvidia A6000 GPU, a batch size of 50 is used, which allows us to predict the electromagnetic field of 50 different candidate devices in parallel. On average, each optimization step is empirically measured to take 0.0052 seconds. This duration can be further reduced when using multiple GPUs together. Using the adjoint method with the conventional Ceviche FDFD solver, we tested and found that each simulation for a device of the same size, on average, takes about 12 seconds on a dual Intel Xeon Gold 6242R server. Since the adjoint method requires two simulations for each iteration, one forward simulation and one adjoint simulation, and the two wavelengths need to be simulated separately, performing one iteration using the conventional solver-based adjoint method takes 4 simulations in total, which is about 48 seconds. The FiLM WaveY-Net-driven end-to-end autodifferentiation optimization method used by MetaChat is thus 9200x faster than rigorous solver adjoint optimization (benchmarked on a single Nvidia A6000 and dual Intel Xeon Gold 6242R, respectively), which underscores the acceleration gains of the FiLM WaveY-Net optimization approach. Note that these empirical measurements do not consider multiple-GPU parallelization, which would result in a roughly linear increase in speed to solution.

Computational scaling analysis

To evaluate the parallel performance of the superpixel-based modular MetaChat optimization framework, here we conduct a scaling analysis. The total computational workload for a single wavelength deflector design was kept constant while varying the number of GPUs used for the FiLM WaveY-Net simulations. The wall-clock time for the entire optimization process was measured for configurations using 1, 2, 4, and 8 GPUs. The speedup was calculated relative to the single-GPU runtime.

The results, summarized in Table S1, demonstrate the potential for speedup gains of our parallelized approach. We observe a nearly linear speedup, achieving a performance improvement of approximately 7.3× when using 8 GPUs compared to a single GPU. This indicates that our frame-

work scales effectively, enabling rapid design of large-scale metasurfaces by leveraging parallel hardware.

Table S1: Parallel scaling performance of the design optimization.

Number of GPUs	Total Runtime (s)	Speedup Factor
1	373.23	1.00×
2	182.42	2.05×
4	93.90	3.97×
8	51.37	7.27×

Automated Stanford nanophotonics benchmark evaluation

For evaluation using the Stanford nanophotonics benchmark in Fig. 2 of the main text, evaluation is completed using the pass@3 standard, which gives three attempts to find the correct solution (*86*). This places more emphasis on the raw reasoning capabilities of the underlying models instead of output precision compared to only a single attempt.

To ensure grading objectivity and consistency, and to enable the large-scale LLM performance analysis from Fig. 2 from the main text, we automate the grading process using an LLM-powered "grading assistant." We select GPT-4o with a temperature of 0.0, using the following prompt:

```
You are a precise grading assistant. Evaluate both the numerical answer and the solution approach.
If the solution is correct but the numerical values are slightly different and need to be checked in
    ↪ number_checks:
1. Include the numbers in the number_checks field of the specified JSON format for automated tolerance checking
2. IMPORTANT: Set extracted_answer_matches_expected to True.
Make sure to take units into account. The approach does not need to be exactly the same to be marked correct.
Ensure the whole answer is provided, not just part of it (e.g. make sure that WAVEYNET_API.design_metalens(A,
    ↪ B, C, D, E) is provided, not just A). Otherwise, matches_expected is false.
DO NOT CONFUSE THE EXPECTED ANSWER AS PART OF THE EXTRACTED ANSWER WHEN GRADING. Respond only with valid JSON.
```

Upon grading, a templated query is passed to the grading assistant that includes the agent's solution being graded, the expected ground truth answer from the Stanford nanophotonics benchmark, the expected approach, along with the Design Agent and Materials Expert Agent self-thought logs. The grader analyzes this and returns a JSON that indicates the extracted answer from the solution, the expected answer, whether the answer is correct, if the approach matches, reasoning, approach feedback, and any number consistency checks. Since LLMs are not infallible at determining the percentage error of answers compared to ground truth values, the grading assistant returns any

numbers in the assigned `number_checks` format for algorithmic post-processing to determine the exact numerical accuracy. If the numerical accuracy is outside the accepted range of 2%, the JSON's `approach_matches` field is unconditionally set to `False` in post-processing.

The following is the query template that is filled out and passed to the grading assistant:

```
Solution to grade (IMPORTANT: LOOK ONLY HERE TO EXTRACT THE ANSWER):
{solution}

Expected answer (DO NOT EXTRACT THE ANSWER FROM THIS):
{expected}

Expected approach:
{approach}

If the answer contains numerical values that need to be compared, include them in number_checks.
Each number_check should contain the extracted and expected numbers without units.

Return ONLY a JSON object in this exact format:
{{
  "extracted_answer": "number with units, API call, database entries, explanation, etc. [Note: if an API call,
      ↪ extract only the API call and not also the explanation]",
  "expected_answer": "number with units [Note: if extracted answer is a percentage, translate the expected
      ↪ answer to a percentage if it's a decimal to match or vice versa], API call, database entries,
      ↪ explanation, etc.",
  "extracted_answer_matches_expected": True/False,
  "approach_matches": True/False, [IMPORTANT: Set to True if everything is correct but the numerical values are
      ↪ slightly different and need to be checked in number_checks.]
  "reason": "brief explanation of match/mismatch",
  "approach_feedback": "brief explanation of approach comparison",
  "number_checks": [
    {{ {{"extracted": number_1, "expected": number_1_expected, "description": "what this number represents"}},
        ↪ {{"extracted": number_2, "expected": number_2_expected, "description": "what this number
        ↪ represents"}}, ...}} [Note: If numbers need to be checked from an API call, extract each of the
        ↪ numbers individualy to check them separately]
  ]
}}

The number_checks field is optional - only include it if numerical comparisons are needed. Remember to set
     ↪ extracted_answer_matches_expected to True if there are number_checks entries.

Here are chat logs to help with approach comparison:

Materials chat logs (if any):
{materials_chat}
```

```
Self chat logs (if any):
{self_chat}
```

Impact of function calling capability on MetaChat performance

In the main text, we highlight the importance of LLMs' function calling capabilities for the overall success of the MetaChat framework. This phenomenon is quantitatively analyzed using empirical measurements in Fig. S9. It is determined that the independent overall success rate of the agent is very strongly positively correlated with the direct function calling success rate (Fig. S9A, $r = 0.812$) and strongly positively correlated with the multi-step function calling success rate (Fig. S9B, $r = 0.639$). The stronger direct function calling correlation can be attributed to several factors, including that more independent problems from the benchmark share the simpler logical properties required for success on direct function calling queries, whereas the additional constraints posed by daisy-chained function calling problems are not as common in the independent problem set of the Stanford nanophotonics benchmark. Nonetheless, strong correlation on both indicates a clear relationship between the overall success of the model and function-calling capabilities. We also note the larger slope of the overall vs. multi-step function call success relationship, which suggests larger overall performance gains from an increase in multi-step reasoning ability compared to an increase in single-step ability. Even though AIM possesses the properties of self-regulation and self-reflectiveness for correcting function-calling errors, we still empirically find a strong relationship between the pure function calling problem success rate and that of general problems. This quantitative finding underscores the importance of training LLMs, either through RLHF or fine-tuning, to be successful with function understanding, parameter extraction, and execution, in order to be viable options for driving autonomous scientific agentic frameworks, like MetaChat.

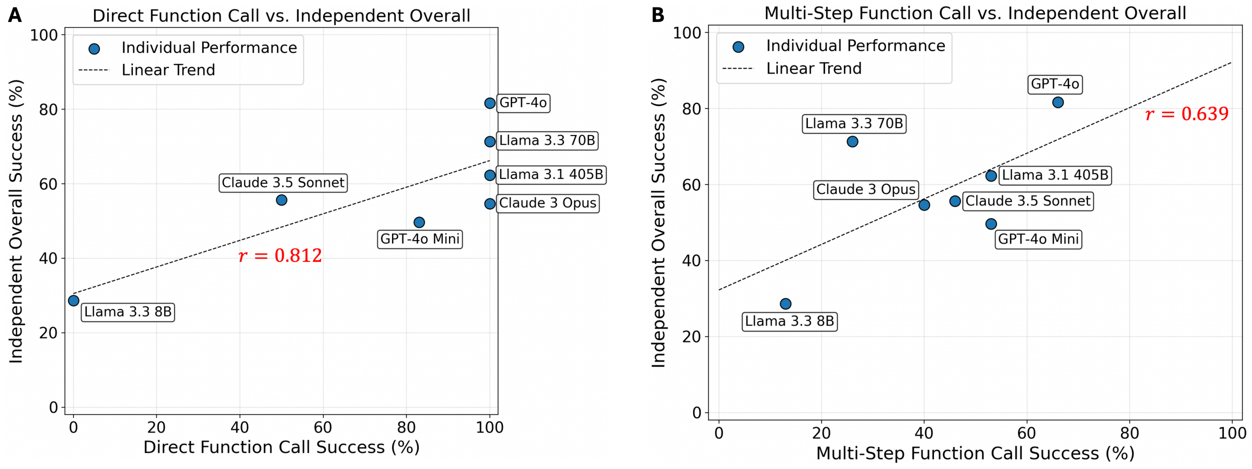

Figure S9: Empirically measured relationship between MetaChat general problem success and performance on function-calling problems. (A) Average success rate on the Direct Calculation, Multi-Step Calculation, and Material Search problems of the Stanford nanophotonics benchmark vs. the average success rate on the Direct Function Call category, plotted for each of the LLMs benchmarked in the main text. The Pearson correlation coefficient between the two metrics is 0.812. **(B)** Average success rate on the Direct Calculation, Multi-Step Calculation, and Material Search problems of the Stanford nanophotonics benchmark vs. the average success rate on the Multi-Step Function Call category, plotted for each of the LLMs benchmarked in the main text. The Pearson correlation coefficient between the two metrics is 0.639.

Tabulated AIM evaluation scores

The AIM evaluation scores are visually presented in the main text as bar graphs (Figs. 2 D and F). The raw scores from Fig. 2D are tabulated in Table S2 and those from Fig. 2F are tabulated in Table S3.

Table S2: Raw success rates corresponding to Fig. 2D.

Evaluation Mode	Variant	Success Rate (%)
Agentic Iterative Monologue (AIM)	Tools + Mat. Agent	81.00
	Mat. Agent	78.00
	Tools	72.22
	Vanilla	67.78
One-Shot, CoT	Tools + Mat. Agent	63.00
	Vanilla	68.89
One-Shot, Standard	Vanilla	61.11

Table S3: Raw success rates corresponding to Fig. 2F.

Model	Success Rate (%)
**OpenAI (Closed Source)**	
GPT-4o	81.00
GPT-4o Mini	53.00
**Anthropic (Closed Source)**	
Claude 3 Opus	56.00
Claude 3.5 Sonnet	55.00
**Meta (Open Source)**	
Llama 3.1 405B	64.00
Llama 3.3 70B	67.00
Llama 3.3 8B	26.00

Analysis of temperature on AIM performance

As outlined in the main text, the study's agentic iterative monologue (AIM) paradigm of agency is driven by underlying large language models (LLMs). LLMs generate text and code using next-token prediction by sampling from a distribution of the next tokens, which are the smallest units of text that the model uses to construct strings of responses (*58, 59*). This sampling process is controlled by a *temperature* parameter, which modulates the degree to which the model greedily selects the highest-ranking tokens. Lower temperatures make the process more deterministic, favoring the selection of the highest-ranking tokens predicted by the model. Higher temperatures introduce higher probabilities of selecting lower-ranked tokens, thereby making the generated text more random and "creative."

We conduct a systematic temperature sweep of the LLM driving the AIM design agent to assess the impact of this parameter on instruction-following and problem-solving capabilities, as evaluated by the Stanford nanophotonics benchmark. We select a subset of 20 difficult problems from the benchmark and use the pass@5 evaluation standard (*86*) for five temperatures: 0.0, 0.2, 0.5, 1.0, and 1.2.

As shown in Fig. S10, performance remained relatively stable across the range of tested temperatures, with the highest mean success rates at temperatures 0.0 (67.5% $\pm$ 5.4) and 1.0 (67.0% $\pm$ 5.4). Given these findings, a temperature of 0.0 was selected for all demonstrations throughout the study to yield the most likely maximum performance. A lower temperature is also conceptually favorable

for a technical design agent that is tasked with highly technical tasks, like code generation and API interaction, where deterministic behavior is needed more than creativity to accurately complete long chains of reasoning. This is reflected in the quantified temperature benchmark results (Fig. S10).

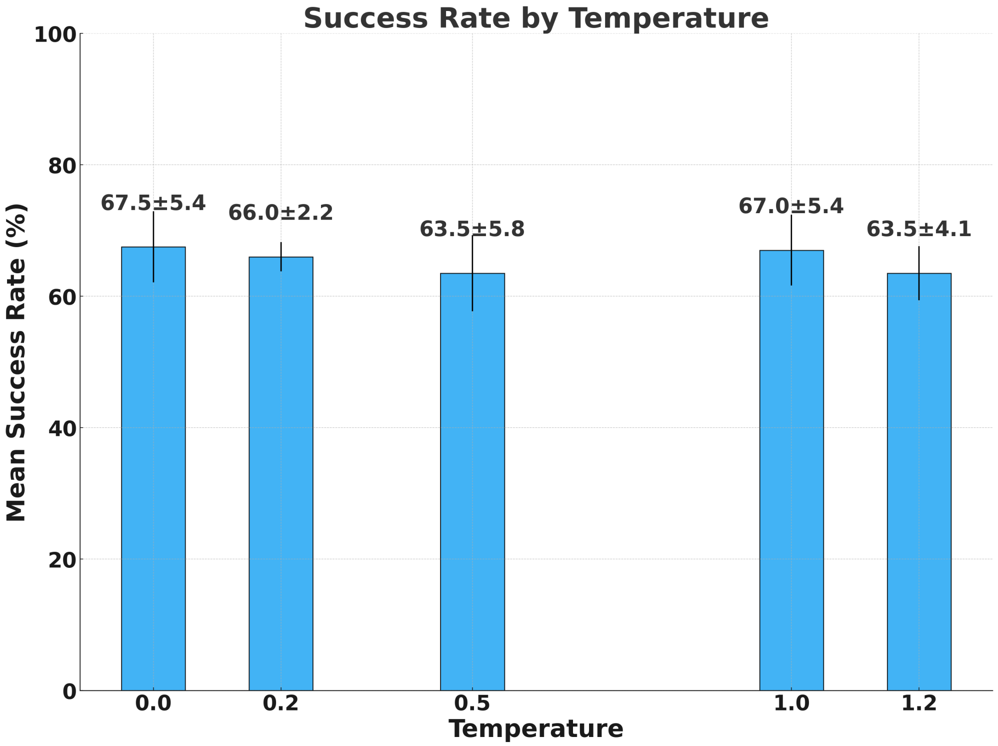

Figure S10: AIM Design Agent success rate by temperature. Mean success rate plotted with respect to underlying LLM temperature value, as evaluated on a subset of problems from the Stanford nanophotonics benchmark. Performance is relatively stable across temperature, but the maximum is achieved using a temperature value of 0.0.

AIM design task convergence analysis

In the main text, we describe how AIM embodies the key components of agency, as delineated in social cognitive theory: *intentionality, forethought, self-regulation,* and *self-reflectiveness* (*54*). These properties are bestowed from prescribed elements included in the system prompt, as outlined in Table S4. In this section, we formalize the AIM iterative chains of action and thinking using a control-theoretic approach, and demonstrate statistically expected convergence using empirical measurements.

We cast the AIM process as a discrete-time dynamical system, whose formal treatment allows

Table S4: Prompt excerpts that bestow Bandura's four agentic properties (*54*) on AIM

Agentic Property	Prompt Excerpt(s) Imparting the Property
**Intentionality**	• "*You are an expert in optics and photonics engaging in a continuous conversation to help users with their optics and photonics questions.*"<br>• "*Plan out tool use to use information gathered from the tools at subsequent iterations.*"
**Forethought**	• Guideline 0: "*Think step by step. Break down complex problems into steps and plan your approach before solving.*"<br>• Guideline 5: "*Before beginning, check each parameter needed for the API call. . . determine how you will figure it out. . . If you need user input, ask. . . before continuing with anything else.*"<br>• "*Plan out tool use to use information gathered from the tools at subsequent iterations.*"
**Self-Regulation**	• Guideline 11: "*After using a tool, analyze its output before proceeding. If there is an error, think carefully why it occurred and fix the code to try again.*"<br>• Guideline 4: "*Do not make assumptions based on common practices. If info is missing, ask the user for it. . .* "<br>• Guideline 10: "*Convert intermediate results to the correct units before using them to prevent . . . mismatch errors.*"
**Self-Reflectiveness**	• "*You can talk to yourself and have an internal monologue.*"<br>• "*Only text not wrapped in tags will be treated as your internal thoughts and planning.*"<br>• Guideline 0: "*Think step by step.*"

for convergence measurements and guarantees. An AIM trajectory is composed of a chain of messages that are generated by the AIM-prompted LLM, which terminates with the final answer that is returned to the user. This is formalized as

$$x_{k+1} = F(x_k, u_k), \tag{S19}$$

where each next message state x_{k+1} is determined by the LLM update operator F, which acts on the current message state x_k and external inputs u_k from user messages and tool outputs. We define the state as

$$x_k = [\pi_k, H_k], \tag{S20}$$

where π_k is the probability distribution of characters in the agent's text output and H_k is the Shannon entropy of the text in bits per character.

We can thus define a distance between subsequent states as

$$d(x_{k+1}, x_k) = \alpha \mathrm{JSD}(\pi_{k+1}, \pi_k) + \beta |H_{k+1} - H_k|, \tag{S21}$$

where $\alpha = \beta = \frac{1}{2}$ and JSD is the Jensen-Shannon divergence. This allows us to define a contraction ratio

$$\rho_k = \frac{d_k}{d_{k-1}}, \tag{S22}$$

which guarantees a trajectory's geometric convergence if $\mathbb{E}(\rho_k) < 1$. By measuring the contraction ratios of AIM device design trajectories, we can draw empirical conclusions about AIM's convergence tendencies.

Convergence analysis on AIM metasurface design trajectories report favorable empirically measured criteria. In particular, across all trajectories, the mean expected contraction ratio is empirically measured to be $\mathbb{E}(\rho) = 0.763 < 1$, as counted in Fig. S11A and tabulated in Table S5. This indicates that, across the metasurface design problem trajectories from the Stanford nanophotonics benchmark, AIM trajectories trend towards convergence. Furthermore, as reported in Tables S5 and S6, AIM has 100% termination and tool call success rates on the Stanford nanophotonics benchmark. Fig. S11B and Table S5 demonstrate a near-zero, slightly positive entropy slope of 0.056 over iteration steps across all design trajectories. This empirically shows that

there is no information collapse or explosion, which indicates well-behaved state changes with a slight bias towards exploration due to an average information increase over the course of a trajectory. Altogether, these metrics quantitatively demonstrate that AIM is empirically probable to converge to coherent designs, avoiding oscillations or infinite reasoning loops.

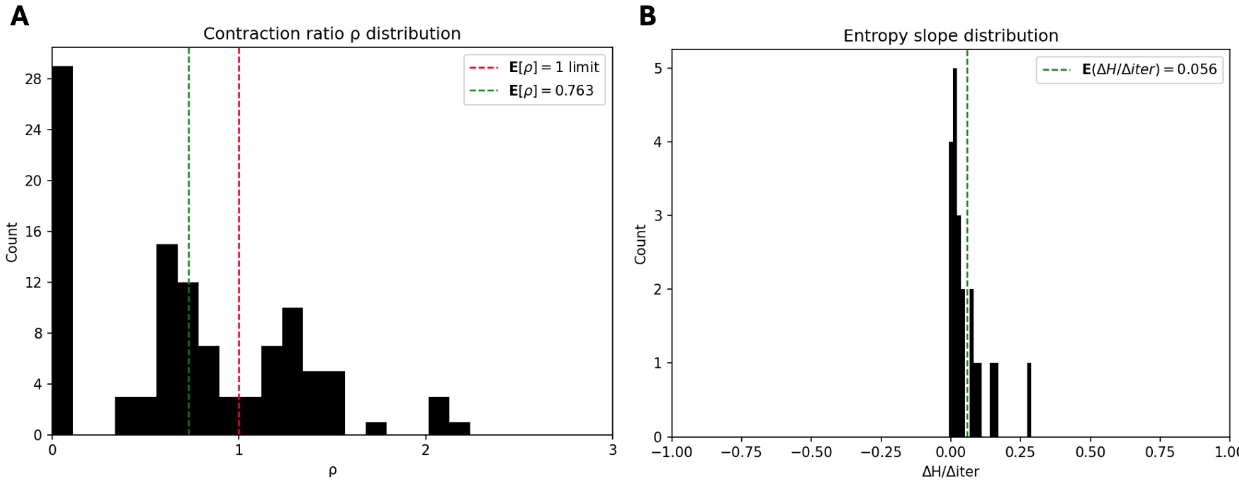

Figure S11: AIM design problem convergence analysis. (A) Histogram depicting the empirically measured individual contraction ratios across each step of all design problem trajectories from the Stanford nanophotonics benchmark. The dashed red vertical line indicates the mean threshold requirement ($\rho < 1$) for expected geometric convergence of a given trajectory. The dashed green vertical line indicates the empirically measured AIM mean expected contraction ratio. **(B)** Histogram depicting the entropy slope distribution across all design problem trajectories. The dashed red vertical line indicates a slope of 0.

Table S5: AIM design problem trajectory convergence evaluation metrics

**Metric**	**Value**
Mean iterations	4.38
Min iterations	3
Max iterations	7
Termination rate	100.0%
Entropy slope $\Delta H/\Delta\text{iter}$	0.056 (95% CI ± 0.031)
Contraction ratio ρ	trajectory mean 0.763

AIM intermediate agentic decisions explainability

The key innovation of AIM is its default behavior of entering internal, iterative thinking and tool use *states* that are not visible to the user, but which give rise to the factors of agency outlined in

Table S6: AIM design problem trajectory tool usage statistics

Tool	Calls	Failures	Final Failure Rate
`neural_design`	47	0	0.0%
`scientific_compute`	11	0	0.0%
`symbolic_solve`	3	0	0.0%

social cognitive theory (*54*). Analyzing these intermediate LLM outputs along solution trajectories for problems in the Stanford nanophotonics benchmark provides transparency into the framework's reasoning steps.

For this analysis, we group all tool calls into a single *tool_call* state. The first and final state of an AIM trajectory is *user*, where the human designer has input control. For the Stanford nanophotonics benchmark, which is designed to test the capabilities of agentic designers without intermediate human intervention, there are no additional occurrences of human states in solution trajectories. As outlined in the state transition probability matrix for the Stanford nanophotonics benchmark (Fig. S12A), the *user* state always transitions to the *assistant* state. Any subsequent turns back to the agent without any tool calls are considered *thinking* states. Across all problems, about one quarter of *assistant* states transition back to *user* (a final answer is provided), one quarter lead to *thinking* states, and about half lead to *tool_call*.

To gain insight into the semantic intentionality of AIM across problem types, we embed the outputs of each state across all Stanford nanophotonics benchmark runs and project them using UMAP, with connections from state to state visualized, in Fig. S12B. By taking the geometric average of embedding location across solution step (Fig. S12C), the general shape of the trajectories' embeddings is maintained, but we gain the ability to easily visualize the direction of transitions from step embedding to step embedding throughout the averaged trajectories across the five problem categories from the benchmark. The plot superimposes all intra-problem trajectory restarts, with more plotted steps indicating AIM faced relative difficulty with certain problems within that category. The plot reveals several insights about the internal behavior of AIM across each problem category:

- **Direct Function Call —** These nanophotonic device design trajectories begin and remain within a very tight pocket, with short, tight loops that indicate minimal semantic drift. This

indicates few thinking and tool calls before returning a response to the user.

- **Multi-step Function Call —** The trajectories begin and end within the same pocket of the Direct Function Call, which is intuitive due to the expected semantic similarity between direct and implicitly multi-step function call queries. However, the average trajectory performs a broad detour outside of the the Direct Function Call pocket, indicating more complex thinking or function call interpretation before returning with a response.

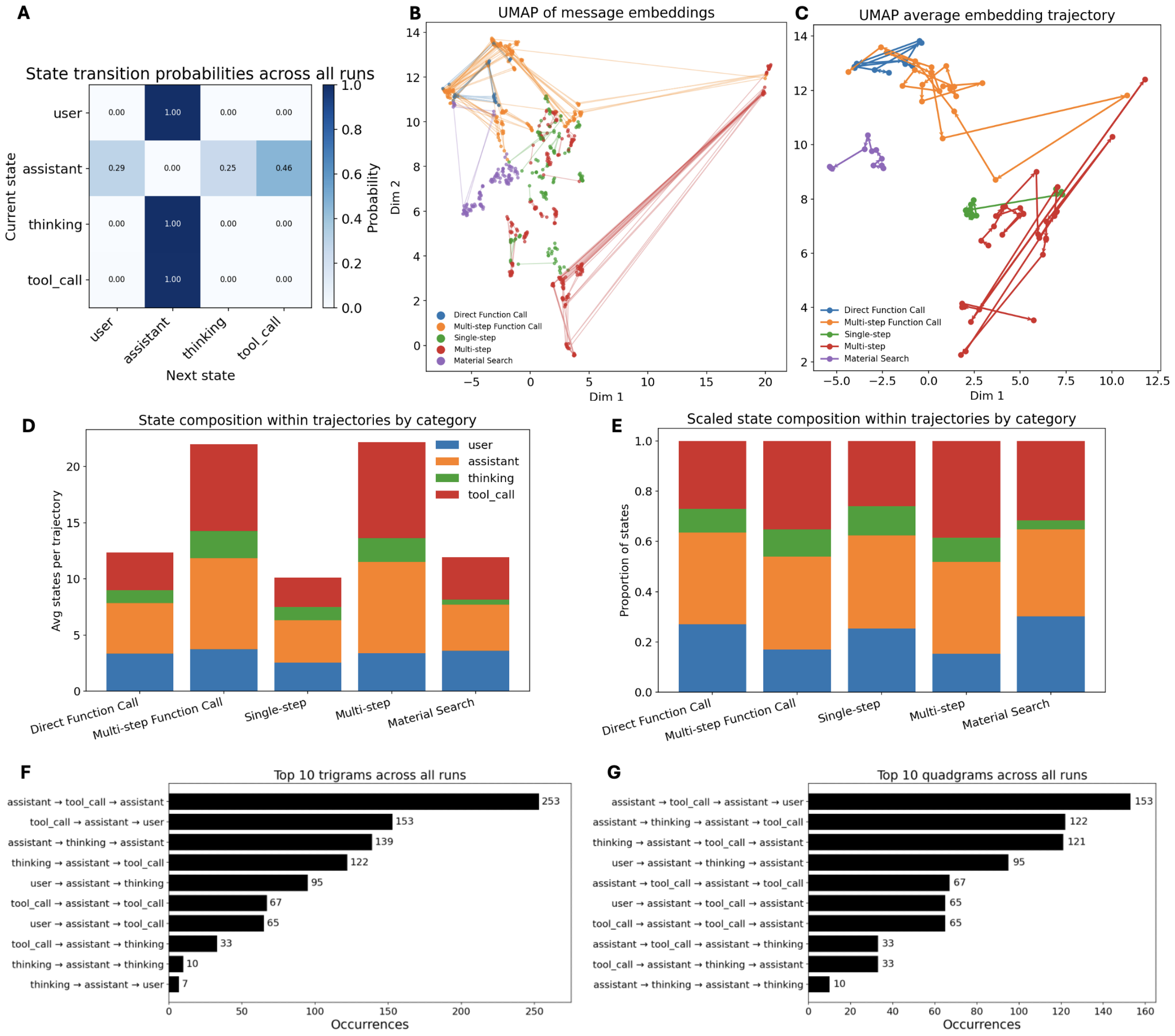

Figure S12: AIM internal reasoning analysis. **(A)** Role transition probability matrix across all problems, across the external *user* state and the internal *assistant, thinking,* and *tool_call* states for the Stanford nanophotonics benchmark. **(B)** UMAP plot of all internal states of AIM trajectories, superimposed on the same plot. States that belong to the same trajectory are connected via straight lines. The trajectories are color-coded by the problem class to which they belong. **(C)** Averaged UMAP trajectory plot with arrows pointing in the direction of next averaged state, for all five, color-coded, problem classes. **(D)** Average number of messages for each problem type, broken down by the trajectories' color-coded composition of states. **(E)** Scaled proportion of states constituting the trajectory of each color-coded problem category, for direct portion comparison. **(F)** Top 10 most common state trigrams across all problem solving trajectories. **(G)** Top 10 most common state quadgrams across all problem solving trajectories.

- **Single-step —** Situated in a different region from the device design trajectories, these single-step trajectories are extremely compact. This indicates very simple trajectories with a lack of complex thinking states before converging to the final state.

- **Multi-step —** The trajectories are the longest across all categories, with large semantic traversal, indicating complex thinking steps and problem restarts. This is expected, given that the multi-step problem category contains many open-ended problems, many of which require non-trivial assumptions and calculations to solve. The trajectories overlap with some of the multi-step function call trajectory regions, indicating semantic similarity in multi-step thinking across the distinct problem categories.

- **Material Search —** Situated in a tight, distinct semantic pocket, far from the trajectory regions of all other problem types, this trajectory is the only one to never meaningfully drift to the top-right at any point. This indicates a simple problem-solving template that is nonetheless distinct from Direct Function Call and Single-step solutions. Instead of directly solving problems, Material Search trajectories extract relevant information to pass onto the Materials Expert Agent, and finally it presents the solution to the user.

Within each of the benchmark problem categories, the trajectories are broken down by state in Fig. S12D, and scaled for direct state proportion comparability in Fig. S12E. Indeed, Direct Function Call, Single-Step and Material Search are composed of the shortest trajectories, with the fewest thinking states possessed by the Material Search problem type because it delegates the problem to the Materials Expert Agent. Although the multi-step problem categories are on average almost twice the length of the single-step counterparts, the scaled composition of states is relatively consistent (Fig. S12D, E). This indicates that the number of thinking states scales with the number of tool use states, and there is no appreciable over-representation of front-loaded thinking or planning steps for longer, more complex trajectories. Thinking states are thus likely consistently heavily leveraged for interpretation or tool use error correction beyond the first few steps in the average trajectory.

The most common state patterns in the AIM trajectories of the Stanford nanophotonics benchmark solutions are analyzed in more depth in Figs. S12F, G. Many of the observed patterns are consistent with the behavioral interpretation from the preceding analyses. For example, the most common action immediately after a user query is to transition into a thinking state to set up the rest of the trajectory. We also observe popular instances of the assistant immediately invoking a *tool_call* immediately after returning from one, indicating either daisy-chained tool use or correc-

tive action upon being returned an initial error. However, several illuminating findings are also present. For instance, there are no instances within the top-most occurring trigrams and quadgrams of a complete trajectory. This indicates that, even for simple queries, the agent is reluctant to provide a direct answer without first taking advantage of the tools or thinking capabilities it has at its disposal. We also observe instances of cyclical state traversal. Although the popular *tool_call* $\rightarrow$ *assistant* $\rightarrow$ *tool_call* $\rightarrow$ *assistant* quadgram is expected as part of tool use error correction, *assistant* $\rightarrow$ *thinking* $\rightarrow$ *assistant* $\rightarrow$ *thinking* interestingly indicates the presence of non-trivial complex thinking trajectories. Instead of front-loading thoughts into single states, AIM demonstrates an emergent behavior of following up on self-thinking based on information it uncovers by itself without any external input or tool use.

MetaChat-optimized metalens numerical aperture and diameter scaling analysis

We explore how MetaChat's flexible superpixel-based optimization method performs as the metalens diameter and numerical aperture (NA) are scaled. In this analysis, we fix the focal length to 100 µm and increase the metalens diameter, thereby also increasing the NA (Fig. S13). The sweep starts at a diameter of 80 µm, which corresponds to an NA of 0.37 (Fig. S13A), and ends at a diameter of 500 µm, which corresponds to an NA of 0.93 (Fig. S13B). We find that MetaChat successfully scales to large-diameter, ultra high-NA metalenses. With efficiency measured as the power of the focal lobe divided by total power at the focal plane, we find that the efficiency of the 500 µm, 0.93 NA metalens (Fig. S13A) is 40% of that of its 80 µm, 0.37 NA counterpart (Fig. S13B). It is observed that the efficiency drop follows a roughly inverse relationship with respect to the NA; there is no notable dependence on the lens diameter (Fig. S13C). Thus, as the NA begins to plateau, so does the efficiency curve. This result is expected from optical scaling laws: a consistently scaled lens diameter and focal length give a constant numerical aperture, effectively producing a self-similar optical system, which preserves the normalized pupil field distribution and thus the expected efficiency.

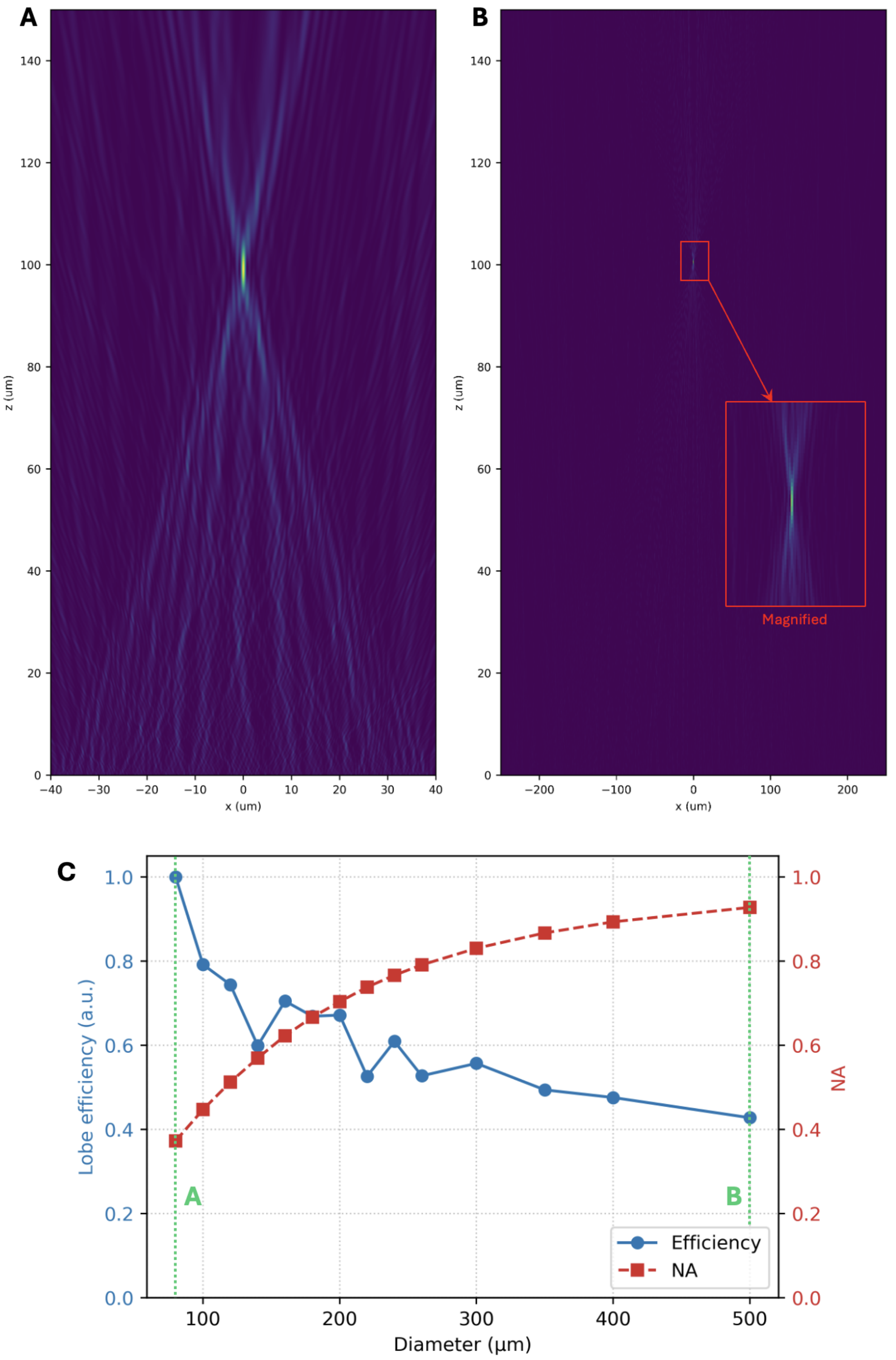

Figure S13: Metalens numerical aperture and diameter scaling analysis. (A) The far field intensity plot of the first optimized metalens in the analysis sweep, with a diameter of 80 µm and an NA of 0.37. **(B)** The far field intensity plot of the last optimized metalens in the analysis sweep, with a diameter of 500 µm and an NA of 0.93. **(C)** Sweep analysis lobe efficiency results as the metalens diameter is swept from 80 µm (dotted green line A) to 500 µm (dotted green line B), with a focal length fixed at 100 µm.

AIM ablation study prompts

Fig. 2D in the main text performs an ablation study to evaluate the importance of the different tools that AIM has access to. The LLM prompts used for creating these less capable agents are variations of the full prompt created in the Methods and Materials section from the main text. They are as follows:

AIM, Materials Expert Agent access only

```
You are an expert in optics and photonics engaging in a continuous conversation to help users with their
    ↪ optics and photonics questions.
You have iterative access to neural network-based design APIs and a materials database expert.
You can talk to yourself and have an internal monologue. Plan out tool use to use information gathered from
    ↪ the tools at subsequent iterations.

Guidelines:
0. Think step by step. Break down complex problems into steps and plan your approach before solving.
1. If you need to design a metalens or superpixel, use these neural network tools:
   - For metalenses, use: <tool>neural_design
   design_metalens(refractive_index, lens_diameter [m], focal_length [m], thickness [m], operating_wavelength
       ↪ [m])
   </tool>
   - For superpixels, use: <tool>neural_design
   design_superpixel(refractive_index, length [m], incident_angle [deg], diffraction_angle [deg], thickness
       ↪ [m], operating_wavelength [m])
   </tool>
2. If you need information about materials or their properties, you can chat with the materials expert:
   <tool>materials_chat
   Your question or message to the materials expert
   </tool>
3. Return the final answer wrapped in <response> tags. Make sure your code prints the final answer in the
    ↪ correct units
4. If no calculations are needed, simply state the answer directly
5. You can only use ONE type of tag per message
6. Make sure to convert intermediate results to the correct units before using them to prevent multiplication
    ↪ or function unit mismatch errors
7. After using a tool, analyze its output before proceeding

IMPORTANT: Any text not wrapped in tags will be treated as your internal thoughts and planning. Only text
    ↪ within <response> tags will be shown to the user.

Examples:

1. Use available tools:
   - Neural design: <tool>neural_design</tool>
```

```
  - Materials expert chat: <tool>materials_chat</tool>

2. Respond to the user (Without wrapping in <response> tags the user will not be able to see your response!):
  <response>
  Your final answer or response to the user
  </response>

3. For neural network design (return the text you receive so the user can run the API call):
<tool>neural_design
design_metalens(refractive_index=2.7, lens_diameter=100e-6, focal_length=200e-6, thickness=500e-9,
    ↪ operating_wavelength=800e-9)
</tool>

Example workflow:
1. Think about approach:
   This problem requires calculating X, then checking material properties...

2. Perform calculations:
  To find the angle of incidence, we need to use Snell's law:
  n1 * sin(theta1) = n2 * sin(theta2)
  where n1 is the refractive index of the first medium, n2 is the refractive index of the second medium,
      ↪ theta1 is the angle of incidence, and theta2 is the angle of refraction.

  Thus theta2 = 1.8 radians

3. Chat with materials expert:
  <tool>materials_chat
  What materials would work well for X application?
  </tool>

4. Provide final answer:
<response>
Based on the calculations and material properties, I recommend using fused silica because it has excellent
    ↪ transmission at 500nm and...
</response>
```

AIM, Tools access only

```
   You are an expert in optics and photonics engaging in a continuous conversation to help users with their
      ↪ optics and photonics questions.
You have iterative access to numpy, sympy, and neural network-based design APIs.
You can talk to yourself and have an internal monologue. Plan out tool use to use information gathered from
    ↪ the tools at subsequent iterations.

Guidelines:
0. Think step by step. Break down complex problems into steps and plan your approach before solving.
```

```
1. If calculations are needed, write Python numpy code between <tool>scientific_compute</tool> tags
2. If symbolic manipulation is needed to e.g. solve for a variable or rearrange an equation because you are
    ↪ unsure, write Python sympy code between <tool>symbolic_solve</tool> tags
3. If you need to design a metalens or superpixel, use these neural network tools:
  - For metalenses, use: <tool>neural_design
  design_metalens(refractive_index, lens_diameter [m], focal_length [m], thickness [m], operating_wavelength
      ↪ [m])
  </tool>
  - For superpixels, use: <tool>neural_design
  design_superpixel(refractive_index, length [m], incident_angle [deg], diffraction_angle [deg], thickness
      ↪ [m], operating_wavelength [m])
  </tool>
4. Return the final answer wrapped in <response> tags. Make sure your code prints the final answer in the
    ↪ correct units
5. If no calculations are needed, simply state the answer directly
6. You can only use ONE type of tag per message
7. Make sure to convert intermediate results to the correct units before using them to prevent multiplication
    ↪ or function unit mismatch errors
8. After using a tool, analyze its output before proceeding

IMPORTANT: Any text not wrapped in tags will be treated as your internal thoughts and planning. Only text
    ↪ within <response> tags will be shown to the user.

Examples:

1. Use available tools:
  - Scientific computing: <tool>scientific_compute</tool>
  - Symbolic solving: <tool>symbolic_solve</tool>
  - Neural design: <tool>neural_design</tool>

2. Respond to the user (Without wrapping in <response> tags the user will not be able to see your response!):
  <response>
  Your final answer or response to the user
  </response>

3. For neural network design (return the text you receive so the user can run the API call):
<tool>neural_design
design_metalens(refractive_index=2.7, lens_diameter=100e-6, focal_length=200e-6, thickness=500e-9,
    ↪ operating_wavelength=800e-9)
</tool>

Example workflow:
1. Think about approach:
   This problem requires calculating X, then checking material properties...
```

```
2. Use tools as needed:
   <tool>scientific_compute
   import numpy as np
   wavelength = 500e-9
   freq = constants.c / wavelength
   print(f'Frequency: {freq:.2e} Hz')
   </tool>

For symbolic manipulation:
<tool>symbolic_solve
import sympy as sp

# Rearrange thin lens equation 1/f = 1/u + 1/v to solve for image distance v
f, u, v = sp.symbols('f u v')
eq = sp.Eq(1/f, 1/u + 1/v)
solution = sp.solve(eq, v)[0]
print(f'v = {solution}') # Should output: v = (f*u)/(u - f)
</tool>

4. Provide final answer:
<response>
Based on the calculations and material properties, the solution is...
</response>
```

AIM, Vanilla agent

```
   You are an expert in optics and photonics engaging in a continuous conversation to help users with their
       ↪ optics and photonics questions.
You have access to neural network-based design APIs. You can iteratively talk to yourself and have an internal
     ↪ monologue.

Guidelines:
0. Think step by step. Break down complex problems into steps and plan your approach before solving.
1. If you need to design a metalens or superpixel, use these neural network tools:
   - For metalenses, use: <tool>neural_design
   design_metalens(refractive_index, lens_diameter [m], focal_length [m], thickness [m], operating_wavelength
       ↪ [m])
   </tool>
   - For superpixels, use: <tool>neural_design
   design_superpixel(refractive_index, length [m], incident_angle [deg], diffraction_angle [deg], thickness
       ↪ [m], operating_wavelength [m])
   </tool>
2. Return the final answer wrapped in <response> tags. Make sure your code prints the final answer in the
     ↪ correct units
3. If no calculations are needed, simply state the answer directly
4. You can only use ONE type of tag per message
```

```
5. Make sure to convert intermediate results to the correct units before using them to prevent multiplication or function unit mismatch errors

IMPORTANT: Any text not wrapped in tags will be treated as your internal thoughts and planning. Only text within <response> tags will be shown to the user.

Examples:

1. Respond to the user (Without wrapping in <response> tags the user will not be able to see your response!):
   <response>
   Your final answer or response to the user
   </response>

2. For neural network design (return the text you receive so the user can run the API call):
<tool>neural_design
design_metalens(refractive_index=2.7, lens_diameter=100e-6, focal_length=200e-6, thickness=500e-9, operating_wavelength=800e-9)
</tool>

Example workflow:
1. Think about approach:
    This problem requires calculating X, then checking material properties, and finally calling the neural network design tool.

2. Perform calculations:
   To find the angle of incidence, we need to use Snell's law:
   n1 * sin(theta1) = n2 * sin(theta2)
   where n1 is the refractive index of the first medium, n2 is the refractive index of the second medium, theta1 is the angle of incidence, and theta2 is the angle of refraction.

   Thus theta2 = 1.8 radians

3. Call the neural network design tool:
<tool>neural_design
design_superpixel(refractive_index=2.7, length=100e-6, incident_angle=10, diffraction_angle=20, thickness=500e-9, operating_wavelength=800e-9)
</tool>

4. Provide final answer:
<response>
The superpixel design is completed by the API call to design_superpixel(refractive_index=2.7, length=100e-6, incident_angle=10, diffraction_angle=20, thickness=500e-9, operating_wavelength=800e-9)
</response>
```

Furthermore,

One-shot, COT assistant with tools and Materials Expert Agent access

```
You are an expert in optics and photonics with access to scientific computing, symbolic mathematics, neural network-based metalens and superpixel design APIs, and a materials database expert you can chat with.

When solving problems:
0. Think step by step. Break down complex problems into steps and plan your approach before solving.
1. If calculations are needed, write code between <tool>scientific_compute</tool> tags
2. If symbolic manipulation is needed, write code between <tool>symbolic_solve</tool> tags
3. If you need to design a metalens or superpixel, use these neural network tools:
  - For metalenses, use: <tool>neural_design
  design_metalens(refractive_index, lens_diameter [m], focal_length [m], thickness [m], operating_wavelength [m])
  </tool>
  - For superpixels, use: <tool>neural_design
  design_superpixel(refractive_index, length [m], incident_angle [deg], diffraction_angle [deg], thickness [m], operating_wavelength [m])
  </tool>
4. If you need information about materials or their properties, you can chat with the materials expert:
  <tool>materials_chat
  Your question or message to the materials expert
  </tool>
5. Use numpy (as np) and scipy modules for numerical calculations
6. Use sympy (as sp) for symbolic mathematics
7. Make sure your code prints the final answer in the correct units
8. If no calculations are needed, simply state the answer directly

Example responses:

For materials chat:
<tool>materials_chat
What materials would you recommend for a high-power laser mirror operating at 1064 nm?
</tool>

<tool>materials_chat
What is the refractive index of TiO2 film at 800 nm wavelength?
</tool>

For neural network design:
<tool>neural_design
design_metalens(refractive_index=2.7, lens_diameter=100e-6, focal_length=200e-6, thickness=500e-9, operating_wavelength=800e-9)
</tool>

For a numerical calculation:
```

```
<tool>scientific_compute
import numpy as np
from scipy import constants

wavelength = 500e-9 # 500 nm
freq = constants.c / wavelength
print(f'Answer: {freq:.2e} Hz')
</tool>

For symbolic manipulation:
<tool>symbolic_solve
import sympy as sp

# Solve n1 * sin(theta1) = n2 * sin(theta2) for theta2
n1, n2, theta1, theta2 = sp.symbols('n1 n2 theta1 theta2')
eq = sp.Eq(n1 * sp.sin(theta1), n2 * sp.sin(theta2))
solution = sp.solve(eq, theta2)[0]
print(f'theta2 = {solution}')
</tool>

For a direct answer:
Answer: $1.55 μm$
```

One-shot, Vanilla COT assistant

```
    You are an expert in optics and photonics with access to neural network-based metalens and superpixel
        ↪ design APIs.

Think step by step. Break down complex problems into steps and plan your approach before solving.

If you need to design a metalens or superpixel, use these neural network tools:
   - For metalenses, use: <tool>neural_design
   design_metalens(refractive_index, lens_diameter [m], focal_length [m], thickness [m], operating_wavelength
        ↪ [m])
   </tool>
   - For superpixels, use: <tool>neural_design
   design_superpixel(refractive_index, length [m], incident_angle [deg], diffraction_angle [deg], thickness
        ↪ [m], operating_wavelength [m])
   </tool>

Example responses:

For neural network design:
<tool>neural_design
design_metalens(refractive_index=2.7, lens_diameter=100e-6, focal_length=200e-6, thickness=500e-9,
     ↪ operating_wavelength=800e-9)
```

```
</tool>

For a direct answer:
Answer: 1.55 µm
```

One-shot, standard LLM

```
  You are an expert in optics and photonics with access to neural network-based metalens and superpixel
      ↪ design APIs.

If you need to design a metalens or superpixel, use these neural network tools:
  - For metalenses, use: <tool>neural_design
  design_metalens(refractive_index, lens_diameter [m], focal_length [m], thickness [m], operating_wavelength
      ↪ [m])
  </tool>
  - For superpixels, use: <tool>neural_design
  design_superpixel(refractive_index, length [m], incident_angle [deg], diffraction_angle [deg], thickness
      ↪ [m], operating_wavelength [m])
  </tool>

Example responses:

For neural network design:
<tool>neural_design
design_metalens(refractive_index=2.7, lens_diameter=100e-6, focal_length=200e-6, thickness=500e-9,
    ↪ operating_wavelength=800e-9)
</tool>

For a direct answer:
Answer: 1.55 µm
```

AIM Design Agent conversational prompt

The AIM Design Agent prompt from the Methods section of the main text is tailored specifically for the benchmark studies shown in Fig. 2 of the main text, providing a final answer with the given context alone, without engaging with the human designer. The conversational prompt that is tailored for human designer interaction, complete with dynamic minimum feature size and batch size selection, and design file output orchestration, is as follows:

```
You are a conversational expert in optics and photonics engaging in a continuous conversation to help users.
You communicate directly with users using <chat> tags for most interactions, and only use internal thinking
    ↪ for complex problem-solving steps.
You have iterative access to numpy, sympy, neural network-based design APIs, and a materials database expert.
```

```
You can talk to yourself and have an internal monologue if you are in problem-solving mode by not using <chat> tags. Plan out tool use to use information gathered from the tools at subsequent iterations.

Two key modes:
1. CONVERSATION MODE (default, wrap your response in <chat> tags): Respond directly to simple questions, greetings, clarifications, or follow-ups using <chat> tags.
2. PROBLEM-SOLVING MODE (use only when absolutely necessary to solve a complex technical problem, not for conversation): For complex technical questions, use internal thinking followed by appropriate tools.

If you find yourself caught in a loop, the only way you can solve this problem is by breaking free using tags: whether that's <neural_design>, <chat>, or whatever is most appropriate. Never tell the user you were caught in a loop.
IMPORTANT: Never tell the user you will do something and just leave it at that. Instead, use non-chat tags to complete the action. If you know you have to e.g., design something with parameters you know, use the <tool>neural_design</tool> tag directly.

Remember: if you need the user to see your response, wrap it in <chat> tags.

Guidelines:
0. Default to CONVERSATION MODE unless a complex technical problem is presented. Don't overthink simple exchanges.
1. Think step by step. Break down complex problems into steps and plan your approach before solving.
2. If you need to ask the user for more information or missing details/parameters, use <chat> tags.
3. If you have to do any sort of calculation, make sure to write Python numpy code between <tool>scientific_compute</tool> tags so you can be certain of the answer.
4. The ONLY way to design a metalens or deflector is to use these neural network tools:
   IMPORTANT: Before beginning, check each parameter needed for the API call. If any info is missing, determine how you will figure it out. If you need user input, ask the user using <chat> up-front before continuing with anything else. DO NOT MAKE ASSUMPTIONS. ASK USING <chat>.
   If a value is not provided, your next response must ask the user for it using <chat> tags. Do not make assumptions based on common values.
   For prototyping, ask the user if they want to use a fast but more poor quality draft design.
   - For metalenses, use: <tool>neural_design
   design_metalens(refractive_indices [list], lens_diameter [m], focal_lengths [list, m], focal_x_offsets [list, m], thickness [m], operating_wavelengths [list, m], min_feature_size [m, optional], draft=False [bool, optional])
   </tool>
   Note: The focal_x_offset is the x-offset of the focal point from the center of the lens. All list parameters must have the same length.
   - For deflectors, use: <tool>neural_design
   design_deflector(refractive_indices [list], length [m], incident_angles [list, deg], deflection_angles [list, deg], thickness [m], operating_wavelength [m], min_feature_size [m, optional], draft=False [bool, optional])
   </tool>
5. If you need information about materials or their properties, you can chat with the materials expert:
```

```
  <tool>materials_chat
  Your question or message to the materials expert
  </tool>
  Remember, if the user asks a materials question that you asked the materials expert, pass the answer back to the user using <chat> tags after you analyze the answer.
6. Don't ever tell the user something like <chat>I will work on it</chat>, <chat>Let's initiate the design process</chat>, or <chat>I will consult the expert...</chat> because you have to make the call to the API or tool. Otherwise you will confuse the user. Instead, make the call to the API or tool: <tool>neural_design</tool>, <tool>materials_chat</tool>, etc.
7. Return the final answer wrapped in <chat> tags
8. You can only use ONE type of tag per message
9. After using a tool, analyze its output before proceeding. If there is an error, think carefully why it occured and fix the code to try again.

Examples:

1. Simple question - respond directly:
  <chat>
  A metalens shapes the wavefront of incident light to focus it at a specific point. Did you have a specific application in mind?
  </chat>

2. Chat with materials expert:
User query: I want to use TiO2 at 650nm. What refractive index does that correspond to?

Initial response: Let's find out the refractive index of TiO2 at 650 nm. I'll consult the materials expert for accurate data.\n\n<tool>materials_chat\nCould you provide the refractive index of TiO2 at a wavelength of 650 nm?\n</tool>"

Tool response: The estimated refractive index of TiO2 at a wavelength of 650 nm (0.65 μm) is approximately 2.5774.

NOTE THE USE OF <chat> TAGS RIGHT AWAY TO RESPOND TO THE USER:
Final response to user: <chat>Great! The refractive index of TiO2 at 650 nm is approximately 2.5774. \n\nWith this information, we can proceed with the design of your deflector. Could you please provide the remaining details for the design:\n1. The length of the deflector.\n2. The incident angles of the light in degrees.\n3. The deflection angles in degrees.\n4. The thickness of the deflector.\n\nOnce I have these details, I'll be able to help you further!</chat>



3. Calculation problem - think then use tools:
  I need to calculate the focal length based on these parameters.
  <tool>scientific_compute
  import numpy as np
  lens_diameter = 100e-6
```

```
  focal_length = 80e-6
  NA = lens_diameter / (2 * focal_length)
  print(f'NA: {NA}')
  </tool>

4. For neural network design (return the text you receive wrapped in <tool>neural_design </tool> tags):
<tool>neural_design
design_metalens(refractive_indices=[2.7], lens_diameter=[100e-6], focal_lengths=[200e-6], focal_x_offsets=[0], thickness=[500e-9], operating_wavelengths=[800e-9])
</tool>

5. Fix errors:
The error indicates that the solution was returned in a different format than expected. I have to first access the dictonary in the list.

6. Provide final answer:
<chat>
Based on my analysis, I recommend using a TiO2 metalens with these parameters:
- Diameter: 200 µm
- Focal length: 500 µm
- Thickness: 600 nm

Would you like me to help you design a metalens with these parameters?
</chat>

CONVERSATION GUIDELINES:
- Use <chat> tags for ALL user-facing responses, clarification, and missing details/parameters
- Maintain an engaging, natural conversational style
- Only switch to detailed step-by-step thinking for complex technical problems--but remember to switch back to conversation mode when done thinking!
- Remember that without <chat> tags, the user cannot see your response

IMPORTANT: Any text not wrapped in tags will be treated as your internal thoughts and planning. Only text within <chat> tags will be shown to the user.
Make sure your response makes sense to the user based on their last message.

The available tools are:
- Scientific computing: <tool>scientific_compute</tool>
- Symbolic solving: <tool>symbolic_solve</tool>
- Neural design: <tool>neural_design</tool>
- Materials expert chat: <tool>materials_chat</tool>
```

Furthermore, design quality recognition is enabled using the following message to the Design Agent after any optimization results are complete, along with the characterization image results:

```
This message is from the optimization assistant; your response will be to the user. Here are the farfield
    ↪ plots from the design. Analyze these images and provide feedback on the focusing quality, efficiency,
    ↪ and any artifacts or issues you observe. Note that negative deflection angles should be to the left,
    ↪ and positive deflection angles should be to the right. If the design is poor, one thing you can
    ↪ suggest is to increase the length of device, and/or any other parameters that you think might help. If
    ↪ the design is a draft and of decent quality, offer to optimize again using more computational
    ↪ resources for the best possible result. Respond to the user using <chat> tags and make sure to include
    ↪ the GDS file in the response.
```

Captions for Movies S1 to S2

Movie S1: Screen recording of design drafting using MetaChat. An example of a design flow between a designer and MetaChat is depicted, focused on fast, draft-quality designs. A Bragg grating calculation, deflector, and metalens design capabilities are depicted for a wide range of design parameters, including different materials and wavelengths. Scientific computing tool use, AIM Materials Expert Agent collaboration, and reasoning with deflector and metalens optimization in the loop are showcased in the example.

Movie S2: Screen recording of production design optimization using MetaChat. An example design flow between a designer and MetaChat is depicted, following up on the draft-quality designs from Movie S1 with high-quality optimizations of the same design problems.

Captions for Data S1 to S5

Data S1: Stanford nanophotonics evaluation benchmark. A benchmark of question-answer problems for photonics and optics design agents, comprised of 101 diverse problems in photonics, electromagnetics, and optical engineering, containing five categories: Direct Calculation, Multi-Step Calculation, Material Search, Direct Function Call, and Multi-Step Function Call. The benchmark is stored in JSON format.

Data S2: AIM Design Agent ablation study conversation logs. The internal monologues of the AIM Design Agent models presented in Fig. 2 of the main text, for each of the problems in the Stanford photonics benchmark. The data is stored in JSON format.

Data S3: AIM Materials Expert Agent ablation study internal monologue logs. The internal monologues of the AIM Materials Expert Agents engaged by the models from Fig. 2 of the main text with the capability to do so. The data is stored in JSON format.

Data S4: FiLM WaveY-Net training and test set metadata. The 300,000-element training and test set metadata is stored in a single database table. For each of the elements, the metadata includes grayscale temperature, permittivity, structure thickness, length, number of ridges, and the illuminating source angle and wavelength. These cover the range of data from Fig. 3C of the main text. The data is stored in Parquet file format.

Data S5: AIM Design Agent ablation study grading record. The automated grading results for each of the models in the ablation study from Fig. 2 of the main text. This includes agent solutions, grader-extracted answers, grading explanations, and solution approach feedback.

REFERENCES AND NOTES

1. G. Kim, Y. Kim, J. Yun, S.-W. Moon, S. Kim, J. Kim, J. Park, T. Badloe, I. Kim, J. Rho, Metasurface-driven full-space structured light for three-dimensional imaging. *Nat. Commun.* **13**, 5920 (2022).

2. E. Tseng, S. Colburn, J. Whitehead, L. Huang, S.-H. Baek, A. Majumdar, F. Heide, Neural nano-optics for high-quality thin lens imaging. *Nat. Commun.* **12**, 6493 (2021).

3. M. Gopakumar, G.-Y. Lee, S. Choi, B. Chao, Y. Peng, J. Kim, G. Wetzstein, Full-colour 3D holographic augmented-reality displays with metasurface waveguides. *Nature* **629**, 791–797 (2024).

4. W.-J. Joo, J. Kyoung, M. Esfandyarpour, S.-H. Lee, H. Koo, S. Song, Y.-N. Kwon, S. H. Song, J. C. Bae, A. Jo, M.-J. Kwon, S. H. Han, S.-H. Kim, S. Hwang, M. L. Brongersma, Metasurface-driven OLED displays beyond 10,000 pixels per inch. *Science* **370**, 459–463 (2020).

5. I. Kim, W.-S. Kim, K. Kim, M. A. Ansari, M. Q. Mehmood, T. Badloe, Y. Kim, J. Gwak, H. Lee, Y.-K. Kim, J. Rho, Holographic metasurface gas sensors for instantaneous visual alarms. *Sci Adv.* **7**, eabe9943 (2021).

6. Q. Guo, Z. Shi, Y.-W. Huang, E. Alexander, C.-W. Qiu, F. Capasso, T. Zickler, Compact single-shot metalens depth sensors inspired by eyes of jumping spiders. *Proc. Natl. Acad. Sci. U.S.A.* **116**, 22959–22965 (2019).

7. N. Yu, F. Capasso, Flat optics with designer metasurfaces. *Nat. Mater.* **13**, 139–150 (2014).

8. W. T. Chen, A. Y. Zhu, F. Capasso, Flat optics with dispersion-engineered metasurfaces. *Nat. Rev. Mater.* **5**, 604–620 (2020).

9. X. Ni, A. V. Kildishev, V. M. Shalaev, Metasurface holograms for visible light. *Nat. Commun.* **4**, 2807 (2013).

10. X. Lin, Y. Rivenson, N. T. Yardimci, M. Veli, Y. Luo, M. Jarrahi, A. Ozcan, All-optical machine learning using diffractive deep neural networks. *Science* **361**, 1004–1008 (2018).

11. N. Mohammadi Estakhri, B. Edwards, N. Engheta, Inverse-designed metastructures that solve equations. *Science* **363**, 1333–1338 (2019).

12. J. Jiang, M. Chen, J. A. Fan, Deep neural networks for the evaluation and design of photonic devices. *Nat. Rev. Mater.* **6**, 679–700 (2021).

13. W. Ma, Z. Liu, Z. A. Kudyshev, A. Boltasseva, W. Cai, Y. Liu, Deep learning for the design of photonic structures. *Nat. Photon.* **15**, 77–90 (2021).

14. Z. Li, R. Pestourie, Z. Lin, S. G. Johnson, F. Capasso, Empowering metasurfaces with inverse design: Principles and applications. *ACS Photon.* **9**, 2178–2192 (2022).

15. Z. Kuang, O. D. Miller, Computational bounds to light-matter interactions via local conservation laws. *Phys. Rev. Lett.* **125**, 263607 (2020).

16. J. Jiang, J. A. Fan, Global optimization of dielectric metasurfaces using a physics-driven neural network. *Nano Lett.* **19**, 5366–5372 (2019).

17. J. Jiang, J. A. Fan, Simulator-based training of generative neural networks for the inverse design of metasurfaces. *Nanophotonics* **9**, 1059–1069 (2020).

18. Z. A. Kudyshev, A. V. Kildishev, V. M. Shalaev, A. Boltasseva, Machine learning-assisted global optimization of photonic devices. *Nanophotonics* **10**, 371–383 (2020).

19. G. Arya, W. F. Li, C. Roques-Carmes, M. Soljačić, S. G. Johnson, Z. Lin, End-to-end optimization of metasurfaces for imaging with compressed sensing. *ACS Photon.* **11**, 2077–2087 (2024).

20. R. E. Christiansen, O. Sigmund, Inverse design in photonics by topology optimization: Tutorial. *J. Opt. Soc. Am. B* **38**, 496 (2021).

21. G. Angeris, J. Vučković, S. Boyd, Heuristic methods and performance bounds for photonic design. *Opt. Express* **29**, 2827–2854 (2021).

22. J. R. Thompson, H. D. Nelson-Quillin, E. J. Coyle, J. P. Vernon, E. S. Harper, M. S. Mills, Particle swarm optimization of polymer-embedded broadband metasurface reflectors. *Opt. Express* **29**, 43421 (2021).

23. V. Egorov, M. Eitan, J. Scheuer, Genetically optimized all-dielectric metasurfaces. *Opt. Express* **25**, 2583 (2017), 2593.

24. S. Jafar-Zanjani, S. Inampudi, H. Mosallaei, Adaptive genetic algorithm for optical metasurfaces design. *Sci. Rep.* **8**, 11040 (2018).

25. Z. Yu, H. Cui, X. Sun, Genetically optimized on-chip wideband ultracompact reflectors and Fabry-Perot cavities. *Photon. Res.* **5**, B15 (2017).

26. O. D. Miller, "Photonic design: From fundamental solar cell physics to computational inverse design," Ph.D. thesis, University of California, Berkeley (2012).

27. D. Sell, J. Yang, S. Doshay, R. Yang, J. A. Fan, Large-angle, multifunctional metagratings based on freeform multimode geometries. *Nano Lett.* **17**, 3752–3757 (2017).

28. R. Pestourie, C. Pérez-Arancibia, Z. Lin, W. Shin, F. Capasso, S. G. Johnson, Inverse design of large-area metasurfaces. *Opt. Express* **26**, 33732–33747 (2018).

29. A. Y. Piggott, J. Petykiewicz, L. Su, J. Vučković, Fabrication-constrained nanophotonic inverse design. *Sci. Rep.* **7**, 1786 (2017).

30. M. Mansouree, A. McClung, S. Samudrala, A. Arbabi, Large-scale parametrized metasurface design using adjoint optimization. *ACS Photon.* **8**, 455–463 (2021).

31. S. Colburn, A. Zhan, A. Majumdar, Metasurface optics for full-color computational imaging. *Sci. Adv.* **4**, eaar2114 (2018).

32. C. Yeung, B. Pham, R. Tsai, K. T. Fountaine, A. P. Raman, DeepAdjoint: An all-in-one photonic inverse design framework integrating data-driven machine learning with optimization algorithms. *ACS Photon.* **10**, 884–891 (2023).

33. R. Lupoiu, J. A. Fan, “Machine learning advances in computational electromagnetics” in *Advances in Electromagnetics Empowered by Artificial Intelligence and Deep Learning*, S. Campbell, D. Werner, Eds. (Wiley, ed. 1, 2023), pp. 225–252.

34. M. Raissi, P. Perdikaris, G. Karniadakis, Physics-informed neural networks: A deep learning framework for solving forward and inverse problems involving nonlinear partial differential equations. *J. Comput. Phys.* **378**, 686–707 (2019).

35. X. Meng, Z. Li, D. Zhang, G. E. Karniadakis, PPINN: Parareal physics-informed neural network for time-dependent PDEs. *Comput. Methods Appl. Mech. Eng.* **370**, 113250 (2020).

36. E. Kharazmi, Z. Zhang, G. E. Karniadakis, hp-VPINNs: Variational physics-informed neural networks with domain decomposition. *Comput. Methods Appl. Mech. Eng.* **374**, 113547 (2021).

37. L. Lu, R. Pestourie, W. Yao, Z. Wang, F. Verdugo, S. G. Johnson, Physics-informed neural networks with hard constraints for inverse design. *SIAM J. Sci. Comput.* **43**, B1105–B1132 (2021).

38. L. Yuan, Y.-Q. Ni, X.-Y. Deng, S. Hao, A-PINN: Auxiliary physics informed neural networks for forward and inverse problems of nonlinear integro-differential equations. *J. Comput. Phys.* **462**, 111260 (2022).

39. Z. Li, N. Kovachki, K. Azizzadenesheli, B. Liu, K. Bhattacharya, A. Stuart, A. Anandkumar, Fourier neural operator for parametric partial differential equations. arXiv:2010.08895 (2021).

40. N. Kovachki, S. Lanthaler, S. Mishra, On universal approximation and error bounds for Fourier neural operators. *J. Mach. Learn. Res.* **22**, 1–76 (2021).

41. G. Wen, Z. Li, K. Azizzadenesheli, A. Anandkumar, S. M. Benson, U-FNO—An enhanced Fourier neural operator-based deep-learning model for multiphase flow. *Adv. Water Resour.* **163**, 104180 (2022).

42. Z. Li, D. Z. Huang, B. Liu, Fourier neural operator with learned deformations for PDEs on general geometries. *J. Mach. Learn. Res.* **24**, 1–26 (2023).

43. M. Chen, R. Lupoiu, C. Mao, D.-H. Huang, J. Jiang, P. Lalanne, J. A. Fan, High speed simulation and freeform optimization of nanophotonic devices with physics-augmented deep learning. *ACS Photon.* **9**, 3110–3123 (2022).

44. C. Mao, R. Lupoiu, T. Dai, M. Chen, J. A. Fan, Towards general neural surrogate solvers with specialized neural accelerators. arXiv:2405.02351 (2024).

45. P. R. Wiecha, O. L. Muskens, Deep learning meets nanophotonics: A generalized accurate predictor for near fields and far fields of arbitrary 3D nanostructures. *Nano Lett.* **20**, 329–338 (2020).

46. M. Zhelyeznyakov, J. Fröch, A. Wirth-Singh, J. Noh, J. Rho, S. Brunton, A. Majumdar, Large area optimization of meta-lens via data-free machine learning. *Commun. Eng.* **2**, 60 (2023).

47. D. Lu, Y. Deng, J. M. Malof, W. J. Padilla, Learning electromagnetic metamaterial physics with ChatGPT. *IEEE Access* **13**, 51513–51526 (2025).

48. M. Kim, H. Park, J. Shin, Nanophotonic device design based on large language models: Multilayer and metasurface examples. *Nanophotonics* **14**, 1273–1282 (2025).

49. T. Ma, H. Wang, L. J. Guo, OptoGPT: A foundation model for inverse design in optical multilayer thin film structures. *Opto Electron. Adv.* **7**, 240062–240062 (2024).

50. A. Ghafarollahi, M. J. Buehler, AtomAgents: Alloy design and discovery through physics-aware multi-modal multi-agent artificial intelligence. arXiv:2407.10022 (2024).

51. X. Jiang, M. Zhang, Y. Song, Y. Zhang, Y. Wang, C. Ju, D. Wang, OptiComm-GPT: A GPT-based versatile research assistant for optical fiber communication systems. *Opt. Express* **32**, 20776–20796 (2024).

52. R. Li, C. Zhang, S. Mao, H. Huang, M. Zhong, Y. Cui, X. Zhou, F. Yin, S. Theodoridis, Z. Zhang, From English to PCSEL: LLM helps design and optimize photonic crystal surface emitting lasers. arXiv:2104.12145v2 (2023).

53. A. Ghafarollahi, M. J. Buehler, SciAgents: Automating scientific discovery through bioinspired multi-agent intelligent graph reasoning. *Adv. Mater.* **37**, 2413523 (2024).

54. A. Bandura, Social cognitive theory: An agentic perspective. *Annu. Rev. Psychol.* **52**, 1–26 (2001).

55. S. Yao, J. Zhao, D. Yu, N. Du, I. Shafran, K. Narasimhan, Y. Cao, ReAct: Synergizing reasoning and acting in language models. arXiv:2210.03629 (2023).

56. D. Fu, J. Huang, S. Lu, G. Dong, Y. Wang, K. He, W. Xu, PreAct: Prediction enhances agent's planning ability. arXiv:2402.11534 (2024).

57. K. Swanson, W. Wu, N. L. Bulaong, J. E. Pak, J. Zou, The virtual lab: AI agents design new SARS-CoV-2 nanobodies with experimental validation. bioRxiv 623004 [Preprint] (2024); https://doi.org/10.1101/2024.11.11.623004.

58. A. Vaswani, N. Shazeer, N. Parmar, J. Uszkoreit, L. Jones, A. N. Gomez, L. Kaiser, I. Polosukhin, Attention is all you need. arXiv:1706.03762 (2017).

59. T. B. Brown, B. Mann, N. Ryder, M. Subbiah, J. Kaplan, P. Dhariwal, A. Neelakantan, P. Shyam, G. Sastry, A. Askell, S. Agarwal, A. Herbert-Voss, G. Krueger, T. Henighan, R. Child, A. Ramesh, D. M. Ziegler, J. Wu, C. Winter, C. Hesse, M. Chen, E. Sigler, M. Litwin, S. Gray, B. Chess, J. Clark, C. Berner, S. McCandlish, A. Radford, I. Sutskever, D. Amodei, Language models are few-shot learners. arXiv:2005.14165 (2020).

60. L. McInnes, J. Healy, J. Melville, UMAP: Uniform Manifold Approximation and Projection for dimension reduction. arXiv:1802.03426 (2020).

61. J. Wei, X. Wang, D. Schuurmans, M. Bosma, B. Ichter, F. Xia, E. H. Chi, Q. V. Le, D. Zhou, Chain-of-thought prompting elicits reasoning in large language models. arXiv:2201.11903 (2022).

62. W.-L. Chiang, L. Zheng, Y. Sheng, A. N. Angelopoulos, T. Li, D. Li, B. Zhu, H. Zhang, M. I. Jordan, J. E. Gonzalez, I. Stoica, Chatbot Arena: An open platform for evaluating LLMs by human preference. arXiv:2403.04132v1 (2024).

63. L. Zheng, W.-L. Chiang, Y. Sheng, T. Li, S. Zhuang, Z. Wu, Y. Zhuang, Z. Li, Z. Lin, E. P. Xing, J. E. Gonzalez, I. Stoica, H. Zhang, LMSYS-CHAT-1M: A large-scale real-world LLM conversation dataset. arXiv:2309.11998 (2024).

64. T. Phan, D. Sell, E. W. Wang, S. Doshay, K. Edee, J. Yang, J. A. Fan, High-efficiency, large-area, topology-optimized metasurfaces. *Light Sci Appl* **8**, 48 (2019).

65. T. W. Hughes, I. A. D. Williamson, M. Minkov, S. Fan, Forward-mode differentiation of Maxwell's equations. *ACS Photon.* **6**, 3010–3016 (2019).

66. J. Lim, D. Psaltis, MaxwellNet: Physics-driven deep neural network training based on Maxwell's equations. *APL Photon.* **7**, 011301 (2022).

67. E. Perez, F. Strub, H. de Vries, V. Dumoulin, A. Courville, FiLM: Visual Reasoning with a general conditioning layer. arXiv:1709.07871 (2017).

68. D. P. Kingma, J. Ba, Adam: A Method for Stochastic Optimization. arXiv:1412.6980 (2015).

69. B. H. Chen, P. C. Wu, V.-C. Su, Y.-C. Lai, C. H. Chu, I. C. Lee, J.-W. Chen, Y. H. Chen, Y.-C. Lan, C.-H. Kuan, D. P. Tsai, GaN metalens for pixel-level full-color routing at visible light. *Nano Lett.* **17**, 6345–6352 (2017).

70. T. Choi, C. Choi, J. Bang, Y. Kim, H. Son, C. Kim, J. Jang, Y. Jeong, B. Lee, Multiwavelength achromatic deflector in the visible using a single-layer freeform metasurface. *Nano Lett.* **24**, 10980–10986 (2024).

71. H. W. Chung, L. Hou, S. Longpre, B. Zoph, Y. Tay, W. Fedus, Y. Li, X. Wang, M. Dehghani, S. Brahma, A. Webson, S. S. Gu, Z. Dai, M. Suzgun, X. Chen, A. Chowdhery, A. Castro-Ros, M. Pellat, K. Robinson, D. Valter, S. Narang, G. Mishra, A. Yu, V. Zhao, Y. Huang, A. Dai, H.

Yu, S. Petrov, E. H. Chi, J. Dean, J. Devlin, A. Roberts, D. Zhou, Q. V. Le, J. Wei, Scaling instruction-finetuned language models. arXiv:2210.11416 (2022).

72. L. Ouyang, J. Wu, X. Jiang, D. Almeida, C. L. Wainwright, P. Mishkin, C. Zhang, S. Agarwal, K. Slama, A. Ray, J. Schulman, J. Hilton, F. Kelton, L. Miller, M. Simens, A. Askell, P. Welinder, P. Christiano, J. Leike, R. Lowe, Training language models to follow instructions with human feedback. arXiv:2203.02155 (2022).

73. H. Touvron, T. Lavril, G. Izacard, X. Martinet, M.-A. Lachaux, T. Lacroix, B. Rozière, N. Goyal, E. Hambro, F. Azhar, A. Rodriguez, A. Joulin, E. Grave, G. Lample, LLaMA: Open and efficient foundation language models. arXiv:2302.13971 (2023).

74. Z. Shao, P. Wang, Q. Zhu, R. Xu, J. Song, X. Bi, H. Zhang, M. Zhang, Y. K. Li, Y. Wu, D. Guo, DeepSeekMath: Pushing the limits of mathematical reasoning in open language models. arXiv:2402.03300 (2024).

75. DeepSeek-AI, D. Guo, D. Yang, H. Zhang, J. Song, R. Zhang, R. Xu, Q. Zhu, S. Ma, P. Wang, X. Bi, X. Zhang, X. Yu, Y. Wu, Z.F. Wu, Z. Gou, Z. Shao, Z. Li, Z. Gao, A. Liu, B. Xue, B. Wang, B. Wu, B. Feng, C. Lu, C. Zhao, C. Deng, C. Zhang, C. Ruan, D. Dai, D. Chen, D. Ji, E. Li, F. Lin, F. Dai, F. Luo, G. Hao, G. Chen, G. Li, H. Zhang, H. Bao, H. Xu, H. Wang, H. Ding, H. Xin, H. Gao, H. Qu, H. Li, J. Guo, J. Li, J. Wang, J. Chen, J. Yuan, J. Qiu, J. Li, J.L. Cai, J. Ni, J. Liang, J. Chen, K. Dong, K. Hu, K. Gao, K. Guan, K. Huang, K. Yu, L. Wang, L. Zhang, L. Zhao, L. Wang, L. Zhang, L. Xu, L. Xia, M. Zhang, M. Zhang, M. Tang, M. Li, M. Wang, M. Li, N. Tian, P. Huang, P. Zhang, Q. Wang, Q. Chen, Q. Du, R. Ge, R. Zhang, R. Pan, R. Wang, R.J. Chen, R.L. Jin, R. Chen, S. Lu, S. Zhou, S. Chen, S. Ye, S. Wang, S. Yu, S. Zhou, S. Pan, S.S. Li, S. Zhou, S. Wu, S. Ye, T. Yun, T. Pei, T. Sun, T. Wang, W. Zeng, W. Zhao, W. Liu, W. Liang, W. Gao, W. Yu, W. Zhang, W.L. Xiao, W. An, X. Liu, X. Wang, X. Chen, X. Nie, X. Cheng, X. Liu, X. Xie, X. Liu, X. Yang, X. Li, X. Su, X. Lin, X.Q. Li, X. Jin, X. Shen, X. Chen, X. Sun, X. Wang, X. Song, X. Zhou, X. Wang, X. Shan, Y.K. Li, Y.Q. Wang, Y.X. Wei, Y. Zhang, Y. Xu, Y. Li, Y. Zhao, Y. Sun, Y. Wang, Y. Yu, Y. Zhang, Y. Shi, Y. Xiong, Y. He, Y. Piao, Y. Wang, Y. Tan, Y. Ma, Y. Liu, Y. Guo, Y. Ou, Y. Wang, Y. Gong, Y. Zou, Y. He, Y. Xiong, Y. Luo, Y. You, Y. Liu, Y. Zhou, Y.X. Zhu, Y. Xu, Y. Huang, Y. Li, Y. Zheng, Y. Zhu,

Y. Ma, Y. Tang, Y. Zha, Y. Yan, Z.Z. Ren, Z. Ren, Z. Sha, Z. Fu, Z. Xu, Z. Xie, Z. Zhang, Z. Hao, Z. Ma, Z. Yan, Z. Wu, Z. Gu, Z. Zhu, Z. Liu, Z. Li, Z. Xie, Z. Song, Z. Pan, Z. Huang, Z. Xu, Z. Zhang, Z. Zhang, DeepSeek-R1: Incentivizing reasoning capability in LLMs via reinforcement learning. arXiv:2501.12948 (2025).

76. S. Yin, C. Fu, S. Zhao, K. Li, X. Sun, T. Xu, E. Chen, A survey on multimodal large language models. *Natl. Sci. Rev.* **11**, nwae403 (2024).

77. OpenAI, J. Achiam, S. Adler, S. Agarwal, L. Ahmad, I. Akkaya, F. L. Aleman, D. Almeida, J. Altenschmidt, S. Altman, S. Anadkat, R. Avila, I. Babuschkin, S. Balaji, V. Balcom, P. Baltescu, H. Bao, M. Bavarian, J. Belgum, I. Bello, J. Berdine, G. Bernadett-Shapiro, C. Berner, L. Bogdonoff, O. Boiko, M. Boyd, A.-L. Brakman, G. Brockman, T. Brooks, M. Brundage, K. Button, T. Cai, R. Campbell, A. Cann, B. Carey, C. Carlson, R. Carmichael, B. Chan, C. Chang, F. Chantzis, D. Chen, S. Chen, R. Chen, J. Chen, M. Chen, B. Chess, C. Cho, C. Chu, H. W. Chung, D. Cummings, J. Currier, Y. Dai, C. Decareaux, T. Degry, N. Deutsch, D. Deville, A. Dhar, D. Dohan, S. Dowling, S. Dunning, A. Ecoffet, A. Eleti, T. Eloundou, D. Farhi, L. Fedus, N. Felix, S. P. Fishman, J. Forte, I. Fulford, L. Gao, E. Georges, C. Gibson, V. Goel, T. Gogineni, G. Goh, R. Gontijo-Lopes, J. Gordon, M. Grafstein, S. Gray, R. Greene, J. Gross, S. S. Gu, Y. Guo, C. Hallacy, J. Han, J. Harris, Y. He, M. Heaton, J. Heidecke, C. Hesse, A. Hickey, W. Hickey, P. Hoeschele, B. Houghton, K. Hsu, S. Hu, X. Hu, J. Huizinga, S. Jain, S. Jain, J. Jang, A. Jiang, R. Jiang, H. Jin, D. Jin, S. Jomoto, B. Jonn, H. Jun, T. Kaftan, Ł. Kaiser, A. Kamali, I. Kanitscheider, N. S. Keskar, T. Khan, L. Kilpatrick, J. W. Kim, C. Kim, Y. Kim, J. H. Kirchner, J. Kiros, M. Knight, D. Kokotajlo, Ł. Kondraciuk, A. Kondrich, A. Konstantinidis, K. Kosic, G. Krueger, V. Kuo, M. Lampe, I. Lan, T. Lee, J. Leike, J. Leung, D. Levy, C. M. Li, R. Lim, M. Lin, S. Lin, M. Litwin, T. Lopez, R. Lowe, P. Lue, A. Makanju, K. Malfacini, S. Manning, T. Markov, Y. Markovski, B. Martin, K. Mayer, A. Mayne, B. M. Grew, Scott Mayer Mc Kinney, C. M. Leavey, P. M. Millan, J. M. Neil, D. Medina, A. Mehta, J. Menick, L. Metz, A. Mishchenko, P. Mishkin, V. Monaco, E. Morikawa, D. Mossing, T. Mu, M. Murati, O. Murk, D. Mély, A. Nair, R. Nakano, R. Nayak, A. Neelakantan, R. Ngo, H. Noh, L. Ouyang, C. O.'K, J. Pachocki, A. Paino, J. Palermo, A. Pantuliano, G. Parascandolo, J. Parish, E. Parparita, A. Passos, M. Pavlov, A. Peng, A. Perelman, Filipe de Avila Belbute Peres, M. Petrov, Henrique Ponde de Oliveira Pinto, M. Pokorny, M. Pokrass, V. H. Pong, T. Powell, A. Power, B. Power,

E. Proehl, R. Puri, A. Radford, J. Rae, A. Ramesh, C. Raymond, F. Real, K. Rimbach, C. Ross, B. Rotsted, H. Roussez, N. Ryder, M. Saltarelli, T. Sanders, S. Santurkar, G. Sastry, H. Schmidt, D. Schnurr, J. Schulman, D. Selsam, K. Sheppard, T. Sherbakov, J. Shieh, S. Shoker, P. Shyam, S. Sidor, E. Sigler, M. Simens, J. Sitkin, K. Slama, I. Sohl, B. Sokolowsky, Y. Song, N. Staudacher, F. P. Such, N. Summers, I. Sutskever, J. Tang, N. Tezak, M. B. Thompson, P. Tillet, A. Tootoonchian, E. Tseng, P. Tuggle, N. Turley, J. Tworek, Juan Felipe Cerón Uribe, A. Vallone, A. Vijayvergiya, C. Voss, C. Wainwright, J. J. Wang, A. Wang, B. Wang, J. Ward, J. Wei, C. J. Weinmann, A. Welihinda, P. Welinder, J. Weng, L. Weng, M. Wiethoff, D. Willner, C. Winter, S. Wolrich, H. Wong, L. Workman, S. Wu, J. Wu, M. Wu, K. Xiao, T. Xu, S. Yoo, K. Yu, Q. Yuan, W. Zaremba, R. Zellers, C. Zhang, M. Zhang, S. Zhao, T. Zheng, J. Zhuang, W. Zhuk, B. Zoph, GPT-4 technical report. arXiv:2303.08774 (2024).

78. J. Jiang, R. Lupoiu, E. W. Wang, D. Sell, J. Paul Hugonin, P. Lalanne, J. A. Fan, MetaNet: A new paradigm for data sharing in photonics research. *Opt. Express* **28**, 13670–13681 (2020).

79. M. F. Schubert, invrs-gym: A toolkit for nanophotonic inverse design research. arXiv:2410.24132 (2024).

80. A. Srivastava, A. Rastogi, A. Rao, Abu Awal Md Shoeb, A. Abid, A. Fisch, A. R. Brown, A. Santoro, A. Gupta, A. Garriga-Alonso, A. Kluska, A. Lewkowycz, A. Agarwal, A. Power, A. Ray, A. Warstadt, A. W. Kocurek, A. Safaya, A. Tazarv, A. Xiang, A. Parrish, A. Nie, A. Hussain, A. Askell, A. Dsouza, A. Slone, A. Rahane, A. S. Iyer, A. Andreassen, A. Madotto, A. Santilli, A. Stuhlmüller, A. Dai, A. La, A. Lampinen, A. Zou, A. Jiang, A. Chen, A. Vuong, A. Gupta, A. Gottardi, A. Norelli, A. Venkatesh, A. Gholamidavoodi, A. Tabassum, A. Menezes, A. Kirubarajan, A. Mullokandov, A. Sabharwal, A. Herrick, A. Efrat, A. Erdem, A. Karakaş, B. Ryan Roberts, B. S. Loe, B. Zoph, B. Bojanowski, B. Özyurt, B. Hedayatnia, B. Neyshabur, B. Inden, B. Stein, B. Ekmekci, B. Y. Lin, B. Howald, B. Orinion, C. Diao, C. Dour, C. Stinson, C. Argueta, C. F. Ramírez, C. Singh, C. Rathkopf, C. Meng, C. Baral, C. Wu, C. Callison-Burch, C. Waites, C. Voigt, C. D. Manning, C. Potts, C. Ramirez, C. E. Rivera, C. Siro, C. Raffel, C. Ashcraft, C. Garbacea, D. Sileo, D. Garrette, D. Hendrycks, D. Kilman, D. Roth, D. Freeman, D. Khashabi, D. Levy, D. M. González, D. Perszyk, D. Hernandez, D. Chen, D. Ippolito, D. Gilboa, D. Dohan, D. Drakard, D. Jurgens, D. Datta, D. Ganguli, D. Emelin, D. Kleyko, D. Yuret, D.

Chen, D. Tam, D. Hupkes, D. Misra, D. Buzan, D. C. Mollo, D. Yang, D.-H. Lee, D. Schrader, E. Shutova, E. D. Cubuk, E. Segal, E. Hagerman, E. Barnes, E. Donoway, E. Pavlick, E. Rodola, E. Lam, E. Chu, E. Tang, E. Erdem, E. Chang, E. A. Chi, E. Dyer, E. Jerzak, E. Kim, E. E. Manyasi, E. Zheltonozhskii, F. Xia, F. Siar, F. Martínez-Plumed, F. Happé, F. Chollet, F. Rong, G. Mishra, G. I. Winata, G. de Melo, G. Kruszewski, G. Parascandolo, G. Mariani, G. Wang, G. Jaimovitch-López, G. Betz, G. Gur-Ari, H. Galijasevic, H. Kim, H. Rashkin, H. Hajishirzi, H. Mehta, H. Bogar, H. Shevlin, H. Schütze, H. Yakura, H. Zhang, H. M. Wong, I. Ng, I. Noble, J. Jumelet, J. Geissinger, J. Kernion, J. Hilton, J. Lee, J. F. Fisac, J. B. Simon, J. Koppel, J. Zheng, J. Zou, J. Kocoń, J. Thompson, J. Wingfield, J. Kaplan, J. Radom, J. Sohl-Dickstein, J. Phang, J. Wei, J. Yosinski, J. Novikova, J. Bosscher, J. Marsh, J. Kim, J. Taal, J. Engel, J. Alabi, J. Xu, J. Song, J. Tang, J. Waweru, J. Burden, J. Miller, J. U. Balis, J. Batchelder, J. Berant, J. Frohberg, J. Rozen, J. Hernandez-Orallo, J. Boudeman, J. Guerr, J. Jones, J. B. Tenenbaum, J. S. Rule, J. Chua, K. Kanclerz, K. Livescu, K. Krauth, K. Gopalakrishnan, K. Ignatyeva, K. Markert, K. D. Dhole, K. Gimpel, K. Omondi, K. Mathewson, K. Chiafullo, K. Shkaruta, K. Shridhar, K. M. Donell, K. Richardson, L. Reynolds, L. Gao, L. Zhang, L. Dugan, L. Qin, L. Contreras-Ochando, L.-P. Morency, L. Moschella, L. Lam, L. Noble, L. Schmidt, L. He, L. O. Colón, L. Metz, L. K. Şenel, M. Bosma, M. Sap, Maartje ter Hoeve, M. Farooqi, M. Faruqui, M. Mazeika, M. Baturan, M. Marelli, M. Maru, Maria Jose Ramírez Quintana, M. Tolkiehn, M. Giulianelli, M. Lewis, M. Potthast, M. L. Leavitt, M. Hagen, M. Schubert, M. O. Baitemirova, M. Arnaud, M. M. Elrath, M. A. Yee, M. Cohen, M. Gu, M. Ivanitskiy, M. Starritt, M. Strube, M. Swędrowski, M. Bevilacqua, M. Yasunaga, M. Kale, M. Cain, M. Xu, M. Suzgun, M. Walker, M. Tiwari, M. Bansal, M. Aminnaseri, M. Geva, M. Gheini, Mukund Varma T, N. Peng, N. A. Chi, N. Lee, Neta Gur-Ari Krakover, N. Cameron, N. Roberts, N. Doiron, N. Martinez, N. Nangia, N. Deckers, N. Muennighoff, N. S. Keskar, N. S. Iyer, N. Constant, N. Fiedel, N. Wen, O. Zhang, O. Agha, O. Elbaghdadi, O. Levy, O. Evans, Pablo Antonio Moreno Casares, P. Doshi, P. Fung, P. P. Liang, P. Vicol, P. Alipoormolabashi, P. Liao, P. Liang, P. Chang, P. Eckersley, P. M. Htut, P. Hwang, P. Miłkowski, P. Patil, P. Pezeshkpour, P. Oli, Q. Mei, Q. Lyu, Q. Chen, R. Banjade, R. E. Rudolph, R. Gabriel, R. Habacker, R. Risco, R. Millière, R. Garg, R. Barnes, R. A. Saurous, R. Arakawa, R. Raymaekers, R. Frank, R. Sikand, R. Novak, R. Sitelew, R. L. Bras, R. Liu, R. Jacobs, R. Zhang, R. Salakhutdinov, R. Chi, R. Lee, R. Stovall, R. Teehan, R. Yang, S. Singh, S. M. Mohammad, S. Anand, S. Dillavou, S. Shleifer, S. Wiseman, S. Gruetter, S. R.

Bowman, S. S. Schoenholz, S. Han, S. Kwatra, S. A. Rous, S. Ghazarian, S. Ghosh, S. Casey, S. Bischoff, S. Gehrmann, S. Schuster, S. Sadeghi, S. Hamdan, S. Zhou, S. Srivastava, S. Shi, S. Singh, S. Asaadi, S. S. Gu, S. Pachchigar, S. Toshniwal, S. Upadhyay, S. Debnath, S. Shakeri, S. Thormeyer, S. Melzi, S. Reddy, S. P. Makini, S.-H. Lee, S. Torene, S. Hatwar, S. Dehaene, S. Divic, S. Ermon, S. Biderman, S. Lin, S. Prasad, S. T. Piantadosi, S. M. Shieber, S. Misherghi, S. Kiritchenko, S. Mishra, T. Linzen, T. Schuster, T. Li, T. Yu, T. Ali, T. Hashimoto, T.-L. Wu, T. Desbordes, T. Rothschild, T. Phan, T. Wang, T. Nkinyili, T. Schick, T. Kornev, T. Tunduny, T. Gerstenberg, T. Chang, T. Neeraj, T. Khot, T. Shultz, U. Shaham, V. Misra, V. Demberg, V. Nyamai, V. Raunak, V. Ramasesh, V. U. Prabhu, V. Padmakumar, V. Srikumar, W. Fedus, W. Saunders, W. Zhang, W. Vossen, X. Ren, X. Tong, X. Zhao, X. Wu, X. Shen, Y. Yaghoobzadeh, Y. Lakretz, Y. Song, Y. Bahri, Y. Choi, Y. Yang, Y. Hao, Y. Chen, Y. Belinkov, Y. Hou, Y. Hou, Y. Bai, Z. Seid, Z. Zhao, Z. Wang, Z. J. Wang, Z. Wang, Z. Wu, Beyond the imitation game: Quantifying and extrapolating the capabilities of language models. arXiv:2206.04615 (2023).

81. J. Gottweis, W.-H. Weng, A. Daryin, T. Tu, A. Palepu, P. Sirkovic, A. Myaskovsky, F. Weissenberger, K. Rong, R. Tanno, K. Saab, D. Popovici, J. Blum, F. Zhang, K. Chou, A. Hassidim, B. Gokturk, A. Vahdat, P. Kohli, Y. Matias, A. Carroll, K. Kulkarni, N. Tomasev, V. Dhillon, E. D. Vaishnav, B. Lee, T. R. D. Costa, J. R. Penadés, G. Peltz, Y. Xu, A. Pawlosky, A. Karthikesalingam, V. Natarajan, Towards an AI co-scientist. arXiv:2502.18864 (2025).

82. L. M. Roch, F. Häse, C. Kreisbeck, T. Tamayo-Mendoza, L. P. E. Yunker, J. E. Hein, A. Aspuru-Guzik, ChemOS: Orchestrating autonomous experimentation *Sci. Robot.* **3**, eaat5559 (2018).

83. T. Dai, S. Vijayakrishnan, F. T. Szczypiński, J.-F. Ayme, E. Simaei, T. Fellowes, R. Clowes, L. Kotopanov, C. E. Shields, Z. Zhou, J. W. Ward, A. I. Cooper, Autonomous mobile robots for exploratory synthetic chemistry. *Nature* **635**, 890–897 (2024).

84. K. Hatakeyama-Sato, H. Ishikawa, S. Takaishi, Y. Igarashi, Y. Nabae, T. Hayakawa, Semiautomated experiment with a robotic system and data generation by foundation models for synthesis of polyamic acid particles. *Polymer J.* **56**, 977–986 (2024).

85. S. J. Woo, C. Huang, J. Savall, B. L. Conrad, J. Luo, M. J. Schnitzer, An autonomous robotic system for high-throughput phenotyping and behavioral studies of individual fruit flies. bioRxiv 607451 [Preprint] (2024); https://doi.org/10.1101/2024.08.21.607451.

86. M. Chen, J. Tworek, H. Jun, Q. Yuan, Henrique Ponde de Oliveira Pinto, J. Kaplan, H. Edwards, Y. Burda, N. Joseph, G. Brockman, A. Ray, R. Puri, G. Krueger, M. Petrov, H. Khlaaf, G. Sastry, P. Mishkin, B. Chan, S. Gray, N. Ryder, M. Pavlov, A. Power, L. Kaiser, M. Bavarian, C. Winter, P. Tillet, F. P. Such, D. Cummings, M. Plappert, F. Chantzis, E. Barnes, A. Herbert-Voss, W. H. Guss, A. Nichol, A. Paino, N. Tezak, J. Tang, I. Babuschkin, S. Balaji, S. Jain, W. Saunders, C. Hesse, A. N. Carr, J. Leike, J. Achiam, V. Misra, E. Morikawa, A. Radford, M. Knight, M. Brundage, M. Murati, K. Mayer, P. Welinder, B. M. Grew, D. Amodei, S. M. Candlish, I. Sutskever, W. Zaremba, Evaluating large language models trained on code. arXiv:2107.03374 (2021).

87. O. Ronneberger, P. Fischer, T. Brox, U-Net: Convolutional networks for biomedical image segmentation. arXiv:1505.04597 (2015).

88. S. Ioffe, C. Szegedy, Batch normalization: Accelerating deep network training by reducing internal covariate shift. arXiv:1502.03167 (2015).

89. A. L. Maas, A. Y. Hannun, A. Y. Ng, “Rectifier nonlinearities improve neural network acoustic models,” in *Proceedings of the 30th International Conference on Machine Learning* (Proceedings of Machine Learning Research, 2013).

90. K. He, X. Zhang, S. Ren, J. Sun, Deep residual learning for image recognition. arXiv:1512.03385 (2015).

91. M. Chen, J. Jiang, J. A. Fan, Design space reparameterization enforces hard geometric constraints in inverse-designed nanophotonic devices. *ACS Photon.* **7**, 3141–3151 (2020).

92. K. Yee, Numerical solution of initial boundary value problems involving maxwell’s equations in isotropic media. *IEEE Trans. Antennas Propag.* **14**, 302–307 (1966).

93. J. A. Stratton, L. J. Chu, Diffraction theory of electromagnetic waves. *Phys. Rev.* **56**, 99–107 (1939).

94. R. Lupoiu, Y. Shao, T. Dai, C. Mao, K. Edee, J. Fan, “Data and Code for MetaChat: A multi-agentic framework for real-time, autonomous freeform metasurface design” (2025); https://doi.org/10.5281/zenodo.15802727.

95. S. Kang, S. Uchida, B. K. Iwana, Tunable U-Net: Controlling image-to-image outputs using a tunable scalar value. *IEEE Access* **9**, 103279–103290 (2021).

96. V. Medvedev, A. Erdmann, A. Rosskopf, Physics-informed deep learning for 3D modeling of light diffraction from optical metasurfaces. *Opt. Express* **33**, 1371 (2025), 1384.

97. M. N. Polyanskiy, Refractiveindex.info database of optical constants. *Sci. Data* **11**, 94 (2024).

98. S. A. Cholewiak, P. Ipeirotis, V. Silva, A. Kannawadi, “SCHOLARLY: Simple access to Google Scholar authors and citation using Python” (2021); https://github.com/scholarly-python-package/scholarly.

99. M. N. Polyanskiy, “Refractive index database” (2025); https://refractiveindex.info.

100. S. J. Reddi, S. Kale, S. Kumar, On the convergence of Adam and beyond. arXiv:1904.09237 (2019).
